# Quality-of-life outcomes in older patients with early-stage rectal cancer receiving organ-preserving treatment with hypofractionated short-course radiotherapy followed by transanal endoscopic microsurgery (TREC): non-randomised registry of patients unsuitable for total mesorectal excision

**DOI:** 10.1016/S2666-7568(22)00239-2

**Published:** 2022-12

**Authors:** Alexandra Gilbert, Victoria Homer, Kristian Brock, Stephan Korsgen, Ian Geh, James Hill, Talvinder Gill, Paul Hainsworth, Matthew Tutton, Jim Khan, Jonathan Robinson, Mark Steward, Christopher Cunningham, Manjinder Kaur, Laura Magill, Ann Russell, Philip Quirke, Nicholas P West, David Sebag-Montefiore, Simon P Bach, TREC collaborators, Alan Beveridge, Bruce Levy, Kelly Handley, Gina Brown, Peter Antonio, Alex Vince, Nick Hilken, Chakanaka Sidile, Adrian Wilcockson, Richard Peto, Tom Crosby, Brendan Moran, Julie Olliff, Katti Ashok, Simone Slawik, Andrew Smethurst, Rajaram Sripadam, Veena Tagore, Monica Terlizzo, Bearn Philip, Robert Davies, Susan Dodd, Sharadah Essapen, Pasha Nisar, Alexandra Stewart, Jonathan Trickett, Bansal Ashish, Peter Billings, Palanichamy Chandran, Conor Corr, Edward Favill, Simon Gollins, Peter Marsh, Andrew Maw, Rakha Neupane, Ramesh Rajagopal, Rachel Cooper, John Griffith, Paul Hatfield, Andy Lowe, Julian Ostrowski, Jonathan Robinson, Rhian Simpson, Richard Adams, Robert Bleehen, Michael Davies, Meleri Morgan, Darren Boone, Nicola Lacey, Ian Seddon, Bruce Sizer, Helen Stunell, Shaobin Wu, Maher Hadaki, Dominic Blunt, Susan Cleator, Ara Darzi, Robert Goldin, Paul Ziprin, Mike Dobson, Mark Pitt, Shabbir Susnerwala, Deborah Williamson, Georgina Howarth, Stephen Lee, Paul Wright, Tim Hoare, Alan Horgan, Fiona McDonald, Stephanie Needham, John Scott, Timothy Simmons, Debashis Biswas, James Hernon, Gaurav Kapur, Sandeep Kapur, James Sington, Christopher Speakman, William Stebbings, Stuart Williams, Madhavi Adusumalli, Anil Agarwal, David Borowski, Dharmendra Garg, Talvinder Gill, Mohammed Hegab, Catherine Hobday, Veena Rao, Jyotsna Shrimankar, Mohamed Tabaqchali, David Wilson, Oliver Jones, Neil Mortensen, Andrew Slater, Aron Szuts, Lai Wang, Bryan Warren, Andrew Weaver, Mukhtar Ahmad, Julian Alexander, Maxine Flubacher, David Tarver, Suhail Baluch, Richard Beable, David Cowlishaw, Antony Higginson, Prokopios Vogiatzis, Neil Cruickshank, Howard Joy, David Peake, Ulises Zanetto, Mark Saunders, Arthur Sun-Myint, Rajaram Sripadam, Rachel Cooper, Paul Hatfield, Mark Teo, Arthur Allan, Ian Geh, John Glaholm, Mark Goldstein, Rahul Hejmadi, Gerald Langman, Dion Morton, Cyril Nelson, Deborah Tattersall, Stephen Falk, Robert Longman, Huw Roach, Jamshed Shabbir, Golda Shelley-Fraser, Michael Thomas, Neil Cripps, Yasser Haba, Guy Harris, Max Hookway, Jay Simson, Angela Skull, Tijani Umar

**Affiliations:** a Leeds Institute for Medical Research, University of Leeds, Leeds, UK; b Division of Pathology and Data Analytics, School of Medicine, University of Leeds, Leeds, UK; c Cancer Research Clinical Trials Unit, University of Birmingham, Birmingham, UK; d Birmingham Clinical Trials Unit, University of Birmingham, Birmingham, UK; e Department of Colorectal Surgery, University Hospitals Birmingham NHS Foundation Trust, Birmingham, UK; f Department of Radiation Oncology, University Hospitals Birmingham NHS Foundation Trust, Birmingham, UK; g Department of Colorectal Surgery, Central Manchester University Hospitals NHS Foundation Trust, Manchester, UK; h Department of Colorectal Surgery, North Tees and Hartlepool NHS Foundation Trust, Durham, UK; i Department of Colorectal Surgery, Newcastle upon Tyne Hospitals NHS Foundation Trust, Newcastle upon Tyne, UK; j Department of Colorectal Surgery, East Suffolk and North Essex NHS Foundation Trust, Colchester, UK; k Department of Colorectal Surgery, Portsmouth Hospital NHS Trust, Portsmouth, UK; l Department of Colorectal Surgery, Bradford Teaching Hospitals NHS Foundation Trust, Bradford, UK; m Department of Colorectal Surgery, Oxford University Hospitals NHS Foundation Trust, Oxford, UK; n National Cancer Research Institute, London, UK

## Abstract

**Background:**

Older patients with early-stage rectal cancer are under-represented in clinical trials and, therefore, little high-quality data are available to guide treatment in this patient population. The TREC trial was a randomised, open-label feasibility study conducted at 21 centres across the UK that compared organ preservation through short-course radiotherapy (SCRT; 25 Gy in five fractions) plus transanal endoscopic microsurgery (TEM) with standard total mesorectal excision in adults with stage T1–2 rectal adenocarcinoma (maximum diameter ≤30 mm) and no lymph node involvement or metastasis. TREC incorporated a non-randomised registry offering organ preservation to patients who were considered unsuitable for total mesorectal excision by the local colorectal cancer multidisciplinary team. Organ preservation was achieved in 56 (92%) of 61 non-randomised registry patients with local recurrence-free survival of 91% (95% CI 84–99) at 3 years. Here, we report acute and long-term patient-reported outcomes from this non-randomised registry group.

**Methods:**

Patients considered by the local colorectal cancer multidisciplinary team to be at high risk of complications from total mesorectal excision on the basis of frailty, comorbidities, and older age were included in a non-randomised registry to receive organ-preserving treatment. These patients were invited to complete questionnaires on patient-reported outcomes (the European Organisation for Research and Treatment of Cancer Quality of Life [EORTC-QLQ] questionnaire core module [QLQ-C30] and colorectal cancer module [QLQ-CR29], the Colorectal Functional Outcome [COREFO] questionnaire, and EuroQol-5 Dimensions-3 Level [EQ-5D-3L]) at baseline and at months 3, 6, 12, 24, and 36 postoperatively. To aid interpretation, data from patients in the non-randomised registry were compared with data from those patients in the TREC trial who had been randomly assigned to organ-preserving therapy, and an additional reference cohort of aged-matched controls from the UK general population. This study is registered with the ISRCTN registry, ISRCTN14422743, and is closed.

**Findings:**

Between July 21, 2011, and July 15, 2015, 88 patients were enrolled onto the TREC study to undergo organ preservation, of whom 27 (31%) were randomly allocated to organ-preserving therapy and 61 (69%) were added to the non-randomised registry for organ-preserving therapy. Non-randomised patients were older than randomised patients (median age 74 years [IQR 67–80] *vs* 65 years [61–71]). Organ-preserving treatment was well tolerated among patients in the non-randomised registry, with mild worsening of fatigue; quality of life; physical, social, and role functioning; and bowel function 3 months postoperatively compared with baseline values. By 6–12 months, most scores had returned to baseline values, and were indistinguishable from data from the reference cohort. Only mild symptoms of faecal incontinence and urgency, equivalent to less than one episode per week, persisted at 36 months among patients in both groups.

**Interpretation:**

The SCRT and TEM organ-preservation approach was well tolerated in older and frailer patients, showed good rates of organ preservation, and was associated with low rates of acute and long-term toxicity, with minimal effects on quality of life and functional status. Our findings support the adoption of this approach for patients considered to be at high risk from radical surgery.

**Funding:**

Cancer Research UK.

**Research in context**

**Evidence before this study**

The European Organisation for Research and Treatment of Cancer calculated that cancer incidence is 11 times higher in older people (aged ≥65 years) than in those younger than 65 years. Although rectal cancer has a peak incidence at age 80 years, the average age of participants in rectal cancer clinical trials is generally younger than 65 years. Two systematic reviews into the outcomes of older patients with rectal cancer reported on the paucity of evidence to guide treatment as a result of under-representation in clinical trials. These reviews, along with consensus guidelines published in 2021, point to the need to consider modifying the use of radical surgery in patients with frailty, comorbidity, or both, to reduce the associated risk of morbidity and mortality. Standard surgical treatment according to the principles of total mesorectal excision benefits younger patients the most, whereas older patients are not only susceptible to higher complication rates but also to more marked consequences of these complications, leading to an increased risk of mortality in the year following surgery. Following curative surgery for rectal cancer, postoperative mortality at 6 months is around 5% among patients aged 65–74 years, 14% among those aged 75–84 years, and 29% among those aged 85–95 years. Few studies have prospectively reported patient-reported outcomes or toxicity rates in the longer term—including health-related quality of life in both the acute and long-term setting—and few studies have reported to the quality detailed in Consolidated Standards of Reporting Trials (CONSORT) guidelines for reporting patient-reported outcomes, limiting interpretation and clinical relevance.

Organ preservation is an alternative approach to the management of early-stage rectal cancer in patients who are at increased risk from radical surgery. Conventionally fractionated chemoradiotherapy with or without selective transanal local excision offers organ-preservation rates of 64–91%, with isolated local relapse of 5% or less. However, problematic toxicities with concurrent chemotherapy render this approach unsuitable for many older or frailer patients.

The TREC study compared use of conventional radical surgery versus organ preservation via hypofractionated SCRT (25 Gy in five fractions) and transanal endoscopic microsurgery (TEM), and found that the organ-preservation approach was acceptable for patients with early-stage rectal cancer. Compared with radical surgery, treatment by organ preservation was associated with fewer serious complications, reduced acute patient-reported toxicity, and had small effects on health-related quality of life and functional status among patients with a median age of 65 years. The TREC study also incorporated a non-randomised registry offering organ-preserving treatment via SCRT and TEM to older patients (median age 74 years)considered to be at high risk from conventional radical surgery. In this non-randomised registry cohort, organ preservation was achieved in 56 (92%) of 61 patients and local recurrence-free survival was estimated to occur in 91% at 3 years.

**Added value of this study**

To our knowledge, this current study is the first to report data on patient-reported outcomes from patients treated with an organ-preservation approach who were considered to be at high risk of complications from standard surgery. These data show that the approach of SCRT followed by TEM has minimal effect on patients' quality of life and patient-reported bowel function following treatment and over 36 months of follow-up.

Endpoints used to assess outcomes in oncology trials often fail to address concerns of older patient populations; notably few published studies in older patients with rectal cancer have incorporated patient-reported outcomes and assessment of health-related quality of life. In the organ-preservation setting, very few studies have prospectively reported patient-reported outcomes or longer-term toxicity rates, including health-related quality of life both in the acute and long-term setting.

This analysis provides high-quality data on patient-reported outcomes, reported to CONSORT standards, on the experience of older and frailer patients treated with an organ-preservation approach. We present their symptom trajectory over time compared with the younger randomised cohort in the TREC study and with an additional cohort of age-matched individuals from the UK general population with no cancer diagnosis to aid interpretation. Alongside excellent rates of organ preservation and low local recurrence rates observed in this non-randomised registry, these findings support use of organ preservation through SCRT and TEM as a leading option for patients with early-stage rectal cancer who are considered to be unfit for radical surgery, aiding clinical decision making.

**Implications of all the available evidence**

Both patient groups and health-care professionals recognise the potential benefits of an organ-preservation approach to reduce the acute and long-term side-effects associated with radical surgery, and avoid stoma placement in patients with low rectal cancer. However, there is limited evidence to support this approach in an older and frailer population, in part due to the paucity of elderly patients included in clinical trials. Surgical treatment is challenging in older patients due to decreasing performance status and the increased burden of comorbidities associated with an increased risk of developing complications postoperatively. Surgical resection can be combined with a permanent stoma in older patients to reduce the risk of anastomotic complications; however, the ability to manage a stoma is compromised if either dexterity or eyesight is impaired. Risk of postoperative complications has been further heightened by COVID-19, which disproportionately affects frail patients; therefore, an organ-preservation approach that avoids radical surgery could be beneficial for this population.

The feasibility of achieving primary organ preservation via conventionally fractionated chemoradiotherapy is not in doubt; however, concerns relating to acute toxicity are a barrier to implementation in older populations where frailty and multiple morbidity are prevalent. Hypofractionated SCRT is tolerated better in older patients, and this approach has gained in popularity during the COVID-19 pandemic as the number of hospital visits needed to complete treatment is reduced and the risk of immunosuppression is avoided.

Patient-reported outcome data from the TREC trial indicate that SCRT and TEM has minimal impact upon quality of life and bowel function in older patients considered at high risk for conventional radical surgery. Alongside excellent rates of organ preservation and relatively low risk of relapse, these findings support use of organ preservation via SCRT and TEM as a leading option for patients with early rectal cancer who are unfit for radical surgery. It might be possible to improve organ preserving therapy further through radiotherapy fields of smaller volumes, risks adapted for early tumours, and the introduction of non-operative management following complete response. These refinements are currently being evaluated in the STAR-TREC study (NCT02945566).

## Introduction

Radical resection adhering to the principles of total mesorectal excision is considered to be the standard of care for patients with early-stage rectal cancer.^1^ As the incidence of rectal cancer rises exponentially with age and as the older population continues to grow, it is anticipated that the number of older patients with rectal cancer will steadily increase. Available evidence suggests that standard surgical treatment most benefits younger patients, whereas older patients have higher rates of postoperative complications and 6-month and 12-month mortality.^2^, ^3^ Although data from the Dutch registry show promising trends in improved postsurgical in-hospital or 30-day mortality for older patients, it remains unknown whether this translates to longer-term improvements.^4^ This increased risk of postoperative mortality among older patients potentially overshadows the beneficial treatment effect of surgery. However, little high-quality prospective data exist to guide treatment choices for older patients diagnosed with rectal cancer. Although rectal cancer trials do not generally set upper age limits for participants, it is notable that older patients are under-represented in study populations.^2^, ^5^

Older patients with early-stage rectal cancer might legitimately consider trading the upfront risks of major surgical resection for notionally safer, organ-preserving alternatives.^6^ Several studies including CARTS, ACOSOG Z6041, and GRECCAR 2 have evaluated organ-preservation via conventionally fractionated chemoradiotherapy combined with transanal excision in relatively young, healthy populations of patients. The average age of participants in these studies was 61–65 years, well below the peak incidence at 80 years.^7^, ^8^, ^9^ Although organ-preservation rates of 64–91% were encouraging and the risk of isolated local relapse low, these studies highlighted relatively poor safety and tolerability and were associated with cumulative toxicities of multiple treatment modalities (in particular from patients who required total mesorectal excision)—issues that are likely to be exacerbated in older patients.^2^, ^8^ Alongside, the immunosuppressive risks of concurrent chemotherapy, conventionally fractionated radiotherapy also requires at least 26 hospital attendances, a particular concern for cancer patients aged 70 years or older who wish to minimise the risk of hospital exposure to COVID-19.^10^

The TREC trial randomly assigned patients with early-stage rectal cancer (median age 65 years [IQR 60–74]) to either radical surgery according to the principles of total mesorectal excision in accordance with National Institute for Health and Care Excellence (NICE) guidance or to a novel organ-preservation strategy—ie, short-course radiotherapy (SCRT) followed by transanal endoscopic microsurgery (TEM) 8–10 weeks later; the primary aim was to assess the feasibility of this approach.^6^ Unlike conventionally fractionated chemoradiotherapy, hypofractionated radiotherapy requires only five hospital treatment attendances. Supported by a strong evidence base, use of this technique has increased substantially during the COVID-19 pandemic.^7^ The study found that, compared with standard radical surgery, organ preservation was associated with fewer serious complications, reduced acute and long-term patient-reported toxicity, and had little effect on health-related quality of life and function during the 3-year follow-up.

Importantly, TREC also incorporated a non-randomised registry to capture the outcomes of patients who were offered organ preservation without randomisation because they were considered to be unsuitable for major surgery or at high risk of complications by the colorectal cancer multidisciplinary team. This non-randomised registry comprised 61 patients (median age 74 years [IQR 67–80]), who were treated with the organ-preservation strategy.^6^ Organ preservation was achieved in 56 (92%) of 61 patients, with a 9% risk of isolated pelvic recurrence at 3 years. Although the trial found encouraging findings on the safety, tolerability, and efficacy of organ preservation through the use of SCRT and TEM for the treatment of older patients, the effect of this organ-preservation therapy on patient functional outcomes and quality of life has not yet been defined. We therefore aimed to investigate to what extent organ-preserving therapy through SCRT and TEM preserved function and quality of life in older patients with early-stage rectal cancer.

## Methods

### Study design and participants

TREC was a randomised, open-label feasibility study conducted at 21 tertiary referral centres specialising in the treatment of early-stage rectal cancer across the UK.^6^ Eligible patients were adults (aged ≥18 years) with stage T1–2 rectal adenocarcinoma (maximum diameter ≤30 mm) and no lymph node involvement or metastasis. Patients with a history of previous pelvic radiotherapy were excluded.

Eligible patients considered to be suitable for the study were invited to enrol by the local colorectal multidisciplinary team. Patients were randomly assigned (1:1) to either total mesorectal excision without pre-operative radiotherapy, in accordance with NICE guidance, or to organ preservation through hypofractionated SCRT followed by TEM 8–10 weeks later.^6^ The sample size was set to enable exploratory evaluation of histopathological downstaging of high-risk features within the randomised feasibility trial. Full details on study design are reported elsewhere.^6^

A non-randomised registry was also included to capture treatment outcomes in patients for whom randomisation was considered inappropriate by the multidisciplinary team. Randomisation was considered inappropriate for patients for whom there was either a strong clinical indication for one of the treatment options (following local management strategies), or, in particular, for patients who were referred for consideration of organ-preserving therapy based upon their frailty status, presence of comorbidities, or older age. Where the multidisciplinary team strongly preferred one of the two treatment groups, patients were invited to participate in the non-randomised registry and were entered by internet or telephone registration prior to treatment. No recruitment target was set for the non-randomised registry. This paper reports on non-randomised patients who received an organ-preservation approach.

Participants provided written informed consent before entry onto the registry. Ethical approval was granted by West Midlands, Black Country Research Ethics Committee (10/H1202/81).

### Procedures

Baseline investigations and procedures have been described in detail previously.^6^ The organ-preservation approach consisted of three-dimensional conformal radiotherapy (25 Gy in five fractions), followed by transanal microsurgery after 8–10 weeks. Histopathological evaluation of surgical specimens established the presence of features indicating a high risk of relapse following the therapy (ie, high-risk features).^8^ Patients with TEM specimens showing high-risk features were offered conversion from the organ-preservation approach to total mesorectal excision.

All participants were invited to complete the following validated questionnaires: the European Organisation for Research and Treatment of Cancer Quality of Life (EORTC-QLQ) questionnaire core module (QLQ-C30) and colorectal cancer module (QLQ-CR29), the Colorectal Functional Outcome (COREFO) questionnaire, and EuroQol-5 Dimensions-3 Level (EQ-5D-3L).^9^, ^10^, ^11^, ^12^ QLQ-C30 measures overall quality of life (global health status); physical, role, social, emotional, and cognitive functioning; and common symptoms affecting patients with cancer. QLQ-CR29 addresses disease-specific concerns, including bowel (and stoma), urinary and sexual symptoms, body image, and health anxiety. EORTC-QLQ patient-reported outcome data for the randomised patients were reported with the main trial outcomes.^6^ The COREFO faecal incontinence grading system comprises 27 questions with five subscales relating to bowel function: incontinence (ie, flatulence, solid, and liquid stools), social impact (ie, toilet dependence and impact on activities), frequency (ie, day and night), stool-related aspects (ie, pain, skin soreness, and blood loss), and need for medication. A total score is derived by excluding an item referring to constipation—ie, “Have you used medicines to make your stools thinner?” EQ-5D-3L comprises five dimensions (ie, mobility, self-care, usual activities, pain or discomfort, and anxiety or depression) and a visual analogue scale to rate health.

Questionnaires were completed at baseline (before start of treatment but after registration), and subsequently at months 3, 6, 12, 24, and 36. The 3-month timepoint represented completion of all organ-preserving treatment and the remainder of the timepoints represented the follow-up period. Questionnaires were completed by patients on paper at the time of clinic appointments or by post.

### Statistical analysis

Randomised and non-randomised patient characteristics were compared using either a t test or a Wilcoxon test for continuous variables (dependent on normality of data), or a χ^2^ test for categorical variables. A Bonferroni adjustment was made to maintain the overall type I error of the comparison of variables at 5%.

Completion and scale compliance of patient-reported outcomes were established and questionnaire guidelines for the management of missing data were followed, deriving missing values in scaled responses by imputation.^12^, ^13^ All EORTC-QLQ item responses were converted from a four-point Likert-type scale using linear transformation onto a 0–100 scale. When interpreting responses, higher symptom scores reflected a greater severity of symptoms, whereas higher functional scores reflected a better level of functioning.^13^ Differences in mean scores were classified as either a small change of 5–10 points (unlikely to be clinically relevant), a moderate change of up to 20 points, or a large change of more than 20 points.^14^ Responses to the three-point ordinal descriptive items of the EQ-5D-3L were translated into a single summary index ranging from –0·59 to 1·00 using a value set relevant to the UK. Responses for the COREFO items are on a five-point Likert-type scale (except for two items on bowel frequency) and all were converted using a linear transformation onto a 0–100 scale. Higher scores indicated worse bowel function; minimally important differences are not available.^15^

Key consensus outcomes derived from the core outcome set for colorectal cancer surgery trials, developed by patients and health-care professionals, and suitable for reporting patient-reported outcomes, were selected a priori to report graphically.^16^ These consensus patient-reported outcomes items included quality of life (global health status), physical functioning, sexual function (ie, libido, impotence, and dyspareunia), faecal incontinence, diarrhoea, and faecal urgency. In addition, we reported functioning issues considered to be important to older people, in particular social functioning (interference with family life or social activities), role functioning (ability to carry out normal daily activities and hobbies), and two additional symptom items—ie, “Did you have pain in your buttocks, anal area, or rectum?” and “Have you had blood in your stools?”—given the potential toxicity related to organ-preserving approaches.^17^

To identify factors that were important in the prediction of poor patient-reported outcomes and to inform decision making around organ preservation, we first analysed pooled patient-reported outcomes data from both those patients who were randomly allocated to receive the organ-preservation approach and those who received organ preservation as a result of entry onto the non-randomised registry. Factors evaluated at baseline were mechanism of entry into the trial (ie, non-randomised or randomised), age, sex, tumour height from anal verge (mm), and T stage (stratification factor within the trial). Regression models were fitted using RStan and the brms package with cumulative family to estimate the response as either an ordinal value (for QLQ-C30 items) or continuous value (for EQ-5D-3L index values).^18^, ^19^, ^20^, ^21^ The Bayesian models account for the fact that repeated observations from the same patients are likely to be correlated. In this analysis, minimally informative prior beliefs regarding how quality-of-life outcomes change over time were combined with the observed data collected as part of the trial to ascertain posterior estimates, from which the probability that patients had a superior outcome on one treatment over the other were ascertained. Cumulative models were used for QLQ-C30 due to the ordinal scoring for many items, and a sensitivity analysis was performed using continuous models for scaled items (only negligible differences were found). EQ-5D-3L index values were assumed to be continuous. Population-level terms were used to estimate the average response value, an adjustment for each timepoint, and an interaction between timepoint and mechanism of entry to the trial. Patient-level intercepts were included to address serial correlation in responses. All models were fit without divergences. No other diagnostics showed problems in model fitting. Posterior predictive checks showed that the models fit the observed data well and all assumptions were met.

To aid interpretation, data from patients in the non-randomised registry are presented alongside reference data from age-matched individuals from the UK general population where available, and are compared with data from patients who were randomly assigned to the organ-preservation arm in the TREC study.^22^, ^23^ Additionally, comparison with the UK age-matched data was sought through ascertainment of the posterior probability that the fitted model predicted individuals on the trial (both randomised and in the non-randomised registry) who had an inferior outcome.

All statistical analysis was performed using R (version 4.1.0). This study is registered with the ISRCTN registry, ISRCTN14422743.

### Role of the funding source

The funder of the study had no role in study design, data collection, data analysis, data interpretation, or writing of the report.

## Results

**Figure 1:**
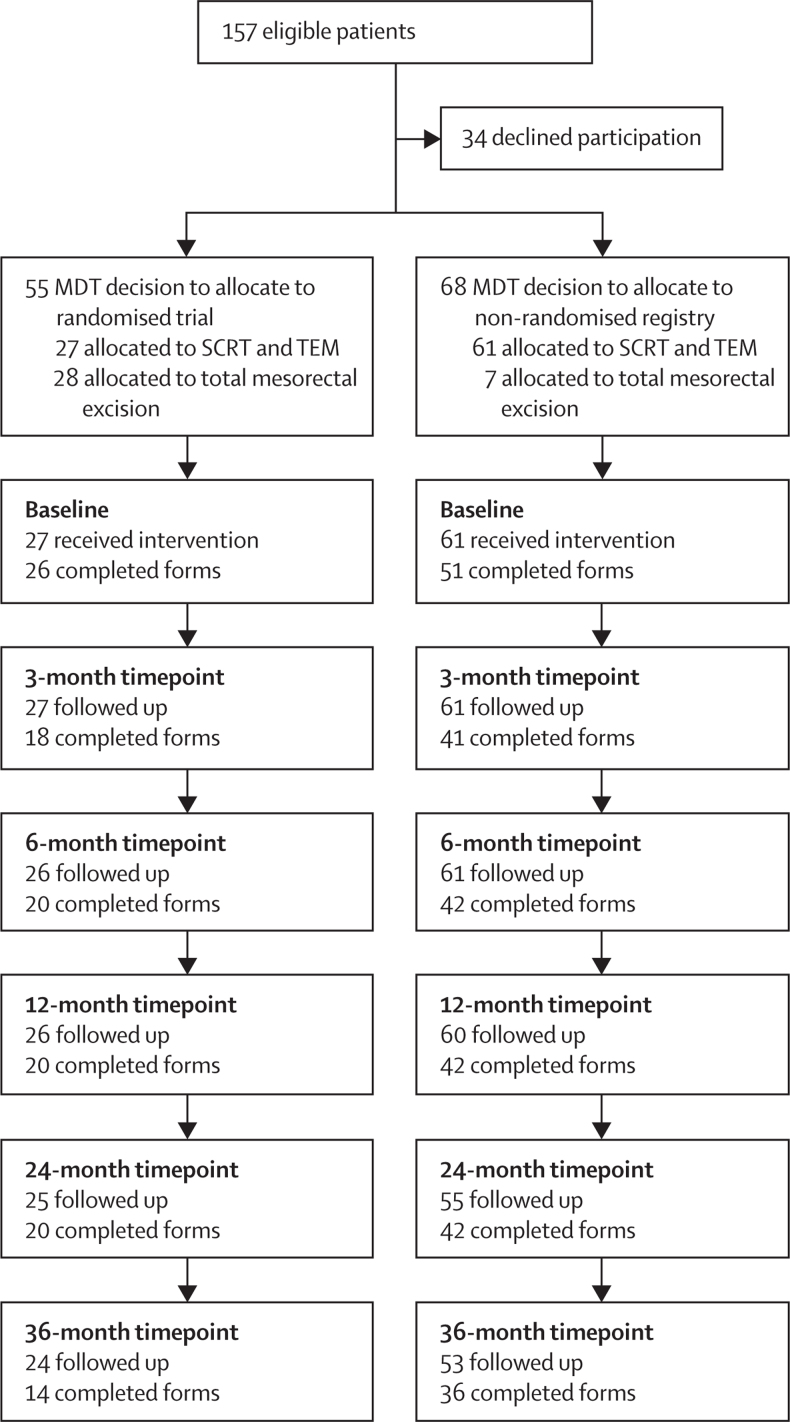
Study profile and patient-reported outcome form completion rates over the study period Surveys were considered to be completed if at least one patient-reported outcome was reported. MDT=multidisciplinary team. SCRT=short-course radiotherapy. TEM=transanal endoscopic microsurgery.

Between July 21, 2011, and July 15, 2015, 88 patients with early-stage rectal cancer were enrolled onto the TREC study to receive organ preservation.^6^ 27 (31%) patients were randomly assigned to have organ preservation and 61 (69%) patients were allocated to the non-randomised registry (figure 1). Patient characteristics, pre-operative staging information, and treatment outcomes—including rates of organ preservation, compliance, stoma, second surgery, complications, patterns of recurrence, overall survival, and disease-free survival—are reported in detail elsewhere.^6^ Compared with patients randomly assigned to organ preservation, those in the non-randomised registry were older (median age 74 years [IQR 67–80] *vs* 65 years [61–71]; p=0·00053), with higher (although not significant) overall Charlson comorbidity index scores and American Society of Anesthesiologists grades (table 1). Only four (7%) of 61 patients in the non-randomised registry had planned early conversion to radical surgery, despite 24 (39%) patients being considered to have high-risk histological features following transanal endoscopic microsurgery with or without total mesorectal excision, and three (5%) patients required permanent stomas following abdominoperineal excision. For five (56%) of the nine patients who died during follow-up for whom data are available, colorectal cancer was not reported to be the cause of death.

**Table 1:** Participant characteristics

	**Non-randomised patients (n=61)**	**Randomised patients (n=27)**	EORTC cohort*(n=540)
**Age, years**			
Mean (SD)	73·07 (8·87)	65·74 (7·22)	65·54 (7·48)
Median (IQR)	74 (67–80)	65 (61–71)	65 (59–72)
**Sex**			
Female	22 (36%)	8 (30%)	269 (50%)
Male	39 (64%)	19 (70%)	271 (50%)
**Charlson Comorbidity Index**			
0	16 (26%)	14 (52%)	..
1	6 (10%)	4 (15%)	..
2	19 (31%)	3 (11%)	..
3	4 (7%)	2 (7%)	..
4	1 (2%)	0	..
5	3 (5%)	1 (4%)	..
6	3 (5%)	0	..
7	0	1 (4%)	..
Missing data	9 (15%)	2 (7%)	..
**American Society of Anesthesiologists grade**			
1	24 (39%)	11 (41%)	..
2	23 (38%)	14 (52%)	..
3	13 (21%)	2 (7%)	..
Missing data	1 (2%)	0	..
**Any comorbidities**			
No	..	..	345 (34%)
Yes	..	..	589 (59%)
Prefer not to answer	..	..	70 (7%)
Missing data	..	..	2 (<1%)

Data are n (%) unless otherwise indicated. EORTC=European Organisation for Research and Treatment of Cancer.

* Data from a cohort of age-matched individuals without a cancer diagnosis from the UK general population.

Patient-reported outcome questionnaire completion rates are reported in figure 1. Among patients in the non-randomised registry, compliance to patient-reported outcome surveys was good, with a response rate of 65% (50 of 77) at 36 months (figure 1). Only four (7%) patients did not contribute any patient-reported outcome data to the study. Overall, questionnaire response rates and missing items did not differ significantly between the non-randomised and randomised patients. Other than items on female sexual function, no single item was under-reported.

To evaluate factors predictive of poor patient-reported outcomes, all organ-preservation data (from both the non-randomised and randomised patients) were initially pooled. No single factor, including mechanism of entry into the trial, age, sex, tumour height, or T stage, was found to be more important than another at predicting poor patient-reported outcomes (appendix pp 12–44). Therefore, subsequent analyses only adjusted for mechanism of trial entry to account for the clinical evaluation provided by the colorectal cancer multidisciplinary team to reflect that patients in the non-randomised registry were considered to be at higher risk from radical surgery than were those who were randomly assigned to organ-preservation therapy.

**Figure 2:**
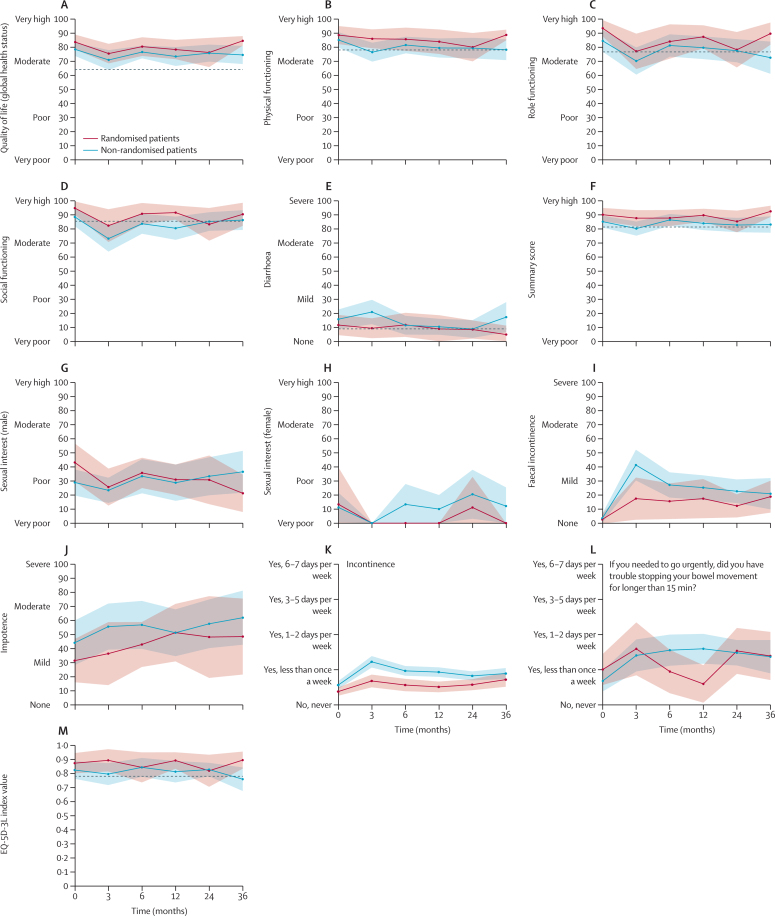
Core patient-reported outcomes Outcomes include EORTC-QLQ-C30 core items (A–E), EORTC QLQ C30 summary score (F), EORTC-QLQ-CR29 core items (G–J), Colorectal Functional Outcome scores (K, L), and EQ-5D-3L index value (M). Data are mean values and shaded areas are 95% CIs. Dashed line represents age-matched UK norm data from EORTC cohort C30 items (A–F) or EQ-5D index value (M). EORTC=European Organisation for Research and Treatment of Cancer. QLQ=quality of life. C30=core module. CR29=colorectal cancer module. EQ-5D-3L=EuroQol-5 Dimensions-3 Level.

Core patient-reported outcome items, plus rectal pain and bleeding, and role and social function, are presented in table 2 and figure 2, which show data for non-randomised and randomised patients with age-matched UK general population norm data (where available).^16^ A full summary of all remaining patient-reported outcome data can be found in the appendix (pp 2–8).

**Table 2:** Patient-reported outcome scores for core items following organ preservation and UK age-matched general population data

		EORTC cohort*	**Baseline**	**3 months**	**6 months**	**12 months**	**24 months**	**36 months**
**EORTC-QLQ-C30 core item scores**								
Quality of life (global health status)†								
	EORTC cohort*	64·2 (1·0); 540 (100%)	..	..	..	..	..	..
	Randomised patients	..	83·7 (13·6);26 (96%)	75·5 (15·0);18 (67%)	80·4 (15·1);20 (74%)	78·3 (15·6);20 (74%)	76·2 (23·6);20 (74%)	84·5 (6·4);14 (52%)
	Non-randomised patients	..	78·4 (17·1);51 (84%)	70·9 (22·9);41 (67%)	76·6 (14·8);42 (69%)	73·4 (22·2);42 (69%)	75·8 (20·0);42 (69%)	74·5 (18·9);33 (54%)
Physical functioning†								
	EORTC cohort	78·0 (1·0); 540 (100%)	..	..	..	..	..	..
	Randomised patients	..	88·6 (16·7);26 (96%)	86·0 (14·9);19 (70%)	85·7 (18·8);20 (74%)	83·9 (19·8);20 (74%)	80·0 (23·0);20 (74%)	88·7 (6·9);13 (48%)
	Non-randomised patients	..	85·1 (20·2);53 (87%)	76·5 (21·6);40 (66%)	81·5 (19·5);42 (69%)	79·4 (22·5);42 (69%)	79·4 (23·3);39 (64%)	78·2 (22·5);36 (59%)
Role functioning†								
	EORTC cohort	76·8 (1·2); 540 (100%)	..	..	..	..	..	..
	Randomised patients	..	93·6 (15·0);26 (96%)	77·2 (27·9);19 (70%)	84·2 (27·8);20 (74%)	87·5 (18·6);20 (74%)	78·3 (28·7);20 (74%)	89·7 (14·5);13 (48%)
	Non-randomised patients	..	84·6 (28·5);53 (87%)	70·3 (31·5);41 (67%)	81·3 (26·1);42 (69%)	79·8 (26·7);42 (69%)	77·6 (26·8);41 (67%)	72·7 (35·0);36 (59%)
Social functioning†								
	EORTC cohort	85·5 (1·1); 540 (100%)	..	..	..	..	..	..
	Randomised patients	..	94·9 (12·3);26 (96%)	82·4 (25·2);18 (67%)	90·8 (17·5);20 (74%)	91·7 (11·5);20 (74%)	83·3 (26·5);20 (74%)	90·5 (15·6);14 (52%)
	Non-randomised patients	..	88·6 (23·7);51 (84%)	73·2 (30·2);41 (67%)	83·7 (23·4);42 (69%)	80·6 (27·3);42 (69%)	85·3 (21·8);42 (69%)	86·4 (20·6);33 (54%)
Diarrhoea								
	EORTC cohort	8·8 (0·9); 540 (100%)	..	..	..	..	..	..
	Randomised patients	..	11·5 (18·7);26 (96%)	9·3 (15·4);18 (67%)	11·7 (19·6);20 (74%)	8·8 (21·8);19 (70%)	8·3 (14·8);20 (74%)	4·8 (12·1);14 (52%)
	Non-randomised patients	..	15·7 (25·3);51 (84%)	20·8 (27·9);40 (66%)	11·4 (21·9);41 (67%)	10·3 (18·8);42 (67%)	8·7 (20·9);42 (69%)	17·2 (31·3);33 (54%)
**EQ-5D-3L scores**								
EQ-5D-3L index value		0·779‡	..	..	..	..	..	..
	Randomised patients	..	0·87 (0·19);26 (96%)	0·89 (0·17);18 (67%)	0·84 (0·24);19 (70%)	0·89 (0·13);20 (74%)	0·82 (0·26);20 (74%)	0·89 (0·12);14 (52%)
	Non-randomised patients	..	0·82 (0·23);52 (85%)	0·79 (0·25);41 (67%)	0·84 (0·22);42 (69%)	0·81 (0·25);40 (66%)	0·83 (0·16);42 (69%)	0·76 (0·26);36 (59%)
**EORTC-QLQ-C29 core item scores**								
Faecal incontinence§								
	Randomised patients	..	2·9 (9·6);23 (85%)	17·6 (31·4);17 (63%)	15·8 (28·0);19 (70%)	17·6 (29·1);17 (63%)	12·5 (16·7);16 (59%)	19·0 (21·5);14 (52%)
	Non-randomised patients	..	3·7 (10·6);45 (74%)	41·4 (33·7);37 (61%)	27·4 (28·5);39 (64%)	25·4 (27·3);38 (62%)	22·9 (25·3);35 (57%)	21·1 (30·9);30 (49%)
Stool frequency§								
	Randomised patients	..	9·4 (12·1);23 (85%)	26·5 (21·3);17 (63%)	18·4 (19·2);19 (70%)	18·5 (17·0);18 (67%)	14·6 (14·8);16 (59%)	14·3 (15·8);14 (52%)
	Non-randomised patients	..	13·7 (17·1);45 (74%)	27·6 (22·0);38 (62%)	22·2 (16·8);39 (64%)	21·9 (22·6);38 (62%)	18·5 (18·6);36 (59%)	21·4 (20·2);28 (46%)
Embarrassment about bowel function§								
	Randomised patients	..	5·8 (12·9);23 (85%)	17·6 (33·6);17 (63%)	12·3 (25·4);19 (70%)	16·7 (26·2);18 (67%)	10·4 (23·5);16 (59%)	7·1 (14·2);14 (52%)
	Non-randomised patients	..	2·2 (8·4);45 (74%)	30·6 (32·8);37 (61%)	23·1 (34·3);39 (64%)	22·8 (30·1);38 (62%)	25·0 (33·2);36 (59%)	27·2 (34·6);27 (44%)
Anal, rectal, or buttock pain								
	Randomised patients	..	3·8 (10·9);26 (96%)	14·0 (25·6);19 (70%)	10·0 (19·0);20 (74%)	5·0 (12·2);20 (74%)	13·3 (19·9);20 (74%)	7·1 (14·2);14 (52%)
	Non-randomised patients	..	8·2 (19·5);53 (87%)	17·5 (29·2);40 (66%)	11·1 (21·7);42 (69%)	7·9 (19·2);42 (69%)	6·5 (18·6);41 (67%)	10·5 (22·5);35 (57%)
Blood and mucus in stool								
	Randomised patients	..	16·7 (20·5);26 (96%)	8·8 (14·0);19 (70%)	6·7 (12·6);20 (74%)	5·0 (7·8);20 (74%)	5·8 (16·5);20 (74%)	2·4 (6·1);14 (52%)
	Non-randomised patients	..	21·7 (25·4);53 (87%)	17·9 (22·2);41 (67%)	11·5 (17·5);42 (69%)	4·8 (9·2);42 (69%)	5·3 (10·2);41 (67%)	11·0 (14·5);35 (57%)
Impotence								
	Randomised patients	..	31·4 (32·2);17 (63%)	36·4 (37·9);11 (41%)	42·9 (30·5;14 (52%)	51·3 (37·6);13 (48%)	48·1 (44·4);9 (33%)	48·5 (45·6);11 (41%)
	Non-randomised patients	..	44·0 (42·6);28 (46%)	55·6 (43·4);27 (44%)	56·8 (45·1);27 (44%)	51·2 (44·9);28 (46%)	57·6 (41·4);22 (36%)	61·9 (36·6);14 (23%)
Dyspareunia								
	Randomised patients	..	0·0 (0·0);4 (15%)	8·3 (16·7);4 (15%)	0·0 (0·0);2 (7%)	0·0 (0·0);2 (7%)	22·2 (38·5);3 (11%)	0·0 (0·0);2 (7%)
	Non-randomised patients	..	6·7 (21·1);10 (16%)	22·2 (38·5);3 (5%)	8·3 (15·4);8 (13%)	10·0 (31·6);10 (16%)	18·5 (33·8);9 (15%)	12·5 (35·4);8 (13%)
Body image issues†								
	Randomised patients	..	94·7 (10·2);25 (93%)	93·6 (10·0);19 (70%)	94·7 (8·7);20 (74%)	94·2 (8·6);19 (70%)	93·3 (9·8);20 (74%)	92·1 (10·2);14 (52%)
	Non-randomised patients	..	89·3 (16·5);52 (85%)	83·9 (22·8);40 (66%)	82·9 (13·9);42 (69%)	86·5 (18·3);42 (69%)	88·8 (17·2);41 (67%)	83·7 (29·8);35 (57%)
Sexual interest (male)†								
	Randomised patients	..	43·1 (28·3);17 (63%)	25·6 (24·2);13 (48%)	35·7 (20·5);14 (52%)	31·0 (20·5);14 (52%)	30·8 (31·8);13 (48%)	21·2 (22·5);11 (41%)
	Non-randomised patients	..	28·9 (25·9);30 (49%)	23·3 (25·0);30 (49%)	33·3 (32·7);28 (46%)	28·7 (35·3);29 (48%)	33·3 (35·8);27 (44%)	36·5 (34·8);21 (34%)
Sexual interest (female)†								
	Randomised patients	..	13·3 (29·8);5 (19%)	0·0 (0·0);5 (19%)	0·0 (0·0);4 (15%)	0·0 (0·0);2 (7%)	11·1 (19·2);3 (11%)	0·0 (0·0);2 (7%)
	Non-randomised patients	..	11·1 (20·6);15 (25%)	0·0 (0·0);6 (10%)	13·3 (23·3);10 (16%)	10·0 (16·1);10 (16%)	20·5 (32·0);13 (21%)	12·1 (22·5);11 (18%)
**Colorectal Functional Outcome scores**								
Incontinence§								
	Randomised patients	..	9·5 (21·5);24 (89%)	17·0 (29·1);18 (67%)	14·1 (27·5);17 (63%)	12·8 (24·5);19 (70%)	14·4 (26·1);20 (74%)	18·0 (28·4);14 (52%)
	Non-randomised patients	..	14·1 (27·9);47 (77%)	30·6 (37·7);38 (62%)	24·2 (33·9);42 (69%)	23·3 (32·5);40 (66%)	20·6 (31·0);42 (69%)	22·3 (34·0);34 (56%)
Social impact§								
	Randomised patients	..	13·1 (24·0);24 (89%)	24·0 (34·4);18 (67%)	18·8 (30·5);17 (63%)	14·6 (26·7);19 (70%)	22·1 (30·7);20 (74%)	26·2 (32·3);14 (52%)
	Non-randomised patients	..	15·6 (26·4);47 (77%)	31·0 (37·0);38 (62%)	27·2 (33·5);42 (69%)	24·1 (31·8);40 (66%)	21·7 (29·9);42 (69%)	23·0 (31·6);35 (57%)
Bowel frequency§								
	Randomised patients	..	10·4 (15·3);24 (89%)	18·1 (21·2);18 (67%)	13·9 (19·3);18 (67%)	16·2 (20·3);17 (63%)	18·1 (20·4);18 (67%)	14·4 (14·4);13 (48%)
	Non-randomised patients	..	12·5 (19·3);47 (77%)	20·7 (22·7);38 (62%)	15·8 (20·1);42 (69%)	17·4 (19·8);40 (66%)	15·2 (16·6);41 (69%)	16·1 (19·1);35 (57%)
Stool-related aspects§								
	Randomised patients	..	14·9 (26·2);24 (89%)	7·9 (21·1);18 (67%)	3·3 (14·7);18 (67%)	2·3 (12·0);19 (70%)	3·2 (11·8);19 (70%)	6·2 (18·6);14 (52%)
	Non-randomised patients	..	17·3 (28·4);47 (77%)	13·5 (25·9);38 (62%)	9·5 (17·8);41 (67%)	10·8 (22·2);40 (66%)	9·7 (22·5);42 (69%)	11·1 (25·1);35 (57%)
Need for medication§								
	Randomised patients	..	3·3 (15·4);23 (85%)	18·8 (32·0);18 (67%)	6·4 (21·1);17 (63%)	13·2 (25·9);19 (70%)	13·6 (29·5);19 (70%)	20·8 (35·3);14 (52%)
	Non-randomised patients	..	11·7 (25·7);45 (74%)	16·0 (30·8);36 (59%)	14·1 (29·8);41 (67%)	15·8 (31·6);40 (66%)	20·2 (32·9);40 (66%)	9·3 (25·4);33 (54%)
Total§								
	Randomised patients	..	10·7 (22·1);24 (89%)	18·6 (30·4);18 (67%)	13·5 (26·6);18 (67%)	12·5 (24·3);19 (70%)	15·9 (27·2);20 (74%)	19·6 (29·5);14 (52%)
	Non-randomised patients	..	14·6 (26·6);47 (77%)	26·3 (35·1);38 (62%)	21·7 (31·5);42 (69%)	20·8 (30·6);40 (66%)	19·2 (29·3);42 (69%)	19·2 (30·7);35 (57%)

Data are mean (SD); n (%), unless otherwise indicated. For scaled items, if at least half of the items from the scale were answered, the missing items were assumed to have values equal to the mean of those items, which were present for that respondent (EORTC QLQ-C30 scoring manual). EORTC=European Organisation for Research and Treatment of Cancer. QLQ=quality of life. C30=core module. EQ-5D-3L=EuroQol-5 Dimensions-3 Level. C29=colorectal cancer module.

* Data from a cohort of age-matched individuals without a cancer diagnosis from the UK general population.

† For function scores, higher scores indicated improved function (0–100); with symptoms, higher scores indicated worse symptoms (0–100).

‡ EQ-5D-3L data from an aged-matched patient population.

§ Scores for patients with and without a stoma combined.

Generally poorer quality-of-life (global health status) scores; summary scores; and physical, social, and role functioning scores were observed for the non-randomised patients compared with the randomised patients at baseline and during follow-up, supporting the multidisciplinary team's viewpoint that these patients were generally frailer at presentation (figure 2; table 2). Baseline scores among patients in the non-randomised registry were higher than among individuals in the reference UK age-matched EORTC cohort, which were derived from individuals without a cancer diagnosis. For patients in the non-randomised registry, global quality-of-life and physical functioning scores were worse at 3 months following treatment than at baseline, but recovered by 6 months to baseline values. There were no clinically relevant differences (>10 points) in quality-of-life or physical functioning scores between the non-randomised and randomised patients. Among patients in the non-randomised registry, quality-of-life scores did not fall below reference values. Social functioning scores for non-randomised patients decreased moderately from baseline and below the UK age-matched EORTC reference value at 3 months (as did scores for the randomised patients, but less markedly), but recovered by 6 months. A moderate deterioration from baseline in role functioning scores at 3 months persisted in non-randomised patients at 36 months; however, this was not substantially below data from the UK age-matched reference EORTC cohort. Importantly, a moderate reduction in rectal bleeding (blood and mucus in stool) was reported by 6 months and sustained over the remainder of the follow-up period, and no increase in anorectal pain (anal, rectal, or buttock pain) was observed in both groups (randomised and non-randomised).

For core bowel patient-reported outcome items, diarrhoea symptoms were mildly worse at baseline for patients in the non-randomised registry group than in the UK age-matched reference EORTC cohort. Additionally, at 3 months, mean scores for diarrhoea symptoms were moderately worse among patients in the non-randomised registry than among those randomly assigned to organ-preservation therapy or those in the UK age-matched EORTC cohort, although this difference resolved to baseline values from 6 months. Faecal incontinence scores (QLQ-CR29) deteriorated at 3 months and a moderate deterioration from baseline scores remained at the 36-month follow-up for both randomised and non-randomised patients; however, these mean scores were equivalent to reporting mild symptoms (figure 2I). The COREFO incontinence scale and COREFO faecal urgency item also showed a mild deterioration from baseline that was maintained up to 36 months in both randomised and non-randomised groups of patients, equivalent to reporting an episode of incontinence or bowel urgency less than once per week (figure 2K, L). In terms of sexual functioning in women, dyspareunia scores were difficult to interpret due to the low numbers of respondents in both the randomised and non-randomised groups; however, no marked deterioration in scores was reported (table 2). For women, sexual interest was low at baseline and throughout follow-up (figure 2H; table 2). Among men, impotence scores at most timepoints, including baseline, were around 10 points worse for patients in the non-randomised registry than for those randomly assigned to organ preserving therapy (figure 2J; table 2). Impotence scores deteriorated over the follow-up period and, at 36 months, there was a moderate deterioration in mean scores in both randomised and non-randomised groups of patients. Male sexual interest scores were similar in both groups (figure 2G; table 2). Patients in the non-randomised group had a mild improvement, whereas those in the randomised group had a large deterioration at 36 months.

For non-core bowel symptoms, assessed with QLQ-CR29 and COREFO, flatulence, stool frequency, embarrassment (about bowel function), toilet dependency, use of pads, and impact on social activities showed a mild to moderate deterioration in scores at 3 months compared with baseline for both randomised and non-randomised patients, which settled to baseline values or to mild or minimal symptoms by 6–12 months (table 2; appendix pp 2–8). The highest mean scores, equivalent to patient-reported mild to moderate symptoms, among both randomised and non-randomised patients at 36-month follow-up were for flatulence (QLQ-CR29) and incomplete bowel emptying (COREFO; appendix pp 4, 7). Urinary symptoms, including frequency and incontinence, remained broadly stable in both groups over the follow-up period.

Comparison of the EORTC-QLQ-C30 and EQ-5D scores found no notable difference between the randomised and non-randomised patients, or the different normal reference populations. The posterior probabilities of having a worse patient-reported outcome score than age-matched individuals from the UK general population were generally low given that patients in both groups tolerated the organ-preservation approach with little effect on patient-reported outcomes scores (table 3). In general, these posterior probabilities were slightly lower among patients in the non-randomised registry than among randomised patients, again reinforcing the multidisciplinary team's viewpoint that patients in the non-randomised registry were generally frailer. Apart from a deterioration in posterior probability scores at 3 months (eg, fatigue), which was probably attributable to treatment recovery, typically posterior probability scores showed a monotonically increasing trend from baseline to 36 months.

**Table 3:** Bayesian model-based analysis of deterioration over time following organ preservation compared with UK age-matched general population norm data

		**Baseline**	**3 months**	**6 months**	**12 months**	**24 months**	**36 months**
**EORTC-QLQ-C30 core outcomes**							
Quality of life (global health status)							
	Randomised patients	9·1%	20·0%	13·5%	15·4%	15·7%	15·0%
	Non-randomised patients	17·2%	26·0%	21·8%	23·4%	20·3%	24·1%
Physical functioning							
	Randomised patients	18·3%	27·1%	29·5%	28·9%	33·0%	30·7%
	Non-randomised patients	26·2%	40·4%	37·1%	37·4%	37·3%	41·4%
Role functioning							
	Randomised patients	14·6%	42·8%	34·5%	27·2%	39·8%	34·4%
	Non-randomised patients	28·0%	47·5%	37·4%	35·6%	39·2%	47·5%
Social functioning							
	Randomised patients	20·7%	46·9%	31·2%	33·4%	42·8%	40·8%
	Non-randomised patients	32·1%	58·9%	42·4%	52·1%	41·9%	40·1%
Diarrhoea							
	Randomised patients	28·1%	25·3%	32·1%	18·5%	23·0%	17·2%
	Non-randomised patients	33·2%	44·1%	27·1%	26·6%	22·6%	35·3%
**EORTC-QLQ-C30 non-core outcomes**							
Emotional functioning							
	Randomised patients	31·9%	20·6%	23·3%	23·2%	29·4%	25·9%
	Non-randomised patients	39·4%	37·5%	36·0%	34·5%	29·7%	30·6%
Cognitive functioning							
	Randomised patients	39·1%	39·3%	36·1%	41·8%	38·2%	29·7%
	Non-randomised patients	51·0%	53·2%	53·0%	52·8%	53·1%	60·5%
Fatigue							
	Randomised patients	24·9%	30·5%	33·8%	26·7%	31·4%	32·0%
	Non-randomised patients	31·5%	49·7%	40·3%	41·4%	39·4%	44·4%
Nausea and vomiting							
	Randomised patients	10·0%	6·1%	11·1%	21·0%	16·6%	10·0%
	Non-randomised patients	16·6%	18·8%	12·7%	13·2%	16·8%	14·4%
Pain							
	Randomised patients	21·8%	19·5%	34·3%	20·3%	26·4%	27·0%
	Non-randomised patients	20·4%	25·8%	24·5%	24·8%	23·9%	33·1%
Dyspnoea							
	Randomised patients	20·7%	16·5%	41·4%	39·5%	29·2%	33·0%
	Non-randomised patients	25·9%	34·8%	23·5%	29·1%	32·7%	39·4%
Insomnia							
	Randomised patients	44·3%	52·1%	53·4%	52·3%	40·8%	42·2%
	Non-randomised patients	54·8%	60·2%	55·2%	54·0%	60·4%	51·6%
Appetite loss							
	Randomised patients	14·7%	14·3%	16·8%	6·2%	23·6%	13·4%
	Non-randomised patients	18·5%	29·6%	20·8%	21·2%	22·2%	23·0%
Constipation							
	Randomised patients	0·4%	6·2%	10·4%	9·9%	23·2%	20·4%
	Non-randomised patients	34·0%	31·8%	39·5%	28·6%	41·4%	23·7%
Financial difficulties							
	Randomised patients	4·6%	6·8%	14·1%	6·9%	22·7%	0·6%
	Non-randomised patients	12·4%	19·5%	10·1%	16·6%	14·9%	8·4%
**EQ-5D-3L**							
Index value							
	Randomised patients	1·4%	0·7%	15·8%	1·3%	18·5%	7·7%
	Non-randomised patients	8·2%	30·4%	5·4%	17·5%	9·0%	81·4%

EORTC=European Organisation for Research and Treatment of Cancer. QLQ=quality of life. C30=core module. EQ-5D-3L=EuroQol-5 Dimensions-3 Level.

There was a notable increase in the posterior probability of having an inferior EQ5D index score compared with the UK age-matched population for those in the non-randomised cohort at 36 months (81·4%; table 3). However, the index estimate in the non-randomised cohort only differed by 0·019 (non-randomised 36 months: 0·76 [SD 0·04; n=36]; UK age-matched index value: 0·779) and a 0·1-point difference in index values is typically considered clinically meaningful.^24^

Finally, we evaluated the global quality-of-life and EQ5D scores from patients (both randomised and non-randomised) who converted from organ preservation to total mesorectal excision surgery (nine patients). There was no signal of worse overall scores than the general norm data at 36 months, although numbers were small (appendix pp 10–11).

## Discussion

While radical surgery currently remains the standard of care for treatment of patients with early-stage rectal cancer, there is a drive to look for safer treatment options due to high postoperative mortality rates in older and frailer patients.^2^, ^25^ Older people will often prioritise quality of life and functional status when balancing the potential benefits and harms of different cancer treatments; therefore, the goals of treatment for this patient group need to focus as much on quality of life and functional recovery as they do on conventional cancer outcomes.^25^ Unfortunately, quality of life and functional status are generally evaluated poorly or not at all in research studies.^17^ This problem is further compounded by under-representation of older patient populations in cancer trials.^5^ Our findings show that organ-preserving treatment by use of SCRT followed by TEM produced high organ-preservation rates of 92%, appeared to be safe, and was well tolerated in older patients, with the patient-reported benefits of organ preservation sustained over the 36-month follow-up.^6^ Patients who were considered to be unsuitable for standard radical resection of the rectum by the multidisciplinary team showed good recovery from organ-preserving treatment; had quality-of-life scores and physical and social functioning scores similar to those in their pre-treatment status; and their outcomes did not differ significantly from younger patients in the randomised cohort or to age-matched controls. Based on our previous work, we can assume that these results are markedly better than would have been achieved with a total mesorectal excision approach.^6^

Systematic reviews of the management of rectal cancer in older patients have highlighted the importance of adjusting management in those with frailty and comorbidities to reduce the risk of excessive mortality and morbidity associated with radical surgery.^26^, ^27^ However, due to the scarcity of high-quality clinical trials to guide the optimal approach to cancer management of these patients, there is wide variation in the delivery of care.^28^ An international population, cohort comparison across five countries found wide heterogeneity in 5-year survival, surgical rates, and radiotherapy rates among patients older than 80 years with stage I–III rectal cancer, with no clear pattern between treatment received and outcomes found.^28^ Following the extrapolation of data from clinical trials and prospective cohorts in younger populations,^6^, ^25^, ^29^, ^30^, ^31^, ^32^ consensus recommendations published in 2021 for the management of older patients with rectal cancer suggest local excision following a good response to neoadjuvant radiotherapy as a treatment option. The results of this current study provide much needed prospectively reported evidence to support this recommendation in older patients.

Similar efficacy between SCRT and chemoradiotherapy in the neoadjuvant setting, particularly in patients with intermediate risk rectal cancer, has long been recognised.^33^, ^34^ The Stockholm III trial of patients with stage I–IV rectal cancer showed reduced rates of postoperative morbidity in patients who received delayed surgery (4–8 weeks) following radiotherapy (SCRT or CRT), with no effect on cancer outcomes.^35^ The authors later concluded that SCRT and delayed surgery should be considered the recommended schedule in older or frailer patients, who might struggle to tolerate the additional toxicity of concomitant chemotherapy, in the setting of locally advanced rectal cancer.^36^ In the setting of early-stage rectal cancer, few studies have explored this novel organ-preservation approach of SCRT with or without local excision in older and frailer patients.^37^, ^38^, ^39^, ^40^ The largest of these studies, with TEM 8–10 weeks following SCRT, found this approach to be well tolerated, with no mortality associated, and that 48 (77%) of 62 patients were disease-free at a median follow-up of 13 months.^37^ Similar smaller cohorts found similar results.^38^, ^40^ Interestingly, in the non-randomised registry in the current study, only four patients underwent radical surgery after detection of high-risk features following TEM in 24 patients. These features were predefined on the basis of available evidence at the time of protocol development. However, only nine patients in the non-randomised registry had disease recurrence over the 5-year follow-up period: four with local recurrence only (two patients had salvage surgery and two patients were unfit for surgery), two with local and distant recurrences, and three with distant recurrences.^6^ This heterogeneous pattern of recurrence was also seen in the randomised cohorts and suggests our preconceived ideas of high risk features were not good at predicting recurrence in an early rectal cancer population. Future work exploring alternative biomarkers of recurrence, such as circulating tumour DNA, might help to stratify personalisation. However, for general adoption of an SCRT and TEM organ-preservation approach in a frailer population, it was essential to show the benefits to quality of life and functional status compared with standard of care. Through the use of validated patient-reported outcome measures, we are able to provide a unified, sensitive measure of how both the short-term and long-term toxicities related to radiotherapy and local surgery treatment affect patients and their lives.

Our study has several limitations. Although we used multicentre, prospective, registry data, the overall participant number was relatively small and the data were non-randomised. However, use of repeated measures over 36 months of follow-up, with validated patient-reported outcomes, enabled a robust comparison of quality of life, toxicity, and function, noting differences to data from age-matched individuals and from patients randomly assigned to organ-preservation treatment to guide interpretation, which provides an important contribution given that existing data on patient-reported outcomes in the setting of organ-preservation approaches are scarce.^41^ Although we found no specific complications related to the use of TEM within our study, the more selective use of TEM might be favourable given that a few studies have noted a potential increased risk of complications. One small study, in which TEM was carried out at 4–10 weeks (median 7 weeks) following SCRT, stopped recruitment early because two (14%) of 14 patients developed enterocutaneous fistulae.^39^ The earlier timing of TEM or the use of a wider local excision might have contributed to an increased rate of postoperative complications. A retrospective, single-centre case series reported painful suture dehiscence postoperatively following TEM, which might reflect the inclusion of only very low tumours (defined as tumours lying <5 cm from the anal verge) within the post-neoadjuvant chemoradiotherapy cohort in this series.^42^ Although an exploratory analysis of GRECCAR-2 reported lower rates of faecal incontinence in those patients who achieved organ preservation at 2 years, for patients requiring conversion to total mesorectal excision it also revealed higher rates of complications and an increased risk of abdominoperineal resection following TEM.^43^ Although, reassuringly, there was no signal of worse overall quality-of-life or EQ-5D-3L scores among patients who converted from organ preservation to total mesorectal excision surgery in the current study, the number of patients was small. Other approaches have been explored as an alternative to TEM, in particular endorectal brachytherapy boost following external beam (chemo)radiotherapy. In two studies, patient-reported outcomes showed favourable bowel function,^44^, ^45^ but high rates (34–78%) of rectal bleeding secondary to proctitis were also reported in an older cohort of patients.^44^, ^45^, ^46^ The interim results of the MORPHEUS study show more promising early results on toxicity and data on patient-reported outcomes have been collected.^47^ Compared with these results, the rates of blood and mucus in stool and rectal pain reported in the current study were extremely low. Further refinements to improve organ-preservation approaches in patients with early-stage rectal cancer are currently being evaluated in the STAR-TREC trial, including the role of smaller radiotherapy fields and selective use of TEM, which might further reduce toxicity.^48^

Our findings from this non-randomised registry show that the organ-preservation approach of SCRT and TEM delivers on the composite endpoint of good rates of organ preservation alongside good patient-reported toxicity, function, and quality of life in a cohort of frail patients with early-stage rectal cancer. These data support adoption of SCRT and TEM as a standard treatment for patients who are considered to be at high risk of complications from radical surgery.

**This online publication has been corrected. The corrected version first appeared at thelancet.com/healthy-longevity on November 23, 2022**

## Data sharing

Data sharing requests will be considered by the trial management group upon written request to trec@trials.bham.ac.uk. De-identified participant data or other prespecified data will be available, subject to a written proposal and an agreed data sharing agreement.

## Declaration of interests

AG is funded by a Cancer Research UK clinical trial fellowship (CRUK/28301). PQ and NPW were funded by a programme grant from Yorkshire Cancer Research. PQ is a National Institute for Health and Care Research senior investigator. NPW reports personal fees from Eisai. KB reports stock ownership in GlaxoSmithKline and UCB. All other authors declare no competing interests.
