# Supplementary material for: Quality-of-life outcomes in older patients with early-stage rectal cancer receiving organ-preserving treatment with hypofractionated short-course radiotherapy followed by transanal endoscopic microsurgery (TREC): non-randomised registry of patients unsuitable for total mesorectal excision
Source: Lancet Healthy Longev. 2022 Dec;3(12):e825–38. doi: 10.1016/S2666-7568(22)00239-2 (PMC9722406; doi:10.1016/S2666-7568(22)00239-2)
Supplement: Supplementary appendix [file mmc1.pdf]

THE LANCET
Healthy Longevity

Supplementary appendix

This appendix formed part of the original submission and has been peer reviewed. We post it as supplied by the authors.

Supplement to: Gilbert A, Homer V, Brock K, et al. Quality-of-life outcomes in older patients with early-stage rectal cancer receiving organ-preserving treatment with hypofractionated short-course radiotherapy followed by transanal endoscopic microsurgery (TREC): non-randomised registry of patients unsuitable for total mesorectal excision. *Lancet Healthy Longev* 2022; published online Nov 17. https://doi.org/10.1016/S2666-7568(22)00239-2.

Table of Contents for Supplementary Appendix

PRO mean scores for non-core items ***2***

Data on patients who converted from organ preservation to TME surgery ***10***

Review of Baseline Scores by Patient Characteristics ***12***

EORTC QLQ-C30 12
EORTC QLQ-CR29 17
COREFO Items 21
EQ5D Index Value 24

Review of Longitudinal Scores by Patient Characteristics ***30***

EORTC QLQ-C30 30
EORTC QLQ-CR29 34
COREFO 37
EQ-5D Index Value 40

List of Collaborators ***45***

PRO mean scores for non-core items

Supplementary Table 1: PRO mean scores for non-core items: EORTC QLQ-C30; EORTC QLQ-CR29 and COREFO mean score by question

	Treatment randomisation	Norm	Baseline	3 months	6 months	12 months	24 months	36 months
**EORTC QLQ-C30**		**Mean score (SE; N)**	**Mean score (SE; N)**	**Mean score (SE; N)**	**Mean score (SE; N)**	**Mean score (SE; N)**	**Mean score (SE; N)**	**Mean score (SE; N)**
**Non-Core Items**								
**Emotional Functioning §**	**EORTC Norm Cohort**	77.6 (1.0; 540)						
	**Randomised**		85.6 (2.5; 26)	90.3 (3.4; 18)	90.0 (2.6; 20)	88.8 (2.7; 20)	85.0 (4.2; 20)	90.5 (2.9; 14)
	**Non-randomised**		82.8 (2.4; 51)	82.0 (2.9; 41)	82.7 (2.9; 42)	82.9 (3.2; 42)	85.7 (2.8; 42)	83.1 (3.8; 33)
**Cognitive Functioning §**	**EORTC Norm Cohort**	86.2 (0.8; 540)						
	**Randomised**		89.7 (3.3; 26)	89.8 (4.7; 18)	91.7 (3.3; 20)	90.4 (2.6; 19)	87.5 (4.5; 20)	95.2 (3.7; 14)
	**Non-randomised**		86.3 (2.5; 51)	85.0 (2.7; 40)	86.5 (2.4; 42)	85.7 (2.5; 42)	86.1 (2.5; 42)	82.8 (2.9; 33)
**Fatigue**	**EORTC Norm Cohort**	30.3 (1.2; 540)						
	**Randomised**		16.2 (4.1; 26)	17.8 (4.7; 19)	18.9 (5.1; 20)	15.6 (3.7; 20)	21.1 (5.7; 20)	12.0 (3.9; 13)
	**Non-randomised**		18.4 (2.5; 53)	30.7 (3.9; 40)	22.5 (3.2; 42)	26.2 (4.2; 42)	25.3 (4.1; 40)	26.4 (3.7; 36)
**Nausea and Vomiting**	**EORTC Norm Cohort**	4.0 (0.5; 540)						
	**Randomised**		3.2 (2.6; 26)	1.8 (1.8; 19)	2.5 (1.8; 20)	5.8 (3.5; 20)	5.0 (3.0; 20)	1.3 (1.3; 13)
	**Non-randomised**		4.7 (1.7; 53)	5.0 (1.9; 40)	2.8 (1.4; 42)	2.8 (1.3; 42)	4.6 (2.1; 40)	2.8 (1.0; 36)
**Pain**	**EORTC Norm Cohort**	32.2 (1.4; 540)						

	**Randomised**		13.5 (3.9; 26)	9.6 (4.1; 19)	16.7 (5.8; 20)	10.5 (4.5; 19)	15.0 (3.6; 20)	9.5 (3.4; 14)
	**Non-randomised**		14.2 (3.5; 53)	16.7 (4.0; 41)	13.9 (3.3; 42)	15.1 (3.6; 42)	15.9 (4.1; 42)	21.3 (4.8; 36)
**Dyspnoea**	**EORTC Norm Cohort**	23.5 (1.3; 540)						
	**Randomised**		9.0 (3.9; 26)	7.0 (4.1; 19)	18.3 (7.0; 20)	18.3 (5.1; 20)	13.3 (6.1; 20)	7.7 (4.1; 13)
	**Non-randomised**		9.6 (2.6; 52)	13.0 (3.1; 41)	7.9 (2.7; 42)	12.2 (3.4; 41)	15.0 (3.9; 40)	19.4 (5.0; 36)
**Insomnia**	**EORTC Norm Cohort**	31.0 (1.5; 540)						
	**Randomised**		19.2 (4.2; 26)	24.6 (8.0; 19)	23.3 (4.9; 20)	23.3 (5.5; 20)	20.0 (6.6; 20)	10.3 (5.8; 13)
	**Non-randomised**		27.7 (3.7; 53)	33.3 (6.0; 40)	25.2 (4.3; 41)	27.0 (4.9; 42)	32.5 (5.3; 40)	26.9 (5.8; 36)
**Appetite Loss**	**EORTC Norm Cohort**	8.3 (0.9; 540)						
	**Randomised**		7.7 (4.6; 26)	5.3 (2.9; 19)	6.7 (3.9; 20)	5.0 (5.0; 20)	11.7 (4.4; 20)	2.6 (2.6; 13)
	**Non-randomised**		8.8 (2.7; 53)	13.7 (3.8; 39)	8.7 (3.8; 42)	8.7 (3.4; 42)	10.8 (3.7; 40)	11.1 (4.2; 36)
**Constipation**	**EORTC Norm Cohort**	8.1 (0.9; 540)						
	**Randomised**		0.0 (0.0; 26)	5.3 (5.3; 19)	3.3 (2.3; 20)	3.3 (2.3; 20)	10.0 (4.3; 20)	7.7 (5.5; 13)
	**Non-randomised**		15.7 (3.5; 53)	13.8 (3.3; 41)	16.7 (3.8; 42)	11.9 (3.2; 42)	17.5 (3.8; 40)	8.3 (2.8; 36)
**Financial Difficulties**	**EORTC Norm Cohort**	9.8 (1.0; 540)						
	**Randomised**		1.3 (1.3; 26)	1.9 (1.9; 18)	6.7 (5.2; 20)	1.7 (1.7; 20)	11.7 (6.1; 20)	0.0 (0.0; 14)
	**Non-randomised**		5.9 (2.4; 51)	8.3 (2.9; 40)	4.0 (2.0; 42)	8.7 (3.2; 42)	7.9 (3.0; 42)	3.1 (1.7; 32)
**§ FUNCTION scores: higher scores = improved function (0-100); SYMPTOMS: higher scores = worse symptoms (0-100)**								
**EORTC QLQ-C29**								
**Non-Core Items**								

**Faecal Incontinence (patients without a stoma)**	**Randomised**	2.9 (2.0; 23)	15.6 (7.9; 15)	11.1 (7.0; 15)	15.4 (8.1; 13)	11.9 (4.4; 14)	20.5 (6.0; 13)
	**Non-randomised**	3.7 (1.6; 45)	42.2 (6.0; 34)	27.8 (4.9; 36)	25.0 (4.7; 36)	21.2 (4.3; 33)	21.0 (5.9; 27)
**Faecal Incontinence (patients with a stoma)**	**Randomised**	n/a	33.3 (33.3; 2)	33.3 (13.6; 4)	25.0 (16.0; 4)	16.7 (16.7; 2)	0.0 (NA; 1)
	**Non-randomised**	n/a	33.3 (0.0; 3)	22.2 (11.1; 3)	33.3 (0.0; 2)	50.0 (16.7; 2)	22.2 (22.2; 3)
**Stool Frequency (patients without a stoma)**	**Randomised**	9.4 (2.5; 23)	24.4 (5.6; 15)	15.6 (4.4; 15)	19.0 (4.9; 14)	15.5 (4.1; 14)	14.1 (4.6; 13)
	**Non-randomised**	13.7 (2.6; 45)	28.1 (3.9; 35)	21.8 (2.9; 36)	21.8 (3.7; 36)	18.1 (3.1; 34)	21.3 (4.0; 25)
**Stool Frequency (patients with a stoma)**	**Randomised**	n/a	41.7 (8.3; 2)	29.2 (12.5; 4)	16.7 (6.8; 4)	8.3 (8.3; 2)	16.7 (NA; 1)
	**Non-randomised**		22.2 (5.6; 3)	27.8 (5.6; 3)	25.0 (25.0; 2)	25.0 (25.0; 2)	22.2 (14.7; 3)
**Embarrassment about bowel function^**	**Randomised**	5.8 (2.7; 23)	17.6 (8.1; 17)	12.3 (5.8; 19)	16.7 (6.2; 18)	10.4 (5.9; 16)	7.1 (3.8; 14)
	**Non-randomised**	2.2 (1.3; 45)	30.6 (5.4; 37)	23.1 (5.5; 39)	22.8 (4.9; 38)	25.0 (5.5; 36)	27.2 (6.7; 27)
**Embarrassment about bowels (patients without a stoma)**	**Randomised**	5.8 (2.7; 23)	17.8 (9.1; 15)	11.1 (7.0; 15)	19.0 (7.6; 14)	11.9 (6.6; 14)	7.7 (4.1; 13)
	**Non-randomised**	2.2 (1.3; 45)	31.4 (5.8; 34)	22.2 (5.8; 36)	22.2 (5.1; 36)	22.5 (5.4; 34)	25.0 (7.0; 24)
**Embarrassment about bowels (patients with a stoma)**	**Randomised**	n/a	16.7 (16.7; 2)	16.7 (9.6; 4)	8.3 (8.3; 4)	0.0 (0.0; 2)	0.0 (NA; 1)
	**Non-randomised**	n/a	22.2 (11.1; 3)	33.3 (19.2; 3)	33.3 (0.0; 2)	66.7 (33.3; 2)	44.4 (22.2; 3)
**Flatulence^**	**Randomised**	23.2 (4.9; 23)	29.2 (7.4; 16)	21.1 (6.4; 19)	29.6 (6.0; 18)	31.2 (6.4; 16)	33.3 (7.8; 14)
	**Non-randomised**	28.9 (3.8; 45)	48.2 (5.6; 38)	40.5 (4.3; 37)	39.8 (4.8; 36)	33.3 (5.3; 36)	39.1 (6.4; 29)
**Flatulence (patients without a stoma)**	**Randomised**	23.2 (4.9; 23)	23.8 (7.4; 14)	20.0 (7.1; 15)	28.6 (6.9; 14)	28.6 (6.9; 14)	35.9 (8.0; 13)
	**Non-randomised**	28.9 (3.8; 45)	48.6 (6.0; 35)	41.2 (4.7; 34)	40.2 (4.8; 34)	34.3 (5.5; 34)	39.7 (7.2; 26)
**Flatulence (patients with a stoma)**	**Randomised**	n/a	66.7 (0.0; 2)	25.0 (16.0; 4)	33.3 (13.6; 4)	50.0 (16.7; 2)	0.0 (NA; 1)
	**Non-randomised**	n/a	44.4 (11.1; 3)	33.3 (0.0; 3)	33.3 (33.3; 2)	16.7 (16.7; 2)	33.3 (0.0; 3)
**Sore Skin^**	**Randomised**	4.3 (3.2; 23)	19.6 (7.6; 17)	19.3 (6.9; 19)	7.4 (3.4; 18)	8.3 (4.8; 16)	7.1 (7.1; 14)
	**Non-randomised**	4.7 (1.8; 43)	23.1 (4.8; 36)	18.8 (4.0; 39)	16.7 (4.3; 38)	19.0 (4.8; 35)	19.5 (5.9; 29)
	**Randomised**	4.3 (3.2; 23)	17.8 (7.9; 15)	13.3 (7.8; 15)	4.8 (3.2; 14)	7.1 (5.2; 14)	7.7 (7.7; 13)

**Sore Skin (patients without a stoma)**	**Non-randomised**	4.7 (1.8; 43)	23.2 (5.1; 33)	18.5 (4.3; 36)	15.7 (4.5; 36)	19.2 (5.0; 33)	19.2 (6.5; 26)
**Sore Skin (patients with a stoma)**	**Randomised**	n/a	33.3 (33.3; 2)	41.7 (8.3; 4)	16.7 (9.6; 4)	16.7 (16.7; 2)	0.0 (NA; 1)
	**Registered**	n/a	22.2 (11.1; 3)	22.2 (11.1; 3)	33.3 (0.0; 2)	16.7 (16.7; 2)	22.2 (11.1; 3)
**Stoma Care Problems**	**Randomised**	n/a	16.7 (16.7; 2)	0.0 (0.0; 4)	0.0 (0.0; 4)	0.0 (0.0; 2)	0.0 (NA; 1)
	**Non-randomised**	n/a	11.1 (11.1; 3)	22.2 (11.1; 3)	33.3 (0.0; 2)	16.7 (16.7; 2)	33.3 (0.0; 3)
**Urinary Incontinence**	**Randomised**	3.8 (2.8; 26)	5.6 (3.0; 18)	5.0 (2.7; 20)	3.5 (2.4; 19)	14.0 (5.3; 19)	2.4 (2.4; 14)
	**Non-randomised**	8.2 (2.2; 53)	11.7 (3.5; 40)	7.9 (2.5; 42)	14.3 (3.4; 42)	11.4 (3.2; 41)	12.4 (3.4; 35)
**Dysuria**	**Randomised**	1.3 (1.3; 26)	0.0 (0.0; 18)	3.3 (2.3; 20)	3.3 (2.3; 20)	8.3 (4.1; 20)	2.4 (2.4; 14)
	**Non-randomised**	1.3 (0.9; 53)	5.8 (2.4; 40)	0.8 (0.8; 42)	0.0 (0.0; 42)	0.8 (0.8; 41)	1.0 (1.0; 35)
**Abdominal Pain**	**Randomised**	5.1 (3.6; 26)	3.5 (2.4; 19)	3.3 (2.3; 20)	5.0 (2.7; 20)	6.7 (3.1; 20)	4.8 (3.2; 14)
	**Non-randomised**	6.3 (2.5; 53)	15.4 (3.9; 41)	10.3 (3.1; 42)	4.0 (1.7; 42)	3.3 (1.6; 41)	8.8 (2.9; 34)
**Bloating**	**Randomised**	11.5 (3.7; 26)	8.8 (3.5; 19)	13.3 (3.7; 20)	11.7 (4.4; 20)	6.7 (3.9; 20)	7.7 (4.1; 13)
	**Non-randomised**	13.8 (3.0; 53)	22.5 (4.5; 40)	14.3 (3.4; 42)	14.3 (3.6; 42)	11.4 (2.5; 41)	15.7 (4.0; 34)
**Dry Mouth**	**Randomised**	11.5 (4.5; 26)	8.8 (4.3; 19)	13.3 (5.1; 20)	13.3 (4.5; 20)	20.0 (6.6; 20)	14.3 (4.6; 14)
	**Non-randomised**	18.2 (2.6; 53)	17.9 (3.3; 41)	16.7 (3.3; 42)	13.5 (2.8; 42)	17.1 (3.1; 41)	21.9 (3.3; 35)
**Hair Loss**	**Randomised**	1.3 (1.3; 25)	7.0 (3.2; 19)	3.3 (2.3; 20)	0.0 (0.0; 20)	1.7 (1.7; 20)	4.8 (4.8; 14)
	**Non-randomised**	0.7 (0.7; 50)	3.3 (2.0; 40)	7.1 (3.3; 42)	6.3 (3.1; 42)	4.1 (1.7; 41)	2.9 (1.6; 35)
**Taste Change**	**Randomised**	2.6 (1.8; 26)	0.0 (0.0; 19)	1.7 (1.7; 20)	3.3 (2.3; 20)	6.7 (3.9; 20)	2.4 (2.4; 14)
	**Non-randomised**	5.1 (1.9; 52)	6.7 (2.4; 40)	6.3 (2.6; 42)	3.2 (1.5; 42)	8.9 (2.9; 41)	9.5 (3.5; 35)
**Health Anxiety §**	**Randomised**	74.7 (4.8; 25)	82.5 (6.4; 19)	80.0 (4.5; 20)	71.7 (5.6; 20)	71.7 (5.6; 20)	78.6 (4.4; 14)
	**Non-randomised**	64.8 (3.3; 53)	71.7 (3.9; 40)	68.3 (3.4; 42)	75.4 (3.6; 42)	81.3 (3.3; 41)	75.2 (4.4; 35)
**Weight Worries §**	**Randomised**	84.6 (4.2; 26)	91.2 (3.5; 19)	95.0 (2.7; 20)	95.0 (3.6; 20)	86.7 (3.7; 20)	85.7 (5.8; 14)
	**Non-randomised**	88.1 (2.7; 53)	86.7 (2.9; 40)	85.7 (3.2; 42)	88.1 (3.2; 42)	88.6 (3.0; 41)	84.8 (3.7; 35)

**Urinary Frequency**	**Randomised**	25.0 (4.7; 26)	25.0 (6.1; 18)	25.8 (5.7; 20)	25.8 (5.1; 20)	30.7 (5.7; 19)	21.4 (6.2; 14)
	**Non-randomised**	31.4 (3.1; 53)	36.2 (4.0; 41)	31.0 (3.3; 42)	31.7 (3.9; 42)	34.1 (3.6; 41)	37.1 (4.3; 35)
**§ FUNCTION scores: higher scores = improved function (0-100); SYMPTOMS: higher scores = worse symptoms (0-100)**							
**COREFO - mean response by question^**		**Mean score (SD) N**	**Mean score (SD) N**	**Mean score (SD) N**	**Mean score (SD) N**	**Mean score (SD) N**	**Mean score (SD) N**
**Have you unintentionally passed wind?**	**Randomised**	37.0 (33.6), n=23	27.8 (27.0), n=18	45.6 (26.9), n=17	36.1 (30.0), n=18	44.7 (33.9), n=19	46.2 (30.4), n=13
	**Non-randomised**	38.4 (36.3), n=43	52.0 (35.1), n=38	54.8 (31.4), n=42	52.5 (30.4), n=40	48.8 (33.0), n=41	53.1 (36.3), n=32
**Have you unintentionally passed liquid stools during the day?**	**Randomised**	4.3 (12.3), n=23	13.9 (21.4), n=18	10.3 (26.6), n=17	5.9 (14.1), n=17	6.6 (16.3), n=19	17.3 (32.9), n=13
	**Non-randomised**	7.0 (19.2), n=43	31.6 (34.7), n=38	23.2 (28.9), n=42	18.8 (28.7), n=40	15.0 (23.9), n=40	13.3 (21.0), n=32
**Have you unintentionally passed liquid stools during the night?**	**Randomised**	0.0 (0.0), n=23	6.9 (16.7), n=18	0.0 (0.0), n=17	2.9 (12.1), n=17	6.6 (16.3), n=19	5.4 (14.5), n=14
	**Non-randomised**	3.5 (12.9), n=43	6.8 (19.2), n=37	3.6 (11.8), n=42	5.0 (14.1), n=40	3.8 (12.1), n=40	5.5 (16.5), n=32
**Have you unintentionally passed solid stools during the day?**	**Randomised**	3.3 (11.4), n=23	16.2 (30.5), n=17	8.8 (26.4), n=17	8.8 (24.9), n=17	7.9 (20.5), n=19	5.8 (15.0), n=13
	**Non-randomised**	2.9 (13.6), n=43	16.0 (30.6), n=36	10.7 (24.8), n=42	12.5 (23.3), n=40	11.9 (21.2), n=40	10.2 (21.9), n=32
**Have you unintentionally passed solid stools during the night?**	**Randomised**	1.1 (5.2), n=23	2.9 (12.1), n=17	0.0 (0.0), n=17	0.0 (0.0), n=17	5.3 (17.8), n=19	0.0 (0.0), n=13
	**Non-randomised**	1.2 (7.6), n=43	1.4 (8.3), n=36	1.2 (7.7), n=42	1.2 (7.9), n=40	1.9 (8.7), n=40	1.6 (8.8), n=32
**Have you had a smear of faeces in your underwear during the day?**	**Randomised**	10.4 (19.4), n=24	19.4 (30.4), n=18	17.2 (29.9), n=16	15.8 (26.6), n=19	18.8 (30.2), n=20	30.4 (34.2), n=14
	**Non-randomised**	24.4 (30.8), n=45	43.9 (35.1), n=37	35.4 (32.6), n=41	37.5 (32.5), n=40	33.8 (32.8), n=40	35.3 (33.2), n=34
**Have you had a smear of faeces in your underwear, pyjamas or night-gown at the end of the night?**	**Randomised**	7.3 (17.3), n=24	9.7 (25.9), n=18	5.9 (16.6), n=17	0.0 (0.0), n=19	7.9 (16.8), n=19	16.1 (28.8), n=14
	**Non-randomised**	5.6 (16.8), n=45	16.9 (26.4), n=37	8.5 (16.4), n=41	10.6 (21.8), n=40	13.8 (21.9), n=40	16.2 (31.9), n=34
**Was it difficult to distinguish between passing wind and a bowel movement?**	**Randomised**	9.4 (19.2), n=24	16.7 (29.7), n=18	13.2 (21.9), n=17	18.4 (20.1), n=19	13.2 (24.1), n=19	23.1 (33.0), n=13
	**Non-randomised**	22.8 (30.5), n=45	37.2 (37.6), n=37	25.0 (31.1), n=41	25.0 (30.0), n=40	16.0 (22.6), n=39	19.1 (32.6), n=34
**Have you used something to protect your underwear, such as sanitary towels, panty liners or nappies?**	**Randomised**	13.1 (29.2), n=21	38.9 (43.9), n=18	26.5 (41.0), n=17	25.0 (36.3), n=19	18.4 (29.9), n=19	17.9 (22.8), n=14
	**Non-randomised**	20.2 (37.8), n=47	68.2 (44.0), n=37	56.1 (46.4), n=41	46.9 (44.3), n=40	39.6 (46.4), n=41	44.9 (46.4), n=34

**If you needed to go urgently, did you have trouble stopping your bowel movement for longer than 15 min?**	**Randomised**	25.0 (27.6), n=24	39.7 (39.6), n=17	23.5 (32.4), n=17	14.7 (28.0), n=17	38.2 (34.4), n=17	34.6 (31.5), n=13
	**Non-randomised**	16.8 (25.8), n=46	35.0 (34.4), n=35	38.8 (35.8), n=40	39.7 (33.8), n=39	36.9 (29.9), n=42	34.1 (34.7), n=33
**Have you had a false alarm? (i.e. a need to go without a bowel movement)?**	**Randomised**	9.4 (23.1), n=24	14.7 (19.9), n=17	8.8 (19.6), n=17	7.8 (12.0), n=16	19.1 (25.8), n=17	21.2 (28.6), n=13
	**Non-randomised**	19.1 (26.2), n=47	31.8 (35.2), n=37	23.8 (29.4), n=40	17.5 (26.7), n=40	14.3 (21.5), n=42	20.6 (28.5), n=34
**When you went to the toilet, did your bowel movement require more than 15 min?**	**Randomised**	7.3 (17.3), n=24	6.9 (24.0), n=18	2.9 (12.1), n=17	7.4 (21.2), n=17	15.0 (29.7), n=20	23.2 (36.0), n=14
	**Non-randomised**	7.2 (18.9), n=45	8.1 (18.7), n=37	12.2 (21.7), n=41	10.0 (21.8), n=40	10.6 (17.8), n=40	6.1 (17.7), n=33
**Did you feel that your bowels were not empty after your bowel movement?**	**Randomised**	26.0 (26.0), n=24	33.3 (35.4), n=18	36.8 (34.4), n=17	36.8 (35.5), n=17	40.0 (30.8), n=20	44.6 (32.8), n=14
	**Non-randomised**	30.0 (31.8), n=45	42.6 (33.3), n=37	36.6 (29.6), n=41	35.6 (29.4), n=40	30.0 (31.6), n=40	39.0 (32.1), n=34
**After you had a bowel movement, did you have to return to the toilet within 1hour for a bowel movement?**	**Randomised**	23.9 (29.4), n=22	39.7 (34.3), n=17	30.9 (32.5), n=17	25.0 (33.2), n=18	35.5 (32.6), n=19	36.5 (36.3), n=13
	**Non-randomised**	26.1 (27.7), n=45	36.8 (35.1), n=36	40.0 (31.9), n=40	39.4 (29.9), n=40	36.2 (32.5), n=40	38.3 (31.1), n=32
**Did you adjust your activities to the availability of a toilet?**	**Randomised**	6.8 (19.2), n=22	37.5 (40.4), n=18	33.8 (38.5), n=17	27.6 (35.3), n=19	26.3 (32.8), n=19	40.4 (33.1), n=13
	**Non-randomised**	16.0 (26.8), n=47	41.9 (42.9), n=37	32.3 (37.6), n=41	26.9 (34.6), n=40	25.0 (34.9), n=41	32.8 (37.8), n=32
**Were you limited in your daily activities (e.g. work or house work) due to problems with your bowel movements?**	**Randomised**	6.8 (17.6), n=22	15.3 (33.4), n=18	10.3 (26.6), n=17	2.6 (11.5), n=19	7.9 (20.5), n=19	8.9 (21.0), n=14
	**Non-randomised**	5.3 (18.0), n=47	27.7 (41.2), n=37	23.2 (36.0), n=41	6.9 (22.6), n=40	12.8 (28.0), n=41	7.9 (19.0), n=35
**Were you limited in your social activities (e.g. family visits, visits to the theatre, or eating out) due to problems with your bowel movements?**	**Randomised**	5.7 (18.8), n=22	25.0 (39.3), n=18	16.2 (34.2), n=17	6.6 (14.0), n=19	13.2 (29.3), n=19	21.4 (33.8), n=14
	**Non-randomised**	9.0 (22.4), n=47	34.0 (40.2), n=36	20.8 (32.6), n=42	20.6 (32.5), n=40	15.9 (26.1), n=41	19.3 (32.7), n=35
**Were you limited in your sexual activities (with or without sexual intercourse) due to problems with your bowel movements?**	**Randomised**	5.0 (22.4), n=20	3.1 (12.5), n=16	4.7 (13.6), n=16	1.7 (6.5), n=15	0.0 (0.0), n=15	5.8 (15.0), n=13
	**Non-randomised**	9.7 (28.8), n=36	19.2 (37.0), n=30	13.8 (35.1), n=29	19.4 (36.3), n=31	10.9 (30.4), n=32	8.0 (23.6), n=28
	**Randomised**	20.8 (15.9), n=24	30.6 (22.0), n=18	23.6 (21.8), n=18	25.0 (23.4), n=17	23.6 (18.1), n=18	23.1 (12.3), n=13

**How many bowel movements have you had during the day?**	**Non-randomised**	20.7 (21.1), n=47	33.6 (22.0), n=38	25.6 (22.4), n=42	28.1 (18.9), n=40	25.0 (14.8), n=41	24.3 (17.7), n=35
**How many bowel movements have you had during the night?**	**Randomised**	0.0 (0.0), n=24	5.6 (10.7), n=18	4.2 (9.6), n=18	7.4 (11.7), n=17	12.5 (21.4), n=18	5.8 (11.0), n=13
	**Non-randomised**	4.3 (13.1), n=47	7.4 (14.3), n=37	6.0 (10.8), n=42	6.4 (13.7), n=39	5.5 (11.9), n=41	7.9 (16.9), n=35
**Have you had pain during your bowel movements?**	**Randomised**	5.2 (18.0), n=24	6.9 (16.7), n=18	0.0 (0.0), n=18	0.0 (0.0), n=18	5.6 (16.2), n=18	9.6 (21.7), n=13
	**Non-randomised**	11.2 (23.8), n=47	13.8 (28.3), n=38	7.5 (15.2), n=40	6.9 (16.0), n=40	7.1 (16.8), n=42	8.6 (22.6), n=35
**Have you experienced blood loss during your bowel movements?**	**Randomised**	30.4 (32.8), n=23	0.0 (0.0), n=18	0.0 (0.0), n=18	2.8 (11.8), n=18	1.4 (5.9), n=18	0.0 (0.0), n=13
	**Non-randomised**	31.9 (34.5), n=47	6.6 (16.1), n=38	4.5 (11.3), n=39	5.0 (14.1), n=40	4.8 (15.8), n=42	5.7 (16.1), n=35
**Have you had irritated skin around your anus?**	**Randomised**	9.1 (18.2), n=22	16.7 (30.9), n=18	10.3 (25.1), n=17	3.9 (17.2), n=19	2.6 (11.5), n=19	8.9 (23.2), n=14
	**Non-randomised**	8.7 (19.2), n=46	20.3 (30.0), n=37	16.2 (23.0), n=40	20.6 (29.9), n=40	17.5 (30.6), n=40	19.1 (32.6), n=34
**Have you used medicines to thicken your stools?**	**Randomised**	1.1 (5.2), n=23	7.4 (17.1), n=17	2.9 (8.3), n=17	7.9 (25.1), n=19	13.2 (32.7), n=19	21.4 (42.6), n=14
	**Non-randomised**	1.1 (5.2), n=45	13.2 (30.8), n=36	15.0 (33.4), n=40	11.9 (31.0), n=40	17.5 (34.5), n=40	12.9 (33.1), n=33
**Have you eaten certain foods on purpose to make your stools thicker or thinner?**	**Randomised**	0.0 (0.0), n=23	27.9 (41.3), n=17	13.2 (33.2), n=17	13.2 (26.8), n=19	14.5 (30.4), n=19	19.6 (32.8), n=14
	**Non-randomised**	18.3 (32.6), n=45	17.4 (33.2), n=36	9.8 (24.3), n=41	11.2 (24.6), n=40	18.1 (28.3), n=40	6.1 (19.8), n=33
**Have you purposely avoided certain foods to prevent your stools becoming loose or hard?**	**Randomised**	8.7 (25.7), n=23	20.8 (31.2), n=18	2.9 (12.1), n=17	18.4 (26.1), n=19	13.2 (26.8), n=19	21.4 (32.3), n=14
	**Non-randomised**	15.6 (27.3), n=45	17.4 (29.2), n=36	17.7 (31.2), n=41	24.4 (36.9), n=40	25.0 (35.8), n=40	9.1 (21.5), n=33

* EORTC Norm Cohort: Age-matched UK general population data; ^ Scores with patients with and without a stoma combined; Scoring: For scaled items, if at least half of the items from the scale were answered, it was assumed the missing items have values equal to the average of those items which are present for that respondent. *(EORTC QLQ-C30 scoring Manual).*

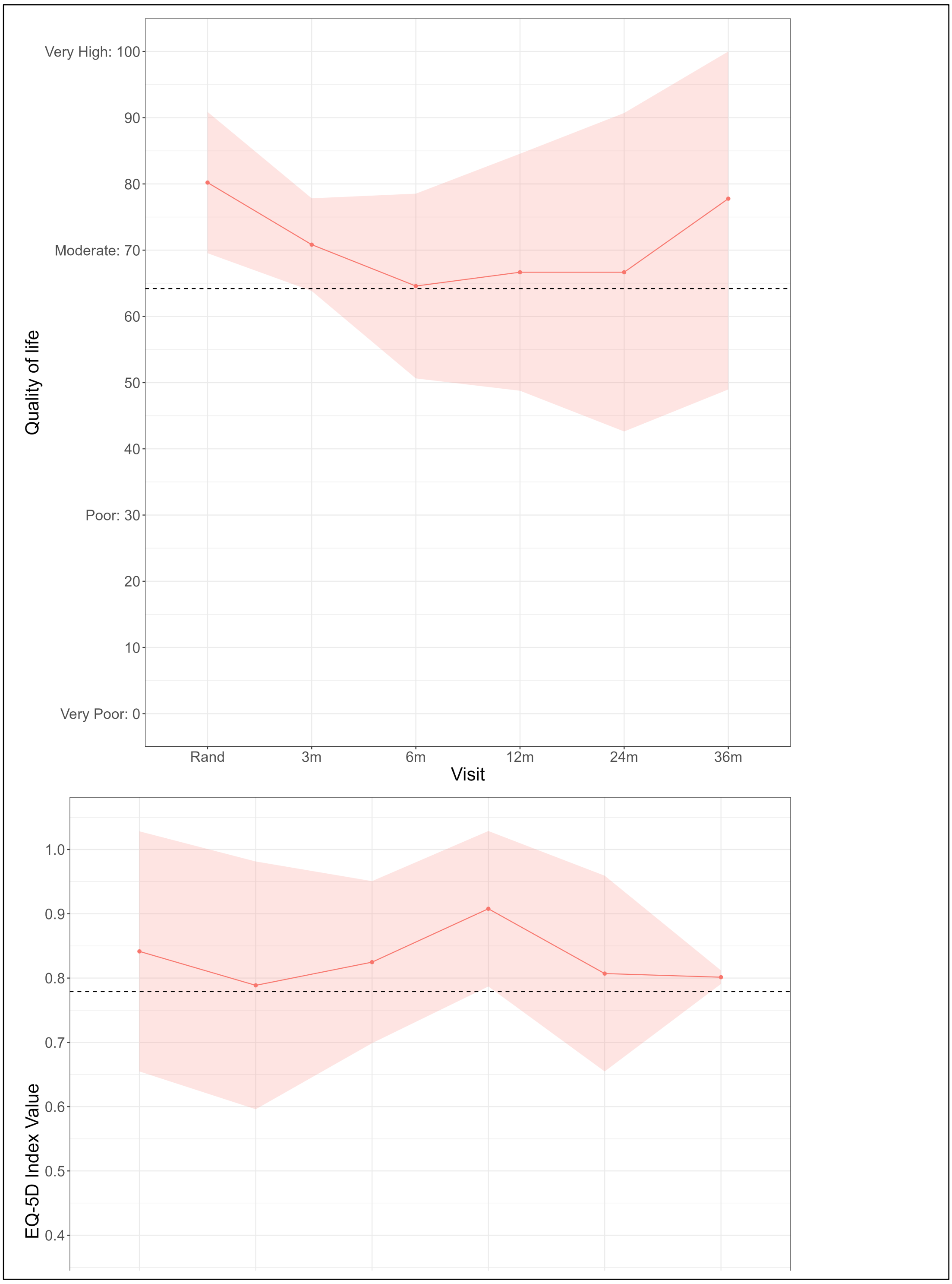

Data on patients who converted from organ preservation to TME surgery

Supplementary Figure 1: Overall QOL and EQ5D scores over time for patients (randomised and non-randomised) who received organ-preservation and were then converted to TME surgery (Dotted line = UK norm data)

Supplementary Table 2: Scores for patients who converted from organ preservation to TME surgery

Pooled EORTC QLQ C30 and EQ5D5L summary index scores of converters (completed SCRT + TEMS and then converted to TME)						
	**Baseline**	**3 months**	**6 months**	**12 months**	**24 months**	**36 months**
**Item**	**Mean score (SE; N)**	**Mean score (SE; N)**	**Mean score (SE; N)**	**Mean score (SE; N)**	**Mean score (SE; N)**	**Mean score (SE; N)**
**Global health status §**	80.2 (5.4; 8)	70.8 (3.6; 6)	64.6 (7.1; 4)	66.7 (9.1; 5)	66.7 (12.3; 4)	77.8 (14.7; 3)
**EQ5D Index Value**	0.84 (0.10; 8)	0.79 (0.10; 6)	0.82 (0.06; 4)	0.91 (0.06; 5)	0.81 (0.08; 4)	0.80 (0.01; 3)

§ FUNCTION scores: higher scores = improved function (0-100)

Review of Baseline Scores by Patient Characteristics

EORTC QLQ-C30

Figures 1-5 respectively present all baseline EORTC QLQ-C30 outcomes by mechanism of entry (randomised vs non-randomised, figure 1) patients age at entry to TREC (figure 2), sex (figure 3), T stage (figure 4), and tumour height (figure 5). These figures are presented for the pooled population of all patients and then according to mechanism of entry to the trial (figures 2-5).

Visual inspection of figures 1-5 revealed no discernible difference in outcomes dependant on any factor.

This is further verified by the univariate analyses of EORTC QLQ-C30 items, QL2 and the summary score, exploring the relationship between each of the aforementioned factors and baseline EORTC scores (Table 1). These univariate analyses were performed using analogous methodology described in the main paper. Global quality of life (QL2) and the summary score were selected as representative scaled items, in particular the summary score was chosen due to its encompassing of the majority of items from EORTC QLQ-C30.

All effects were seen to be of small magnitude and containing the null value, and so it was concluded none were important at predicting PRO poor responses at baseline.

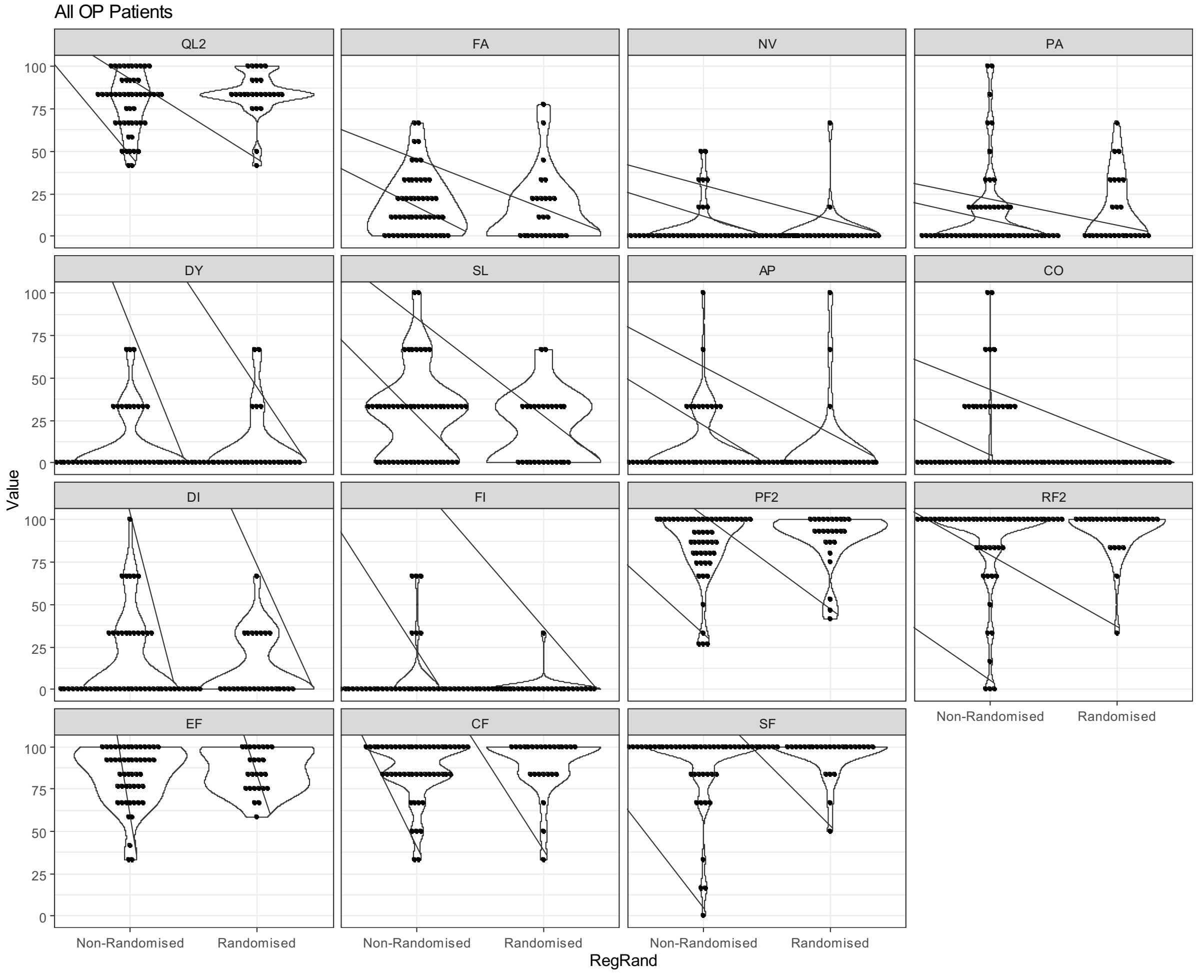

Figure 2: Baseline EORTC QLQ-C30 scores by mechanism of entry to TREC

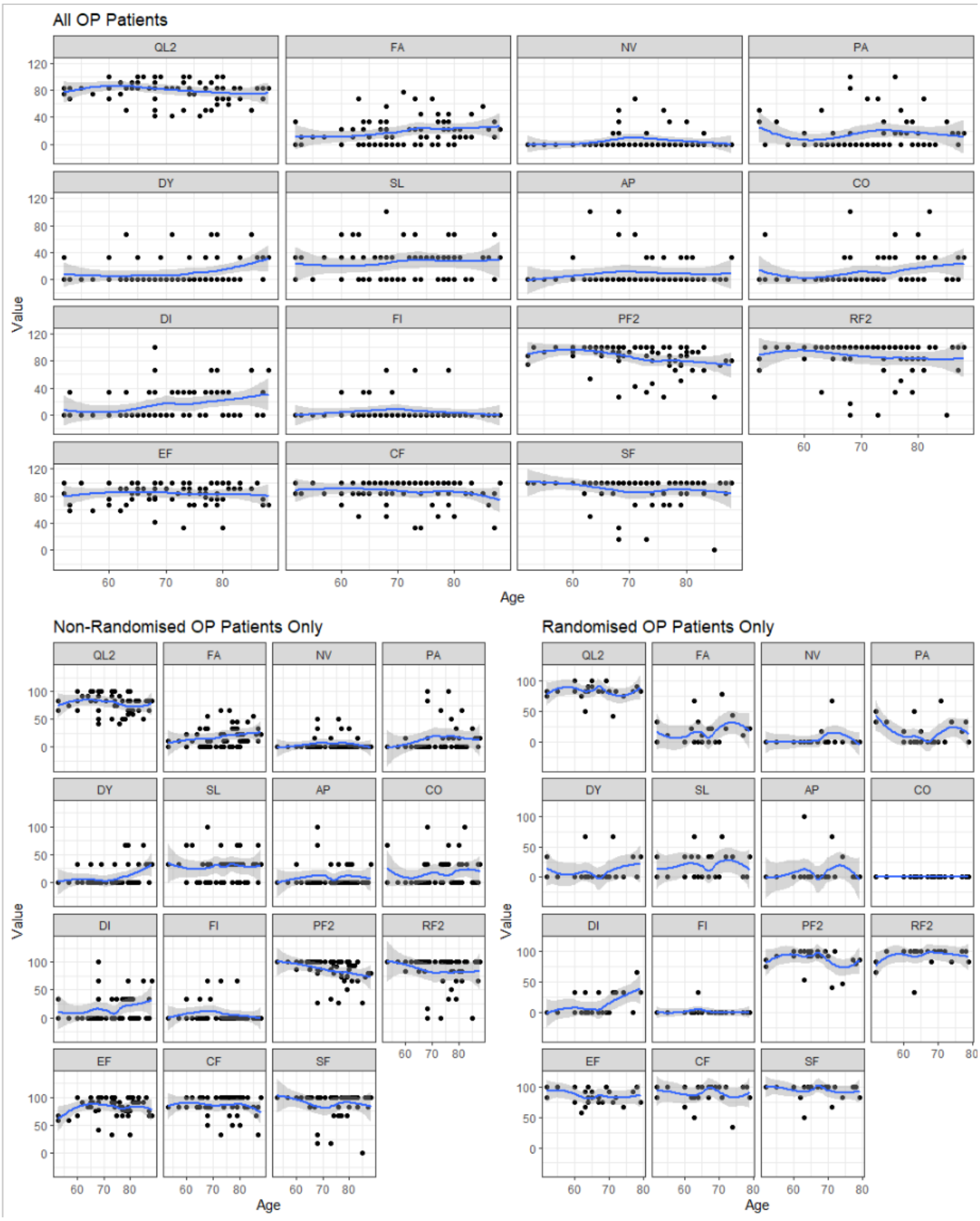

Figure 3: Baseline EORTC QLQ-C30 scores by age to TREC trial

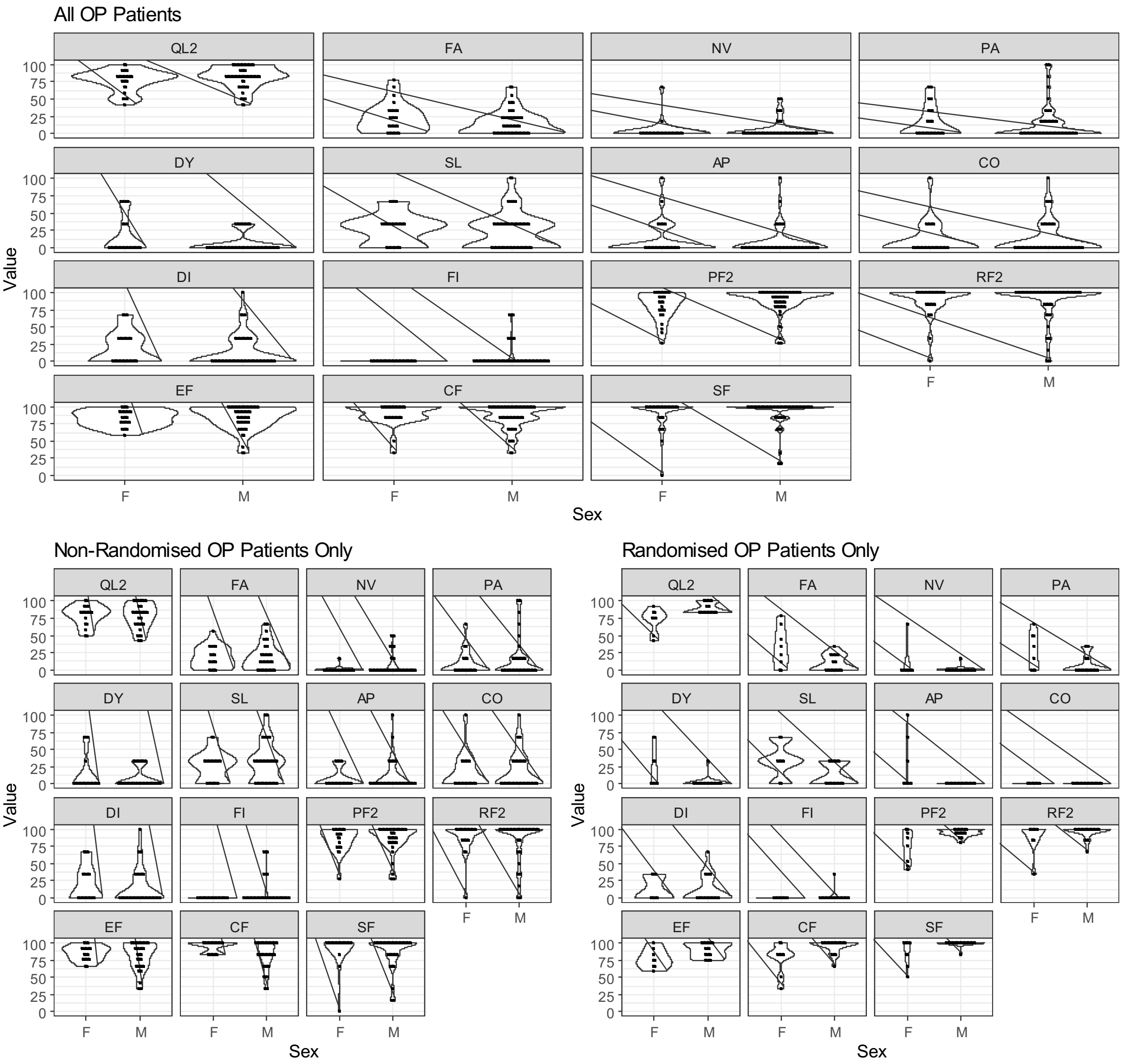

Figure 4: Baseline EORTC QLQ-C30 scores by sex

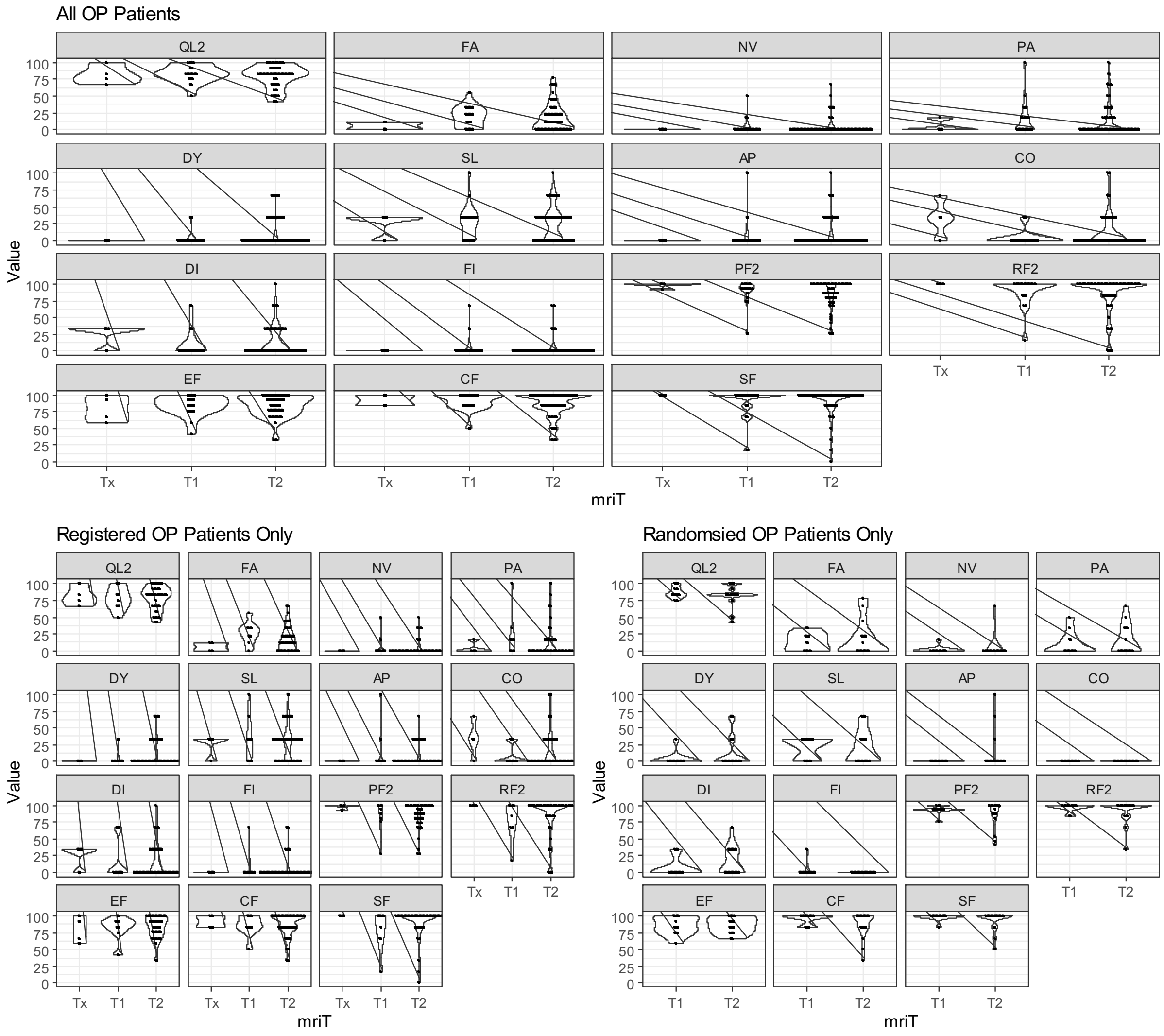

Figure 5: Baseline EORTC QLQ-C30 scores by MRI T stage

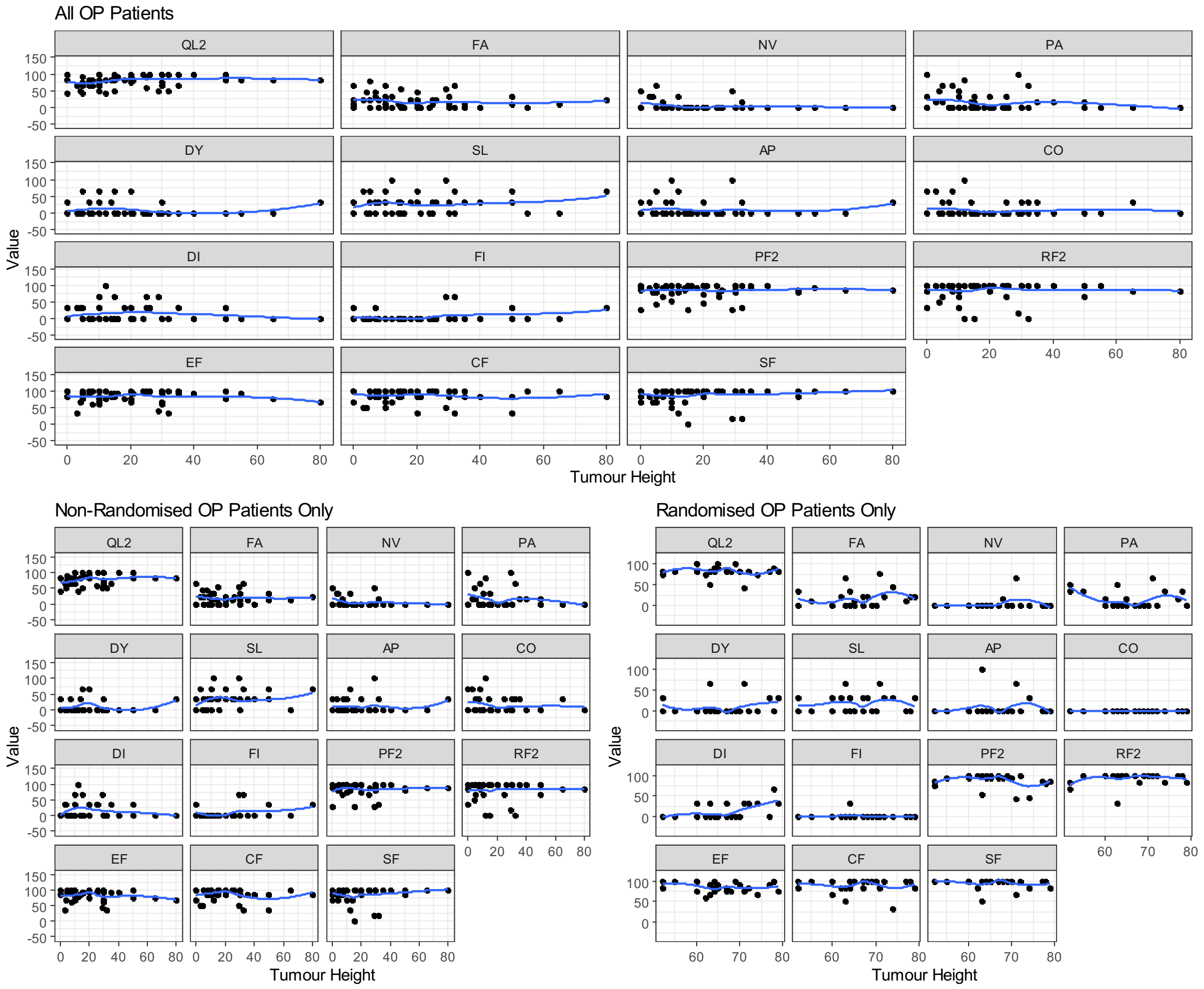

Figure 6: Baseline EORTC QLQ-C30 scores by tumour height

Table 3: Results of the Univariate Analysis for EORTC QLQ-C30

**Factors**	**Effect estimate**
**Summary Score**	
Mechanism of entry *(Randomised as the reference category)*	-5.01 (95% CrI: -11.96, 1.77)
Age (years)	-0.39 (95% CrI: -0.76, -0.03)
Sex *(Female as the reference category)*	3.17 (95% CrI: -3.63, 10.14)
MRI T Staging *(Tx as the reference category)*	T1: -3.82 (95% CrI: -16.20, 8.49)<br>T2: -0.01 (95% CrI: -8.87, 8.94)
Tumour height	0.06 (95% CrI: -0.17, 0.30)
**QL2**	
Mechanism of entry *(Randomised as the reference category)*	-0.51 (95% CrI: -1.36, 0.35)
Age (years)	-0.04 (95% CrI: -0.08, 0.01)
Sex *(Female as the reference category)*	0.60 (95% CrI: -0.24, 1.47)
MRI T Staging *(Tx as the reference category)*	T1: 0.04 (95% CrI: -1.34, 1.34)<br>T2: -0.21 (95% CrI: -1.21, 0.81)
Tumour height	0.02 (95% CrI: 0.00, 0.05)

Note: CrI - Credible Interval

EORTC QLQ-CR29

Figures 6-10 respectively present all baseline EORTC QLQ-CR29 outcomes by mechanism of entry (randomised vs non-randomised, figure 6) patients age at entry to TREC (figure 7), sex (figure 8), T stage (figure 9), and height of tumour (figure 10). These figures are presented for the pooled population of all patients and then according to mechanism of entry to the trial (figures 7-10).

Visual inspection of figures 6-10 revealed no discernible difference in outcomes dependant on any factor.

As there is no summary measure derived from the EORTC QLQ-CR29, no univariate analyses were explored.

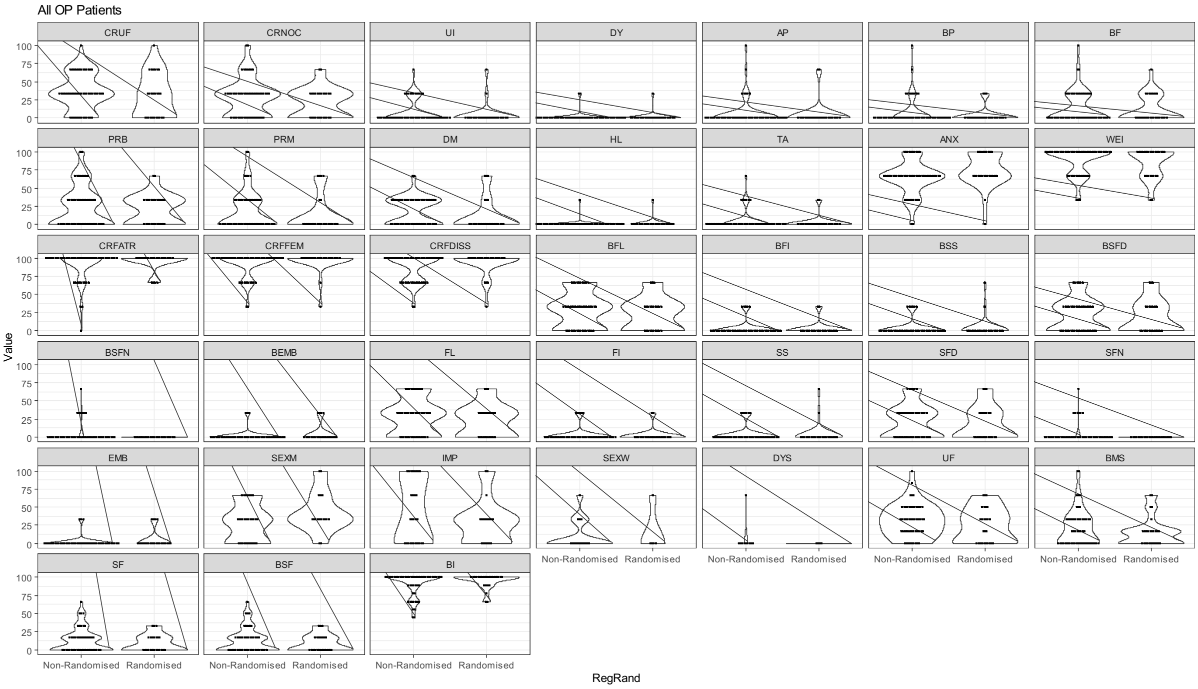

Figure 7: Baseline EORTC QLQ-CR29 scores by mechanism of entry to TREC

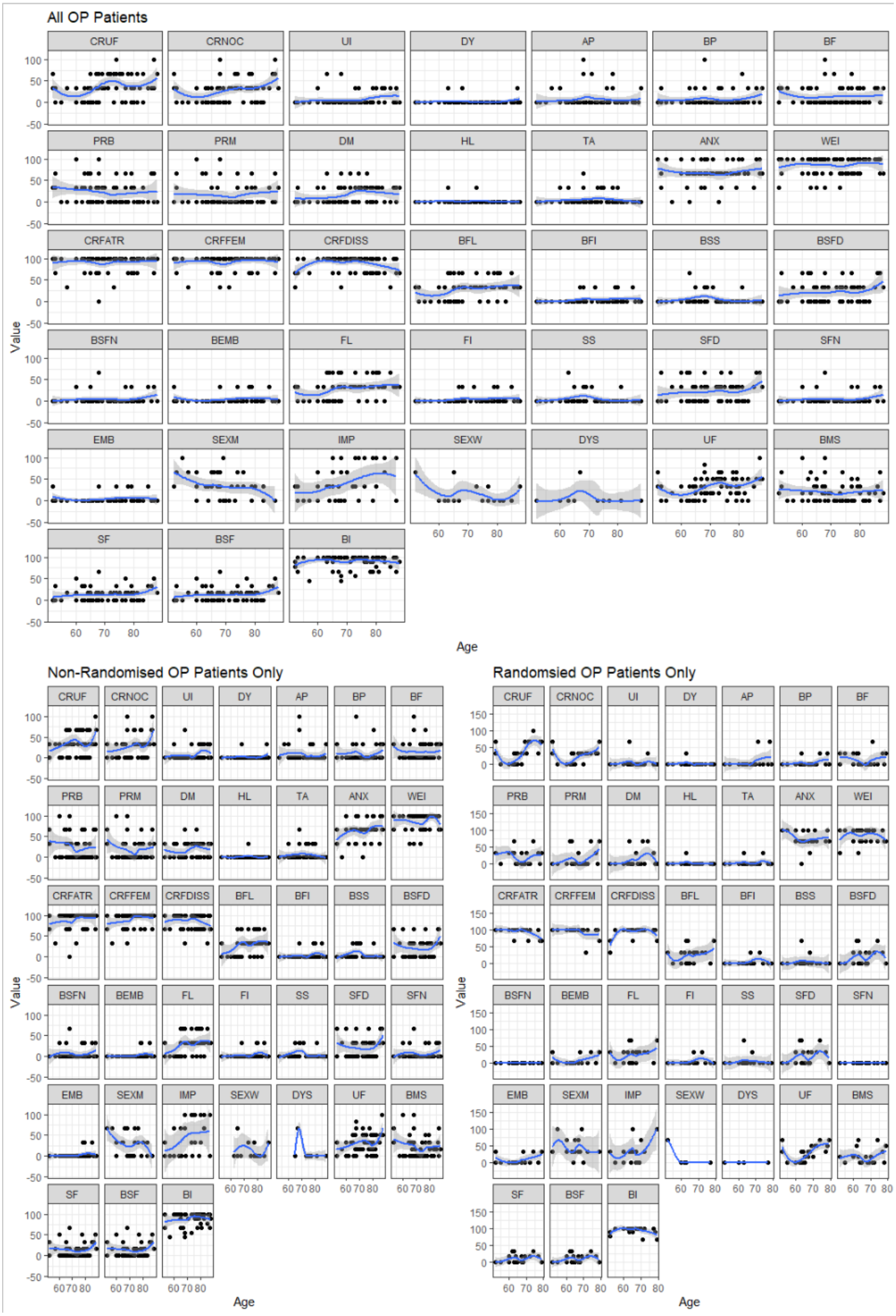

Figure 8: Baseline EORTC QLQ-CR29 scores by Age

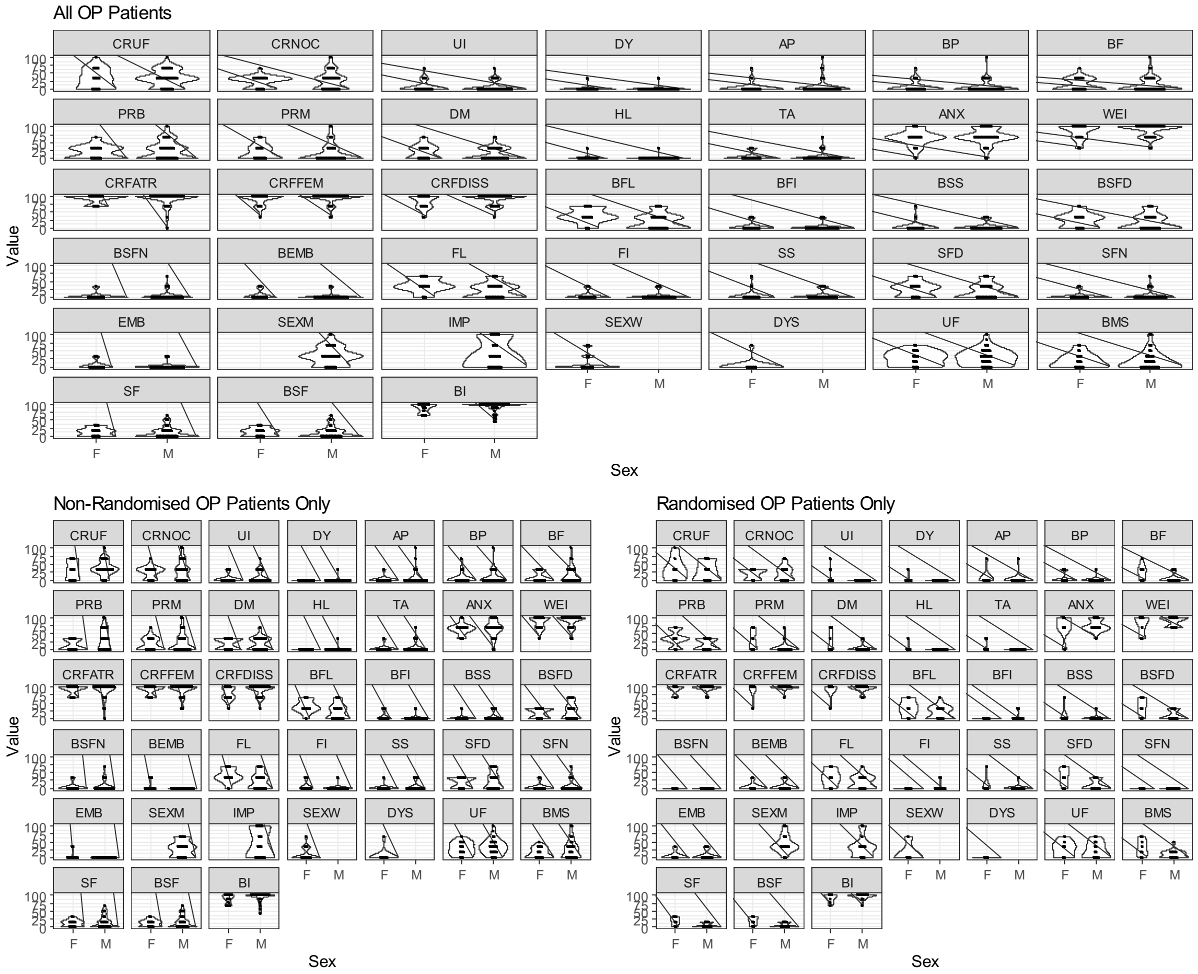

Figure 9: Baseline EORTC QLQ-CR29 scores by Sex

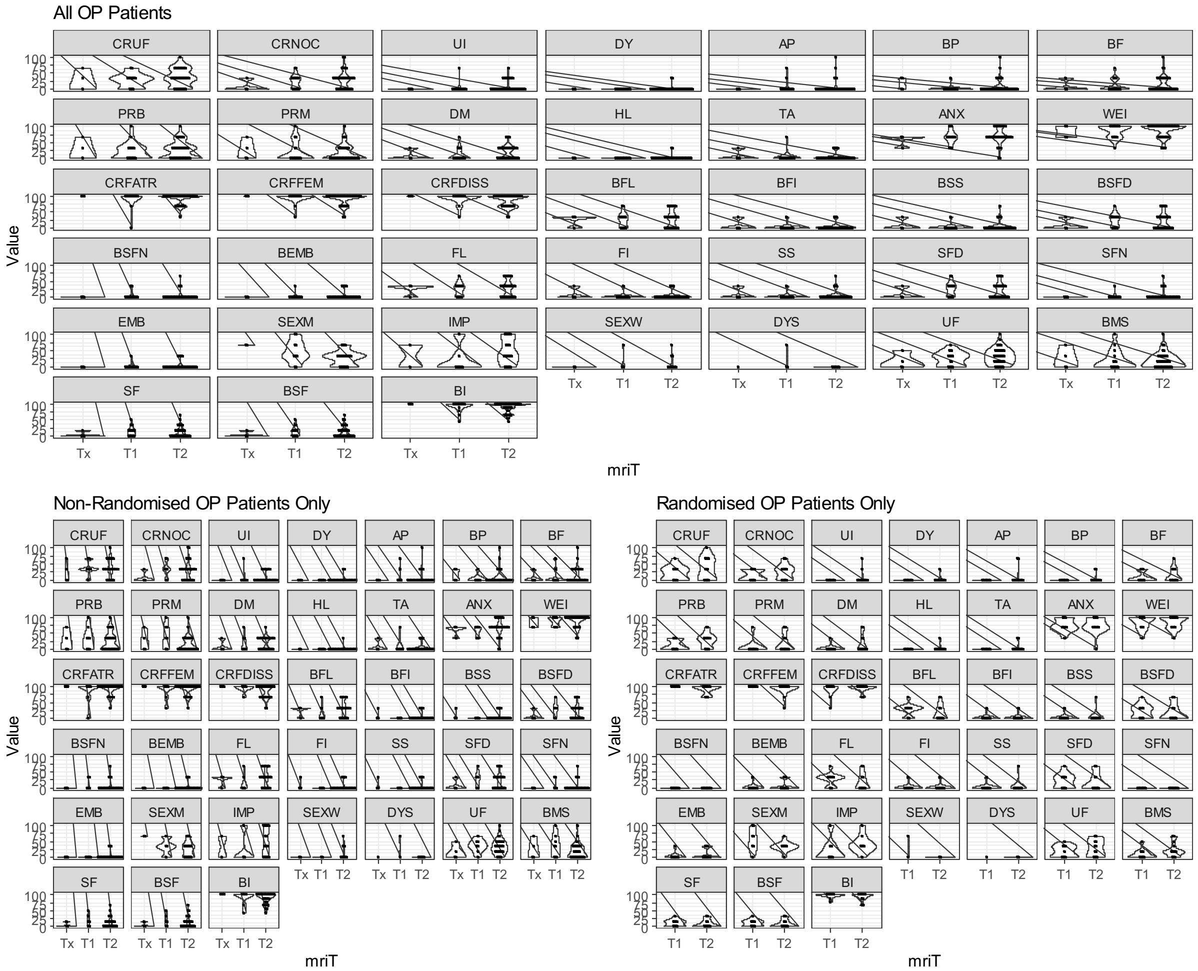

Figure 10: Baseline EORTC QLQ-CR29 scores by MRI T Stage

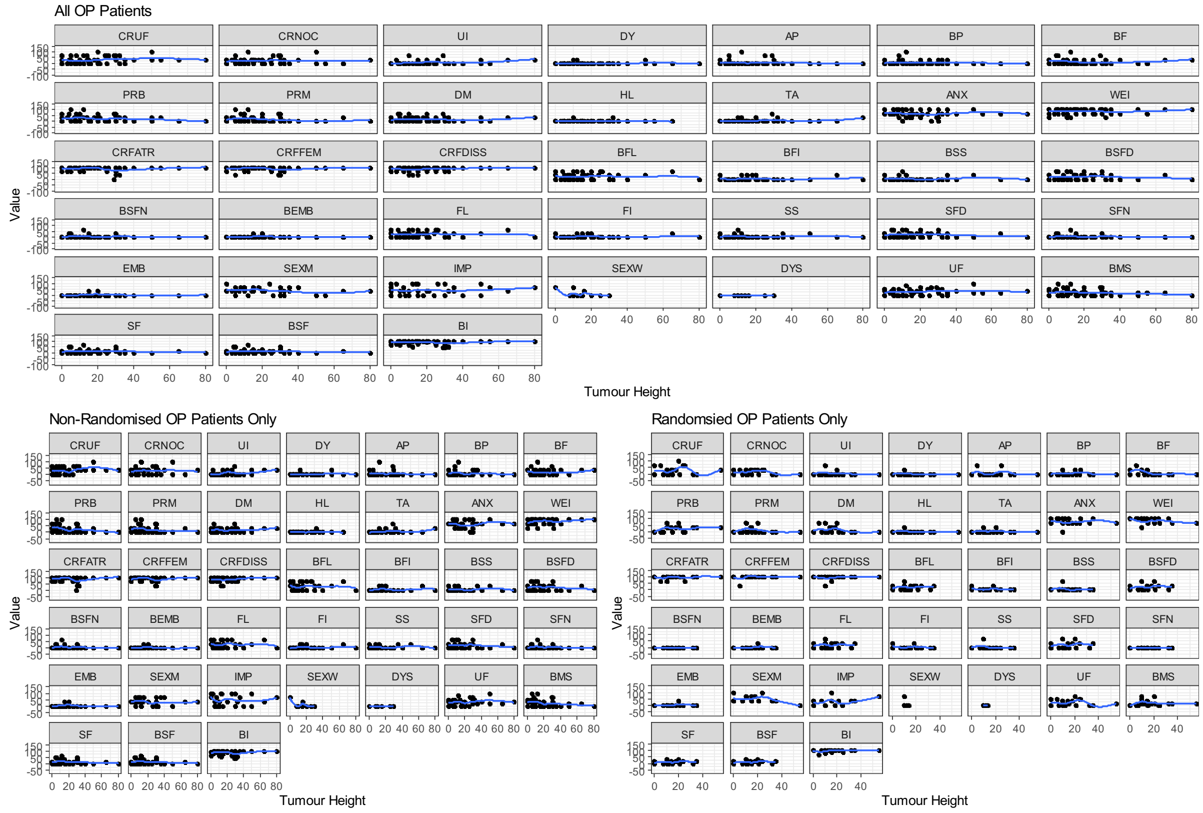

Figure 11: Baseline EORTC QLQ-CR29 scores by Tumour height

COREFO Items

Figures 11-15 respectively present all baseline COREFO outcomes by mechanism of entry (randomised vs non-randomised, figure 11) patients age at entry to TREC (figure 12), sex (figure 13), T stage (figure 14), and tumour height (figure 15). These figures are presented for the pooled population of all patients and then according to mechanism of entry to the trial (figures 12-15 only).

Visual inspection of figures 11-15 revealed no discernible difference in outcomes dependant on any factor.

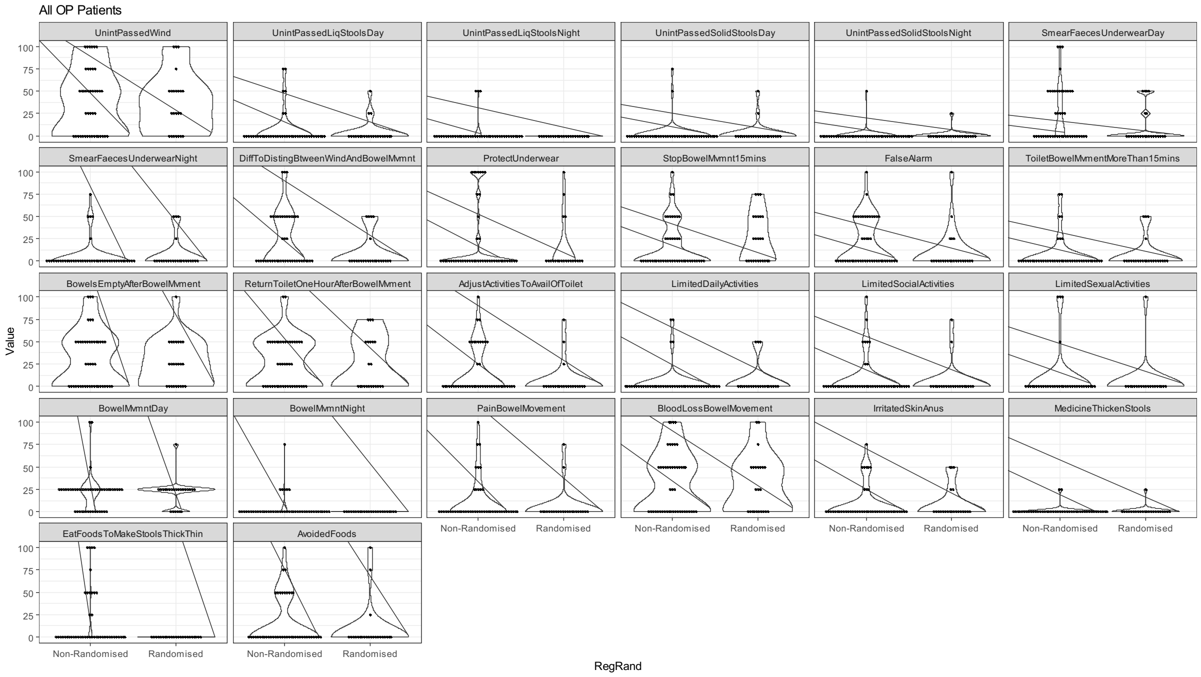

Figure 12: Baseline COREFO outcomes by mechanism of entry to TREC

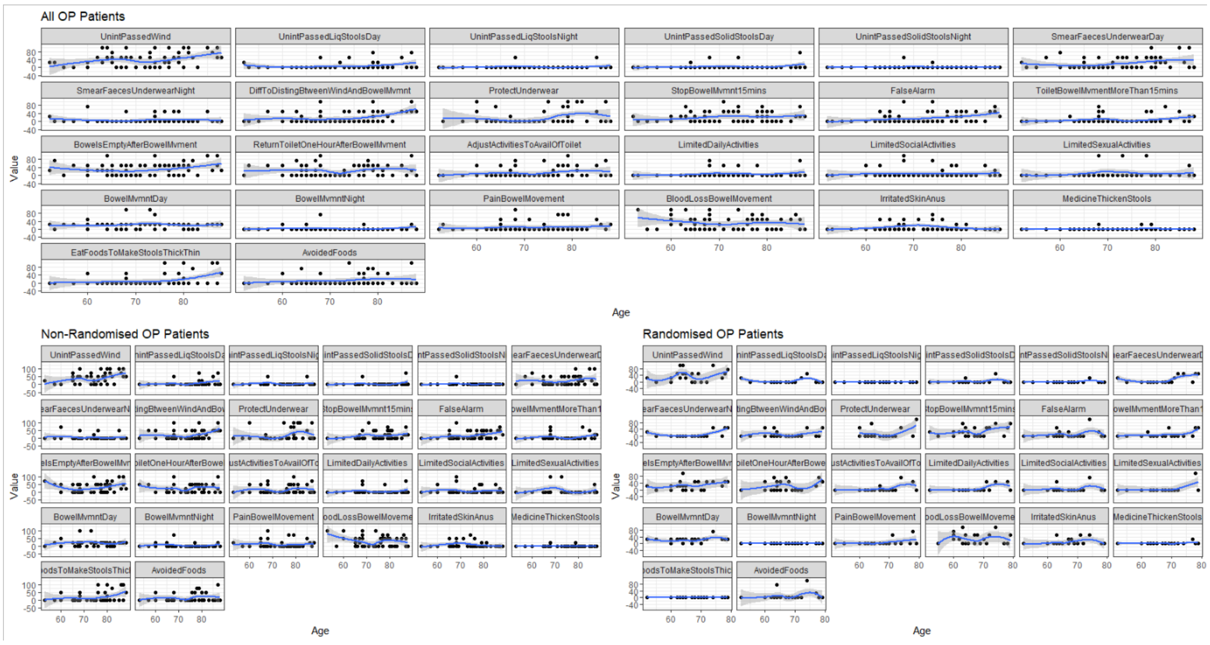

Figure 13:Baseline COREFO outcomes by age

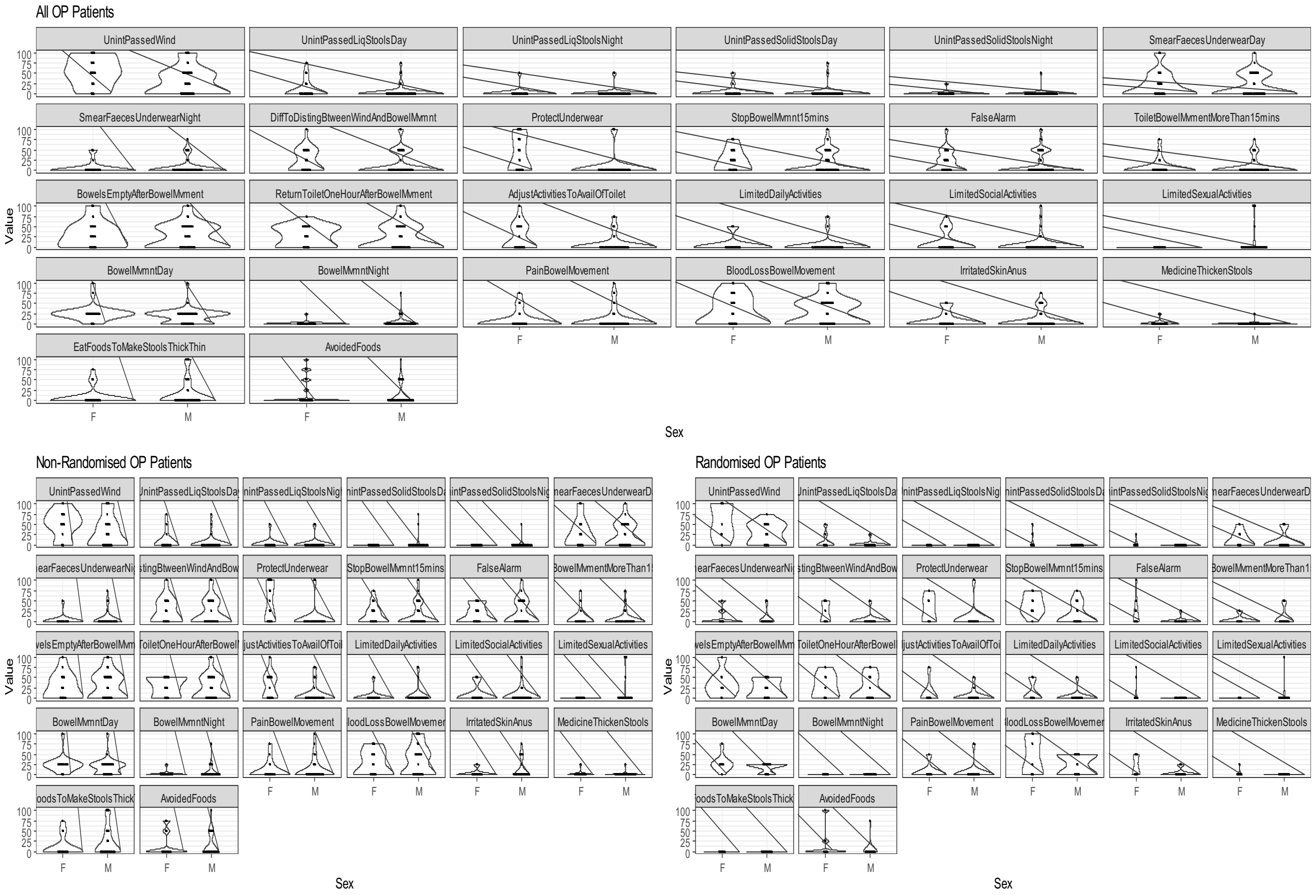

Figure 14: Baseline COREFO outcomes by sex

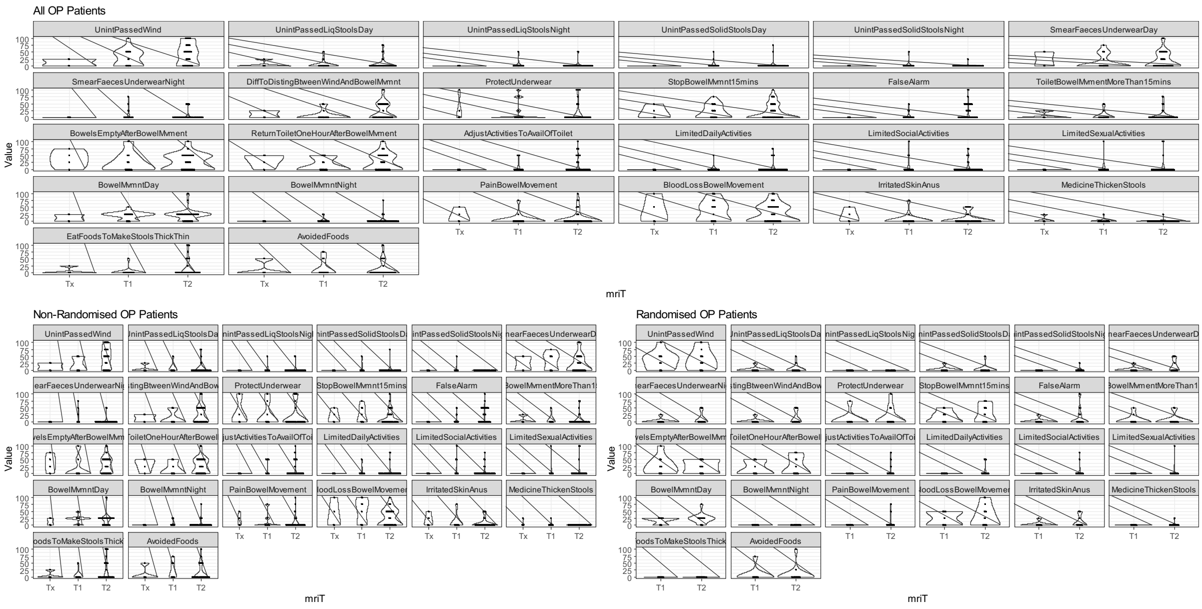

Figure 15: Baseline COREFO outcomes by MRI T stage

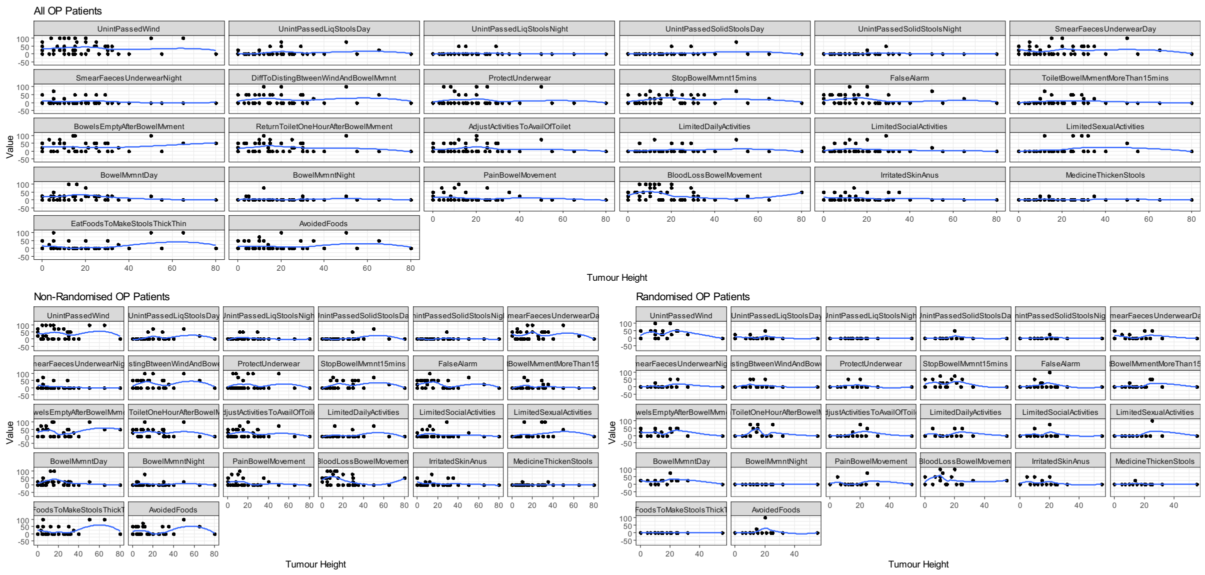

Figure 16: Baseline COREFO outcomes by tumour height

EQ5D Index Value

Figures 16-20 respectively present all baseline EQ5D index values by mechanism of entry (randomised vs non-randomised, figure 16) patients age at entry to TREC (figure 17), sex (figure 18), T stage (figure 19), and tumour height (figure 20). These figures are presented for the pooled population of all patients and then according to mechanism of entry to the trial (figures 16-20).

Visual inspection of figures 16-20 revealed no discernible difference in outcomes dependant on any factor.

This is further verified by the univariate analyses of the index value exploring the relationship between each of the aforementioned factors (Table 2). These univariate analyses were performed using analogous methodology described in the main paper. All effects were seen to be of small magnitude and containing the null value, and so it was concluded none were important at predicting PRO poor responses at baseline.

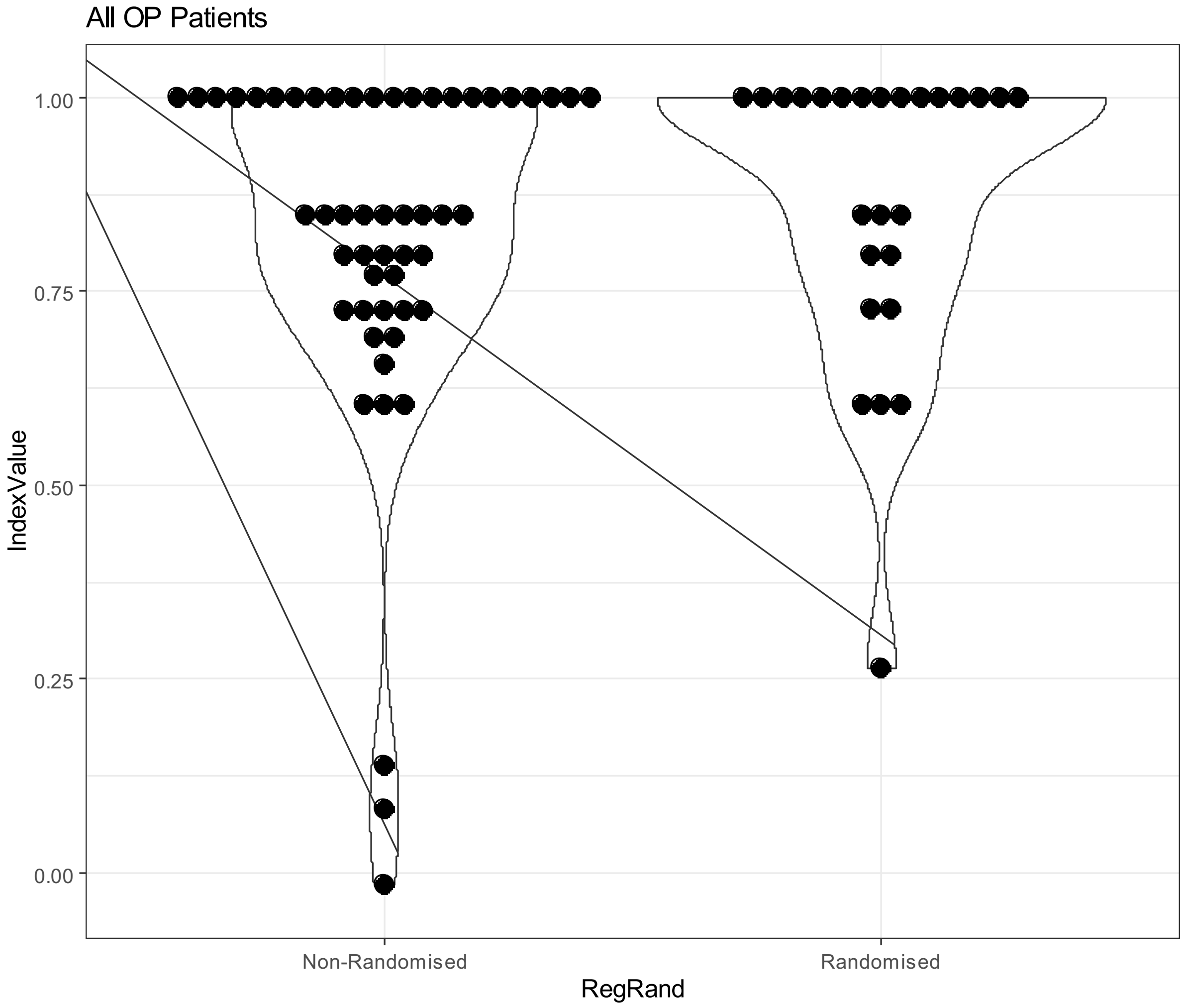

Figure 17: Baseline EQ5D Index Value by mechanism of entry to TREC

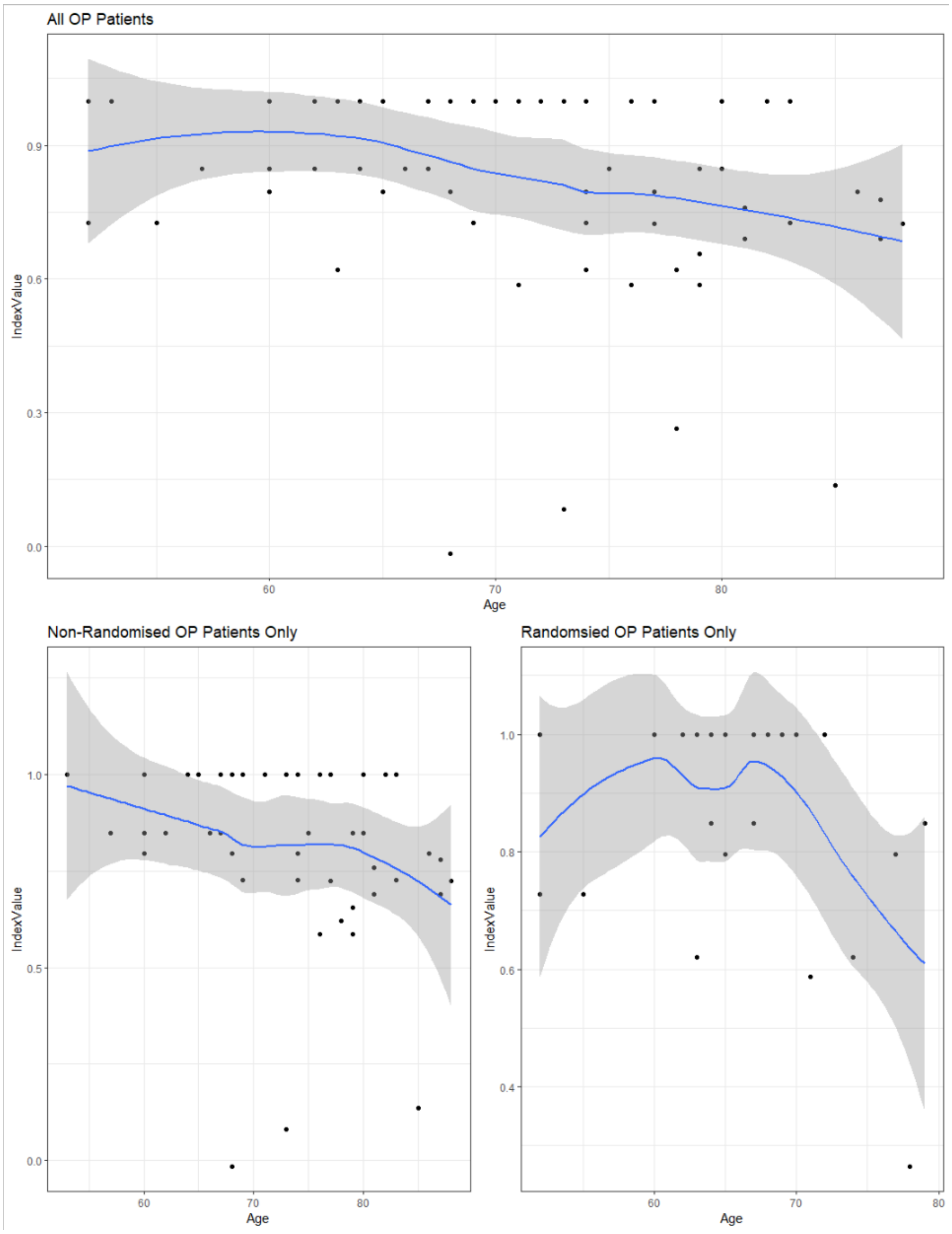

Figure 18: Baseline EQ5D Index Value by age

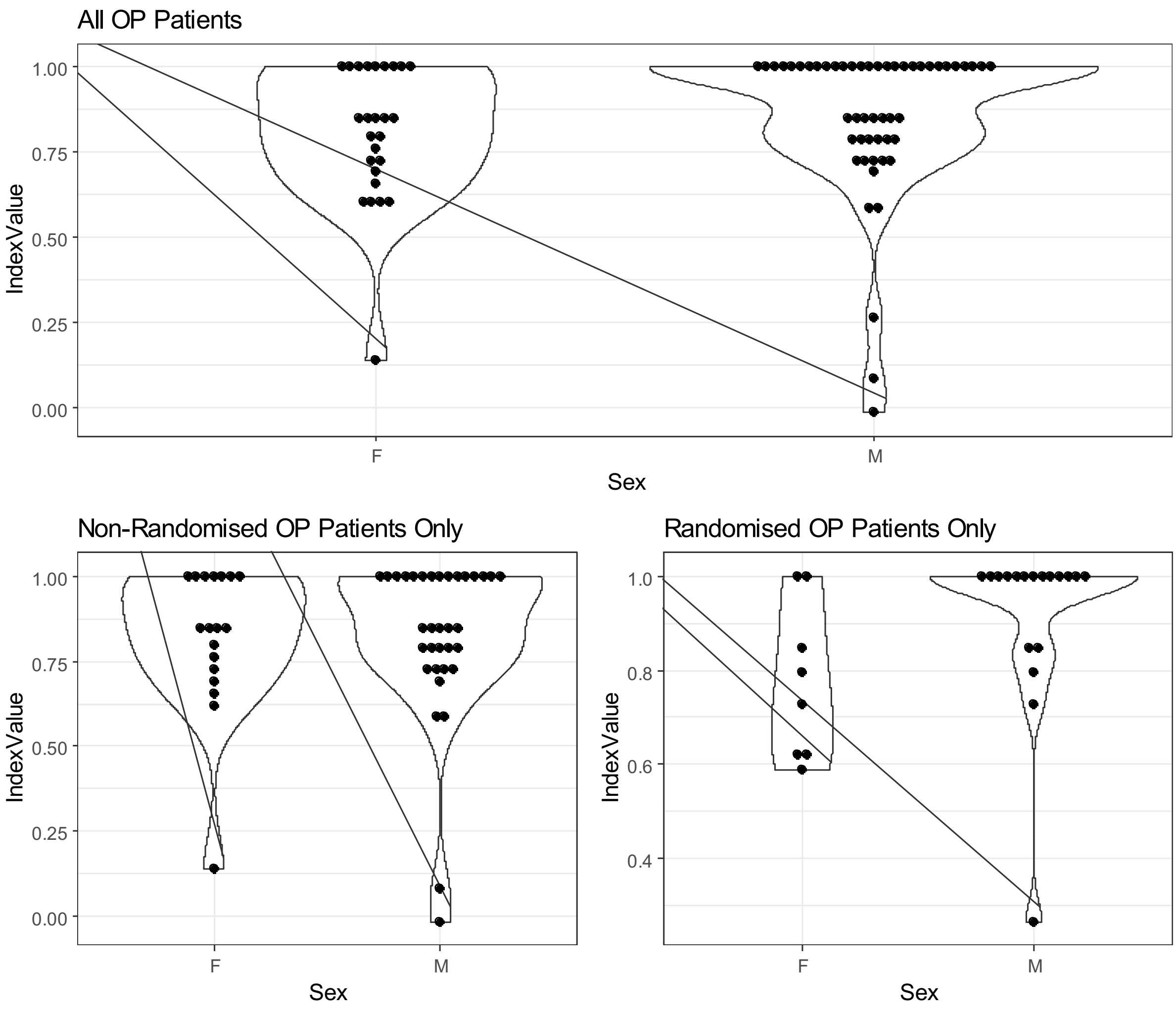

Figure 19: Baseline EQ5D Index Value by sex

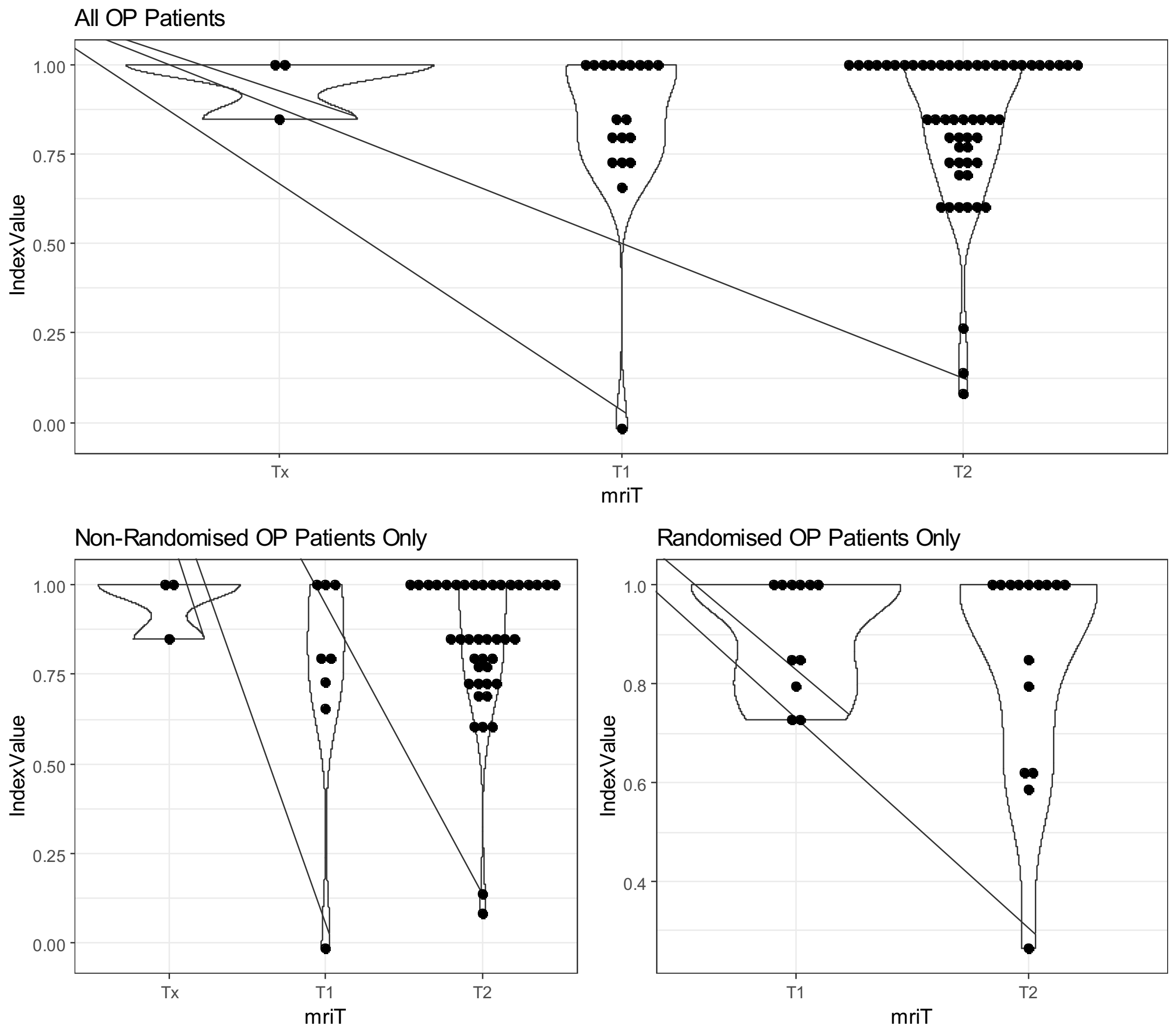

Figure 20: Baseline EQ5D Index Value by MRI T stage

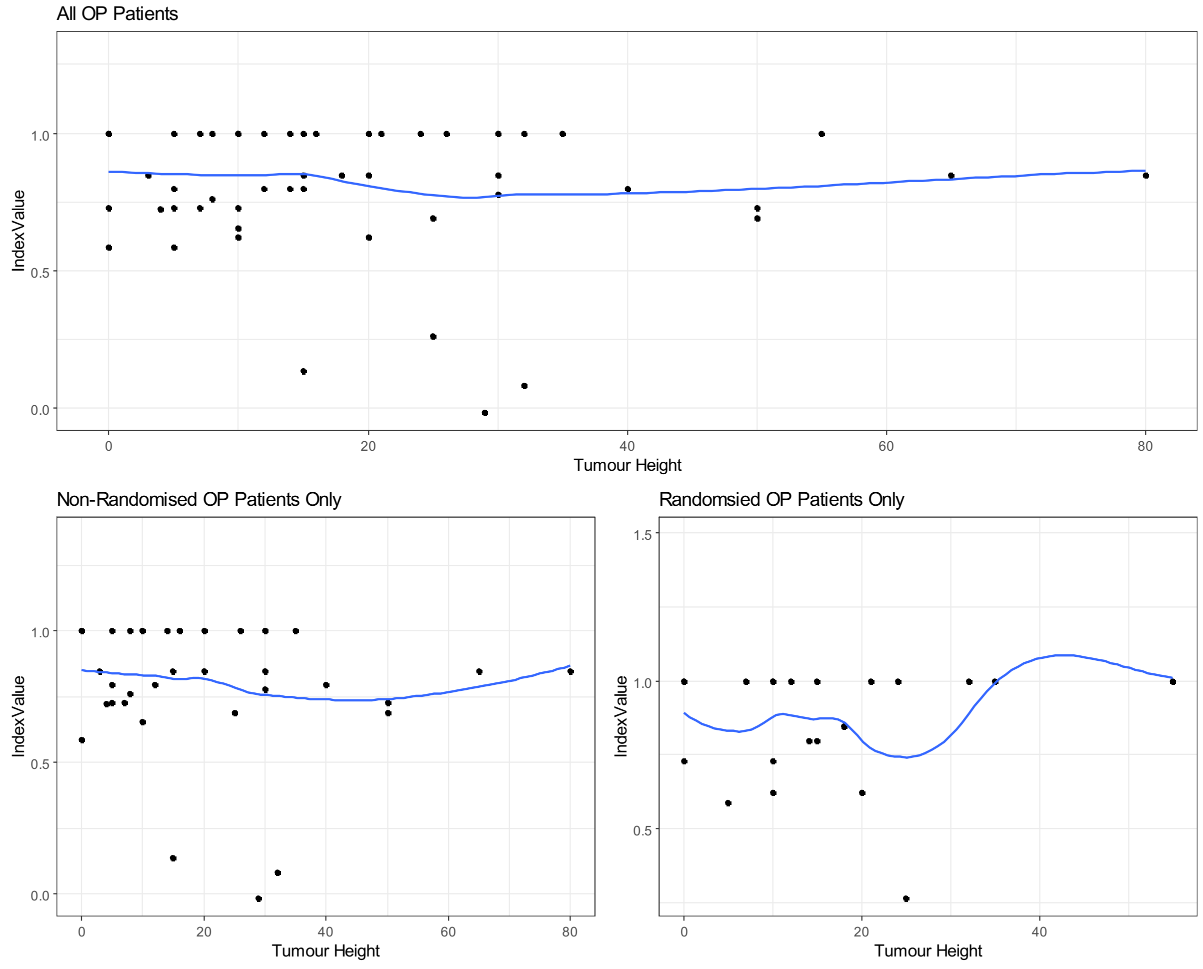

Figure 21: Baseline EQ5D Index Value by Tumour height

Table 4: Results of the Univariate analysis for EQ5D

**Factors**	**Effect estimate**
**EQ5D Index Value**	
Mechanism of entry *(Randomised as the reference category)*	-0.05 (95% CrI: -0.15, 0.15)
Age (years)	-0.01 (95% CrI: -0.01, 0)
Sex *(Female as the reference category)*	0.05 (95% CrI: -0.06, 0.15)
MRI T Staging *(Tx as the reference category)*	T1: -0.08 (95% CrI: -0.26, 0.10)<br>T2: 0.04 (95% CrI: -0.09, 0.18)
Tumour height	0.00 (95% CI: 0.00, 0.00)

Note: CrI – Credible Interval

Review of Longitudinal Scores by Patient Characteristics

EORTC QLQ-C30

Figures 21-25 respectively present all EORTC QLQ-C30 outcomes by mechanism of entry (randomised vs non-randomised, figure 21) patients age at entry to TREC (figure 22), sex (figure 23), T stage (figure 24), and tumour height (figure 25). These figures are presented for the pooled population of all patients.

Visual inspection of figures 21-25 revealed no discernible difference in outcomes dependant on any factor.

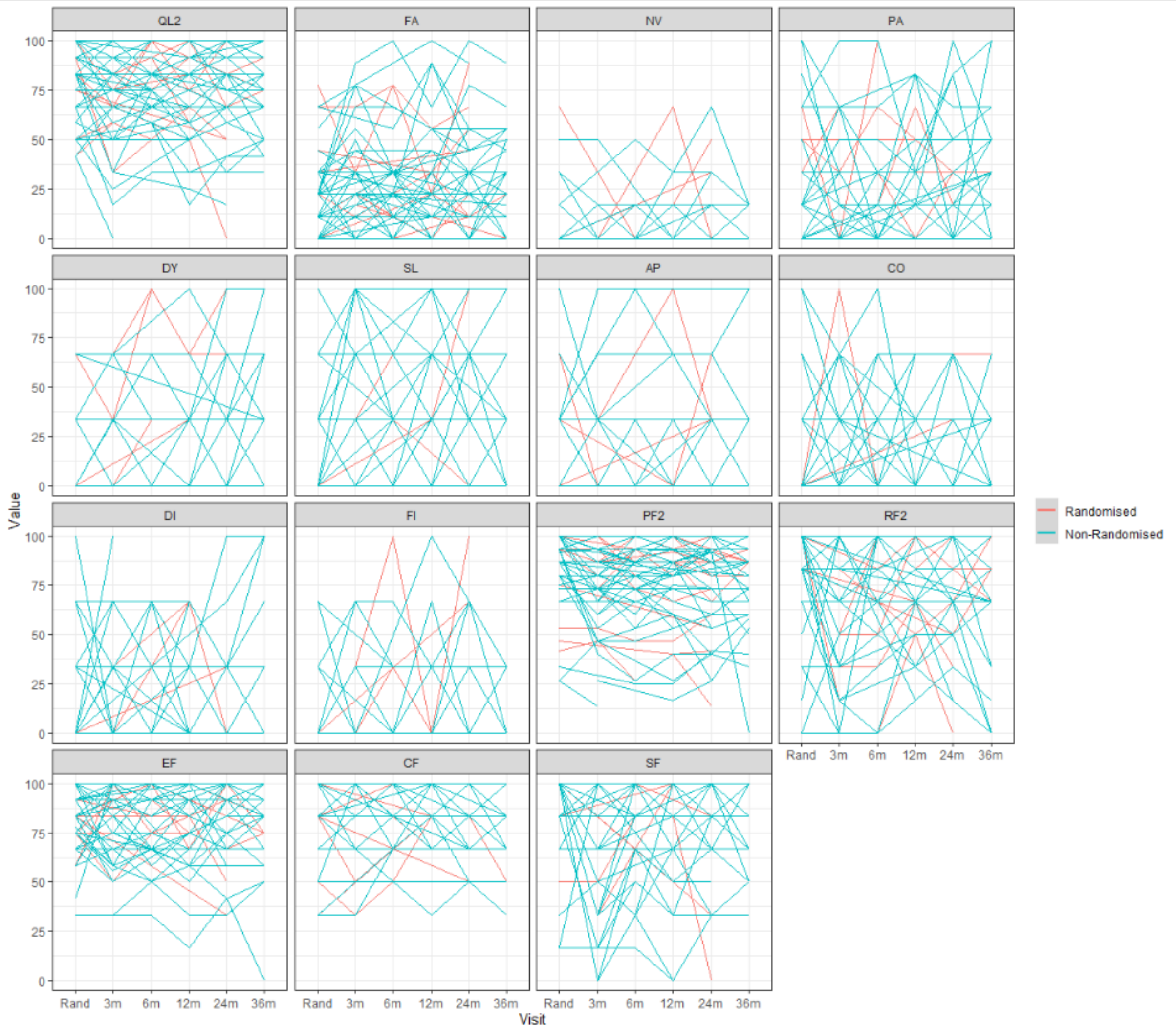

Figure 22: EORTC QLQ-C30 scores by mechanism of entry to TREC

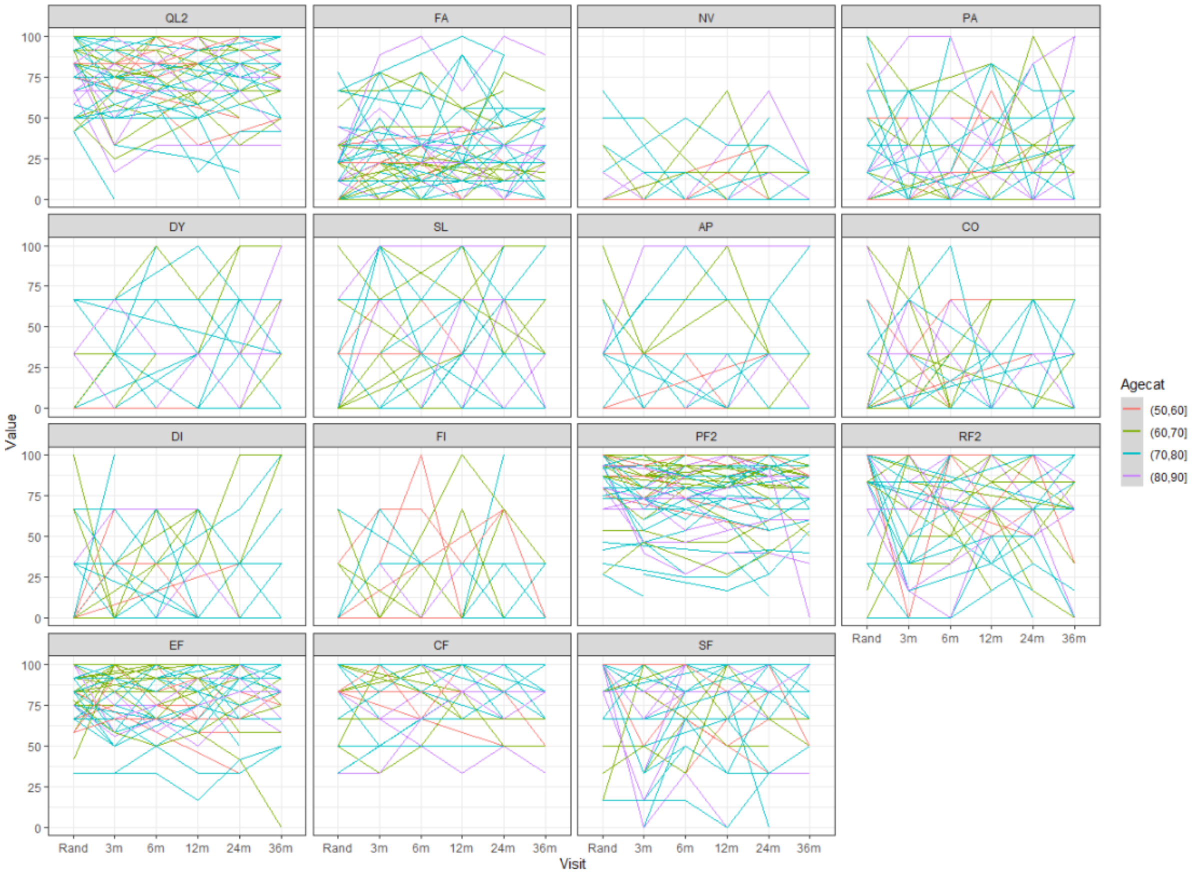

Figure 23: EORTC QLQ-C30 scores by age decile

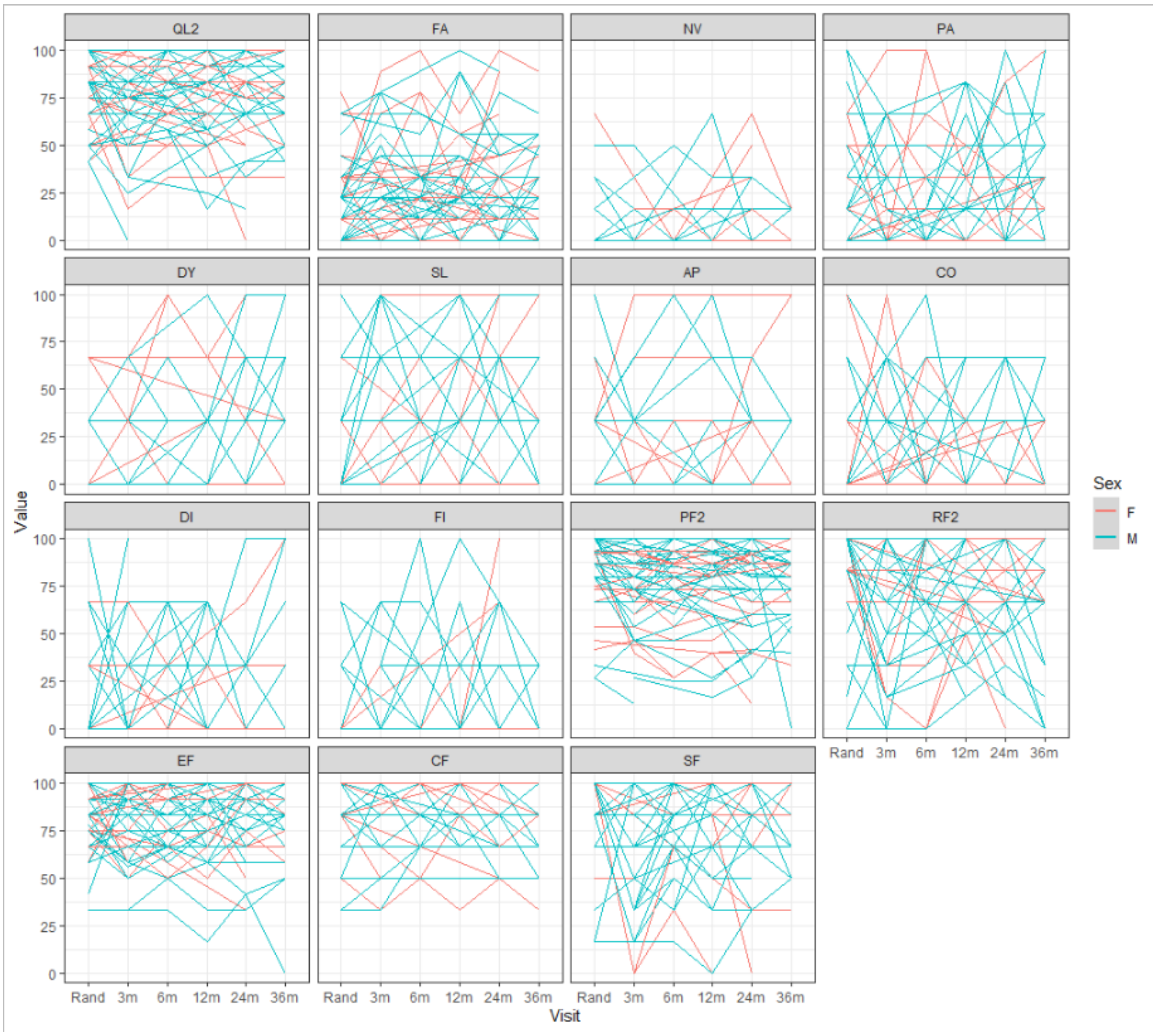

Figure 24: EORTC QLQ-C30 scores by sex

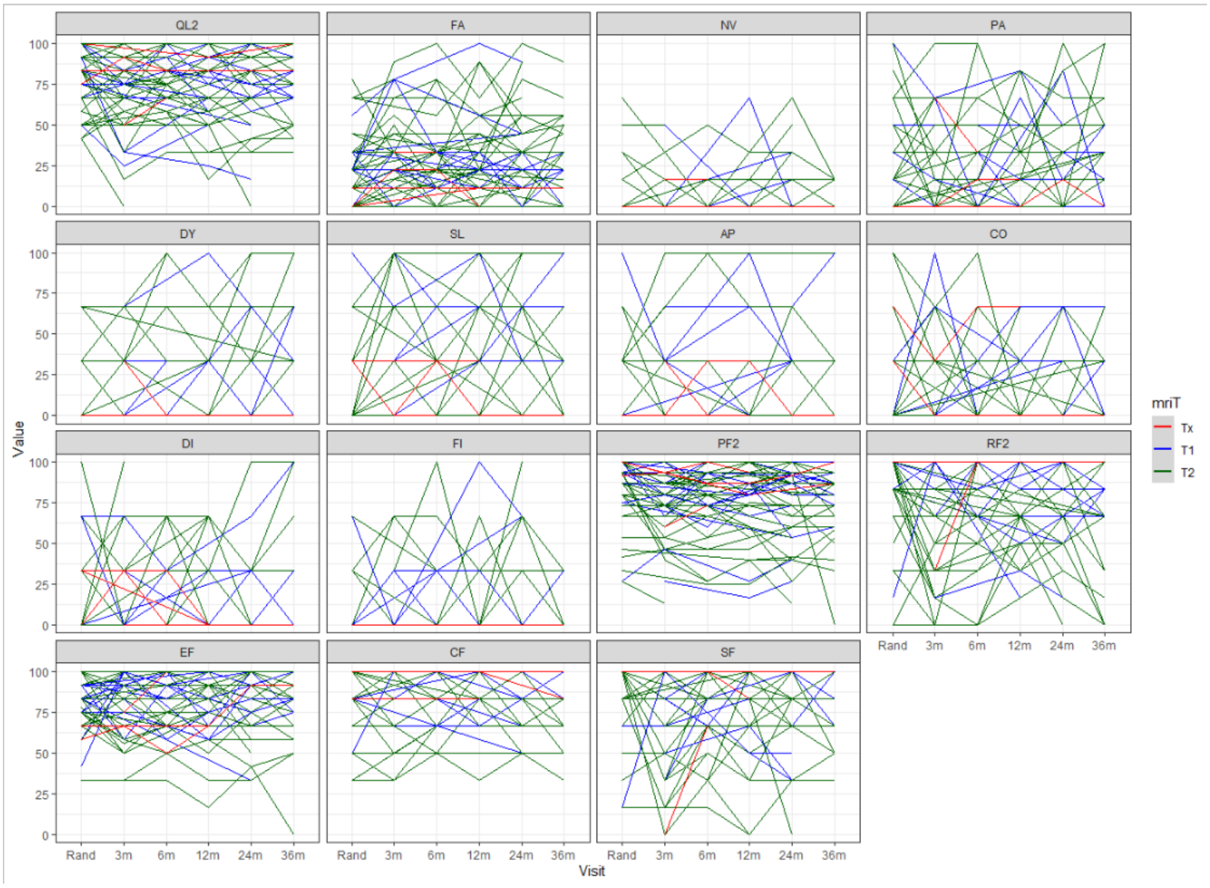

Figure 25: EORTC QLQ-C30 scores by MRI T stage

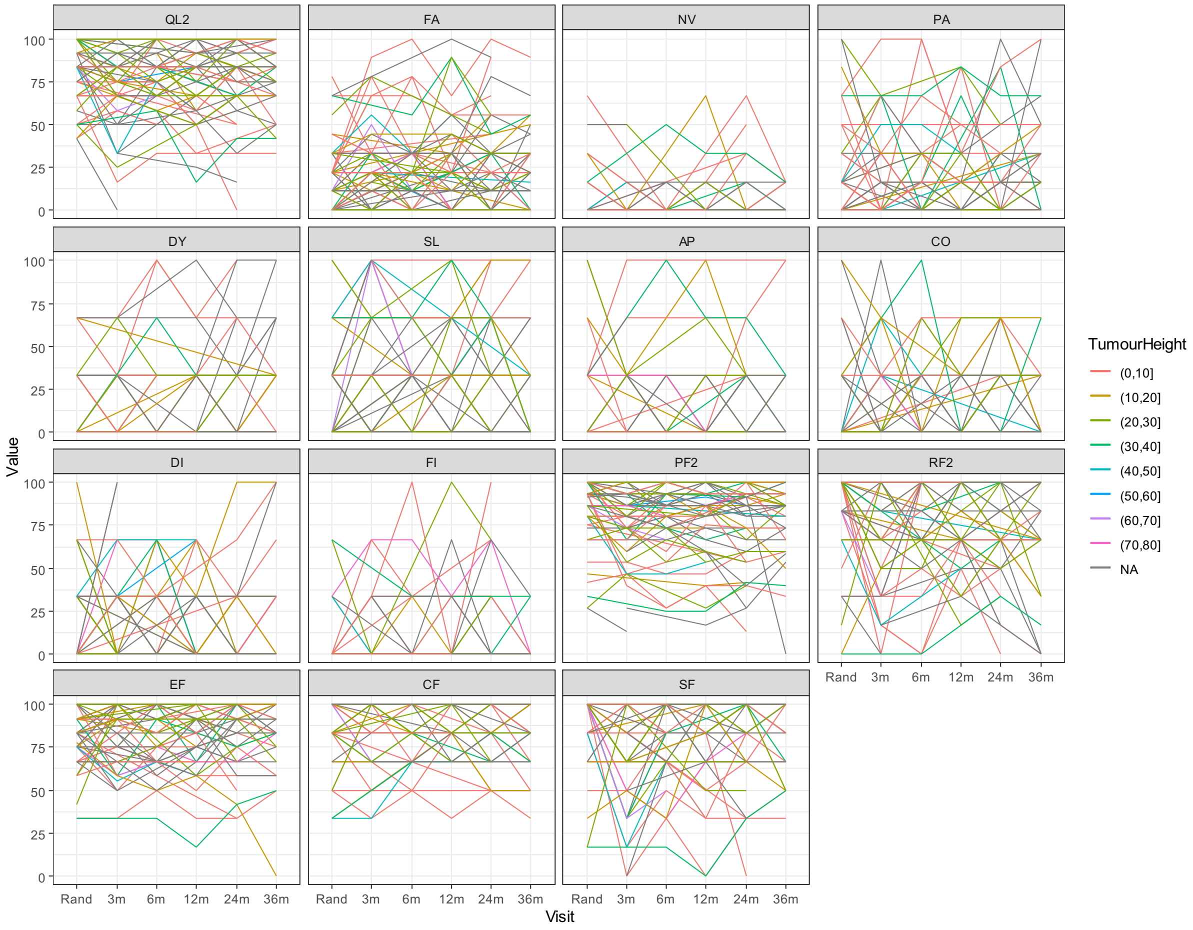

Figure 26: EORTC QLQ-C30 scores by tumour height decile

EORTC QLQ-CR29

Figures 26-30 respectively present all EORTC QLQ-CR29 outcomes by mechanism of entry (randomised vs non-randomised, figure 26) patients age at entry to TREC (figure 27), sex (figure 28), T stage (figure 29), and tumour height (figure 30). These figures are presented for the pooled population of all patients.

Visual inspection of figures 26-30 revealed no discernible difference in outcomes dependant on any factor.

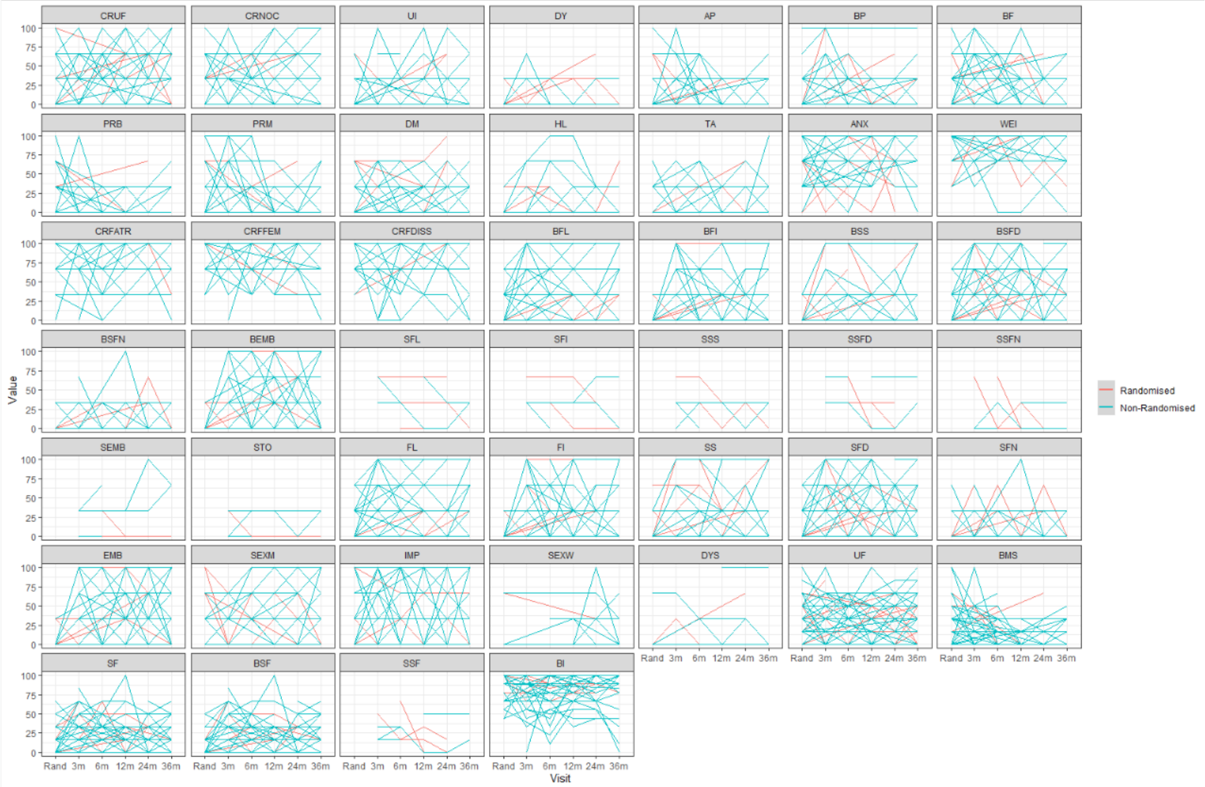

Figure 27: EORTC QLQ-CR29 scores by mechanism of entry to TREC

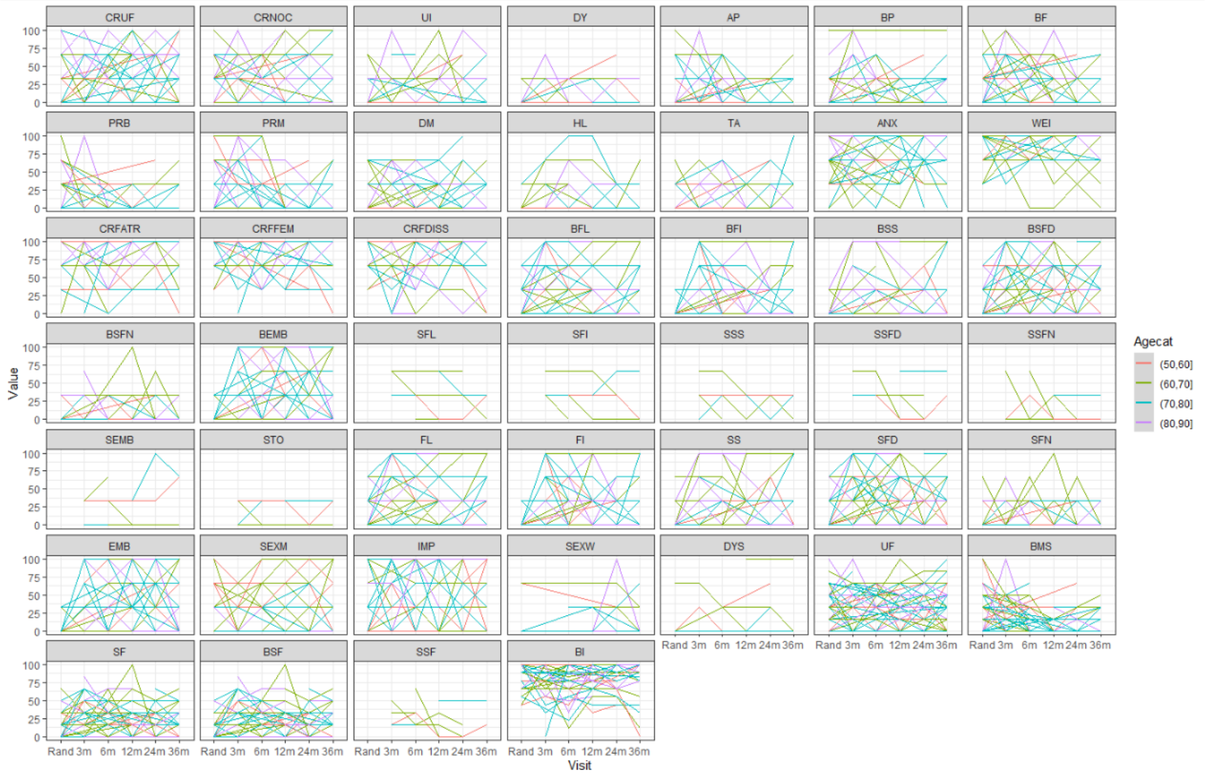

Figure 28: EORTC QLQ-CR29 scores by age decile

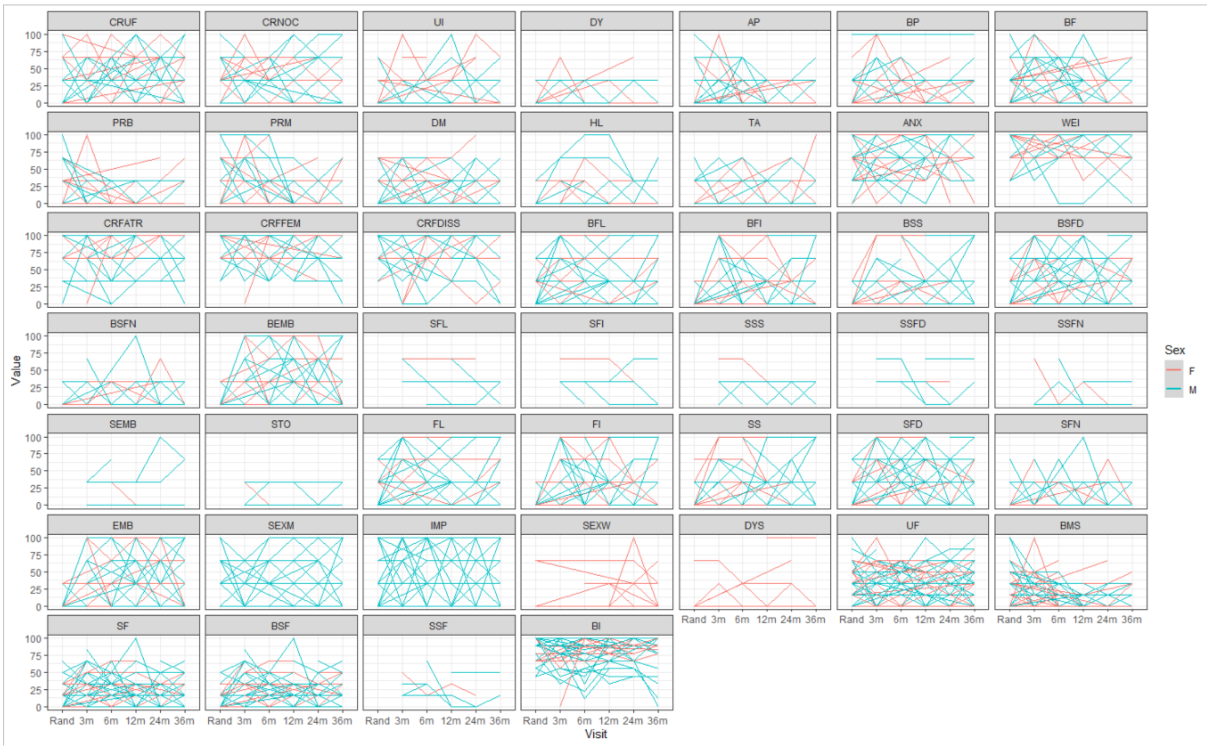

Figure 29: EORTC QLQ-CR29 scores by sex

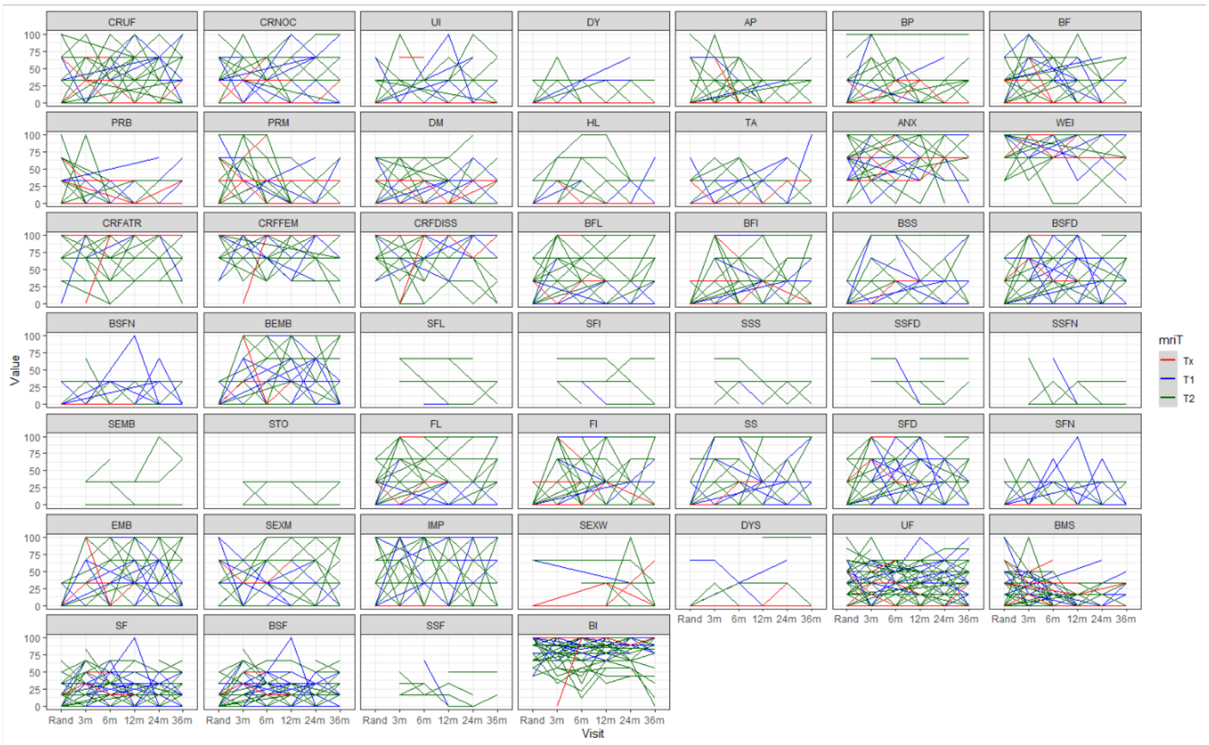

Figure 30: EORTC QLQ-CR29 scores by MRI T stage

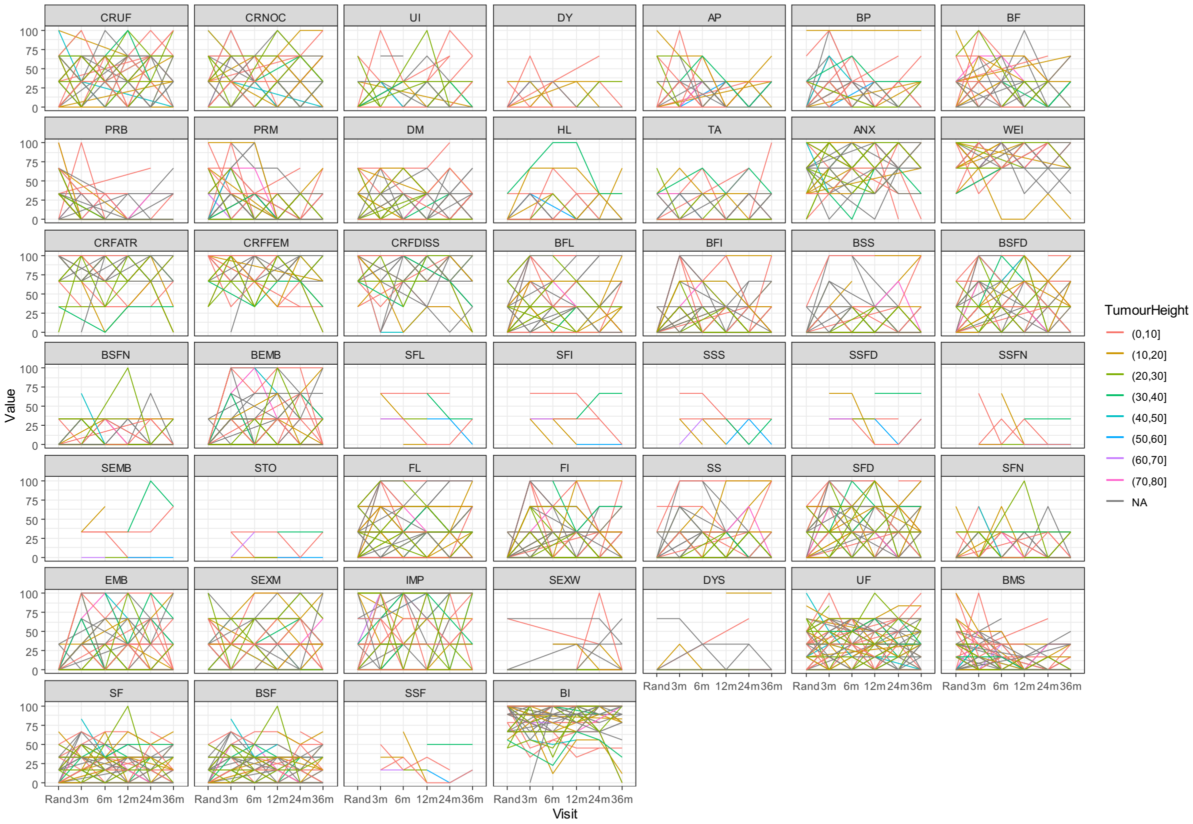

Figure 31: EORTC QLQ-CR29 scores by tumour height decile

COREFO

Figures 31-35 respectively present all COREFO outcomes by mechanism of entry (randomised vs non-randomised, figure 31) patients age at entry to TREC (figure 32), sex (figure 33), T stage (figure 34), and tumour height (figure 35). These figures are presented for the pooled population of all patients. Visual inspection of figures 31-35 revealed no discernible difference in outcomes dependant on any factor.

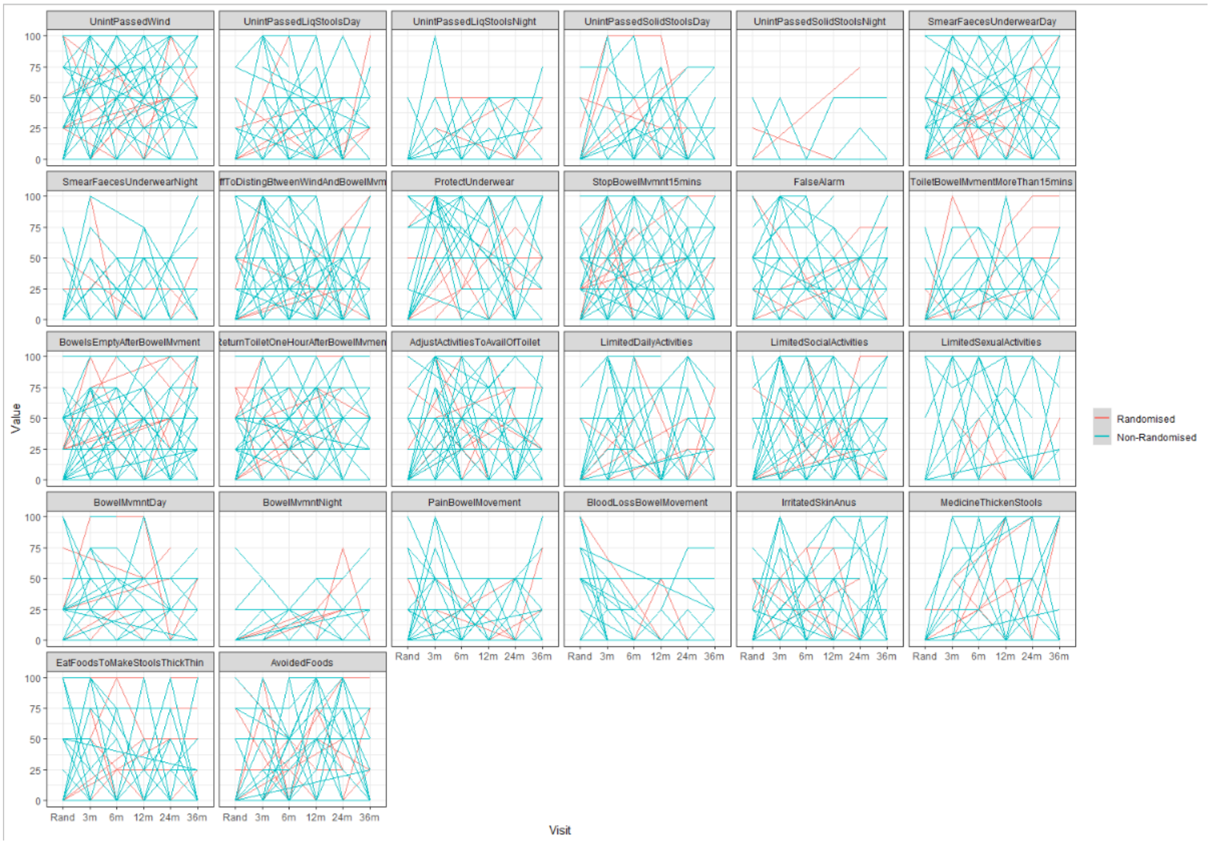

Figure 32: COREFO outcomes by mechanism of entry to TREC

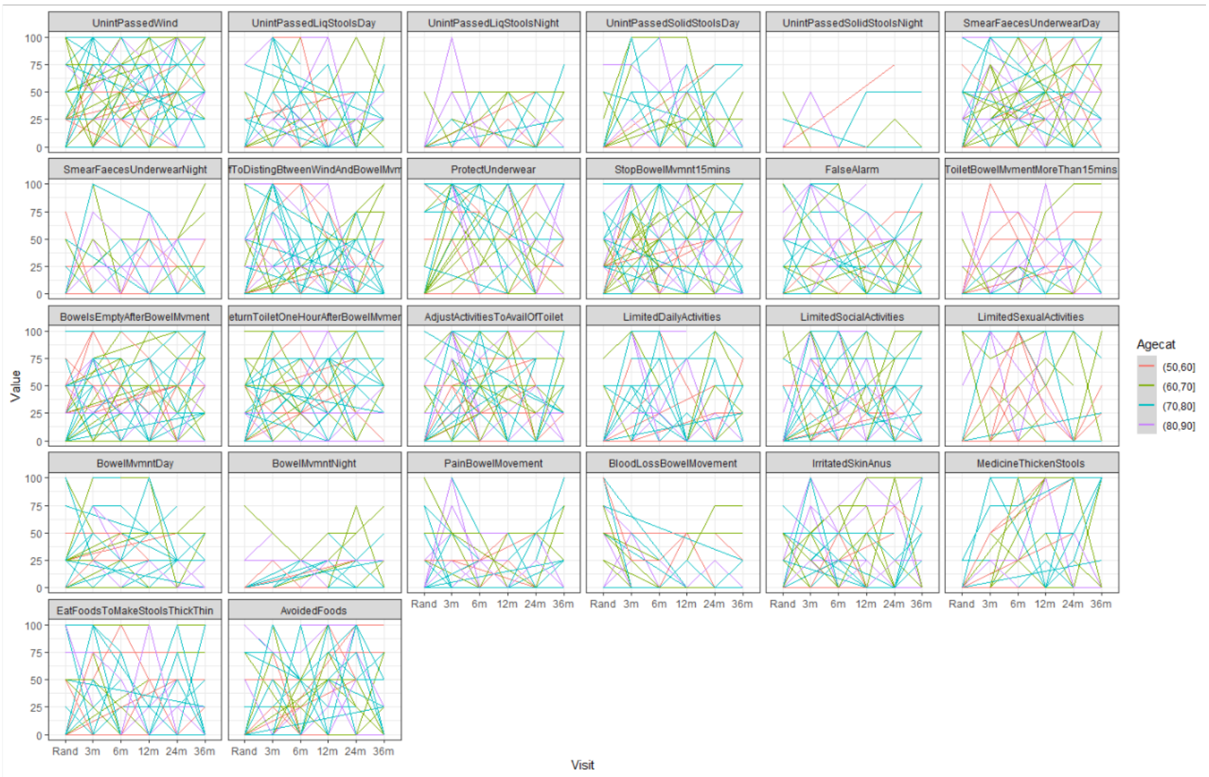

Figure 33: COREFO outcomes by age decile

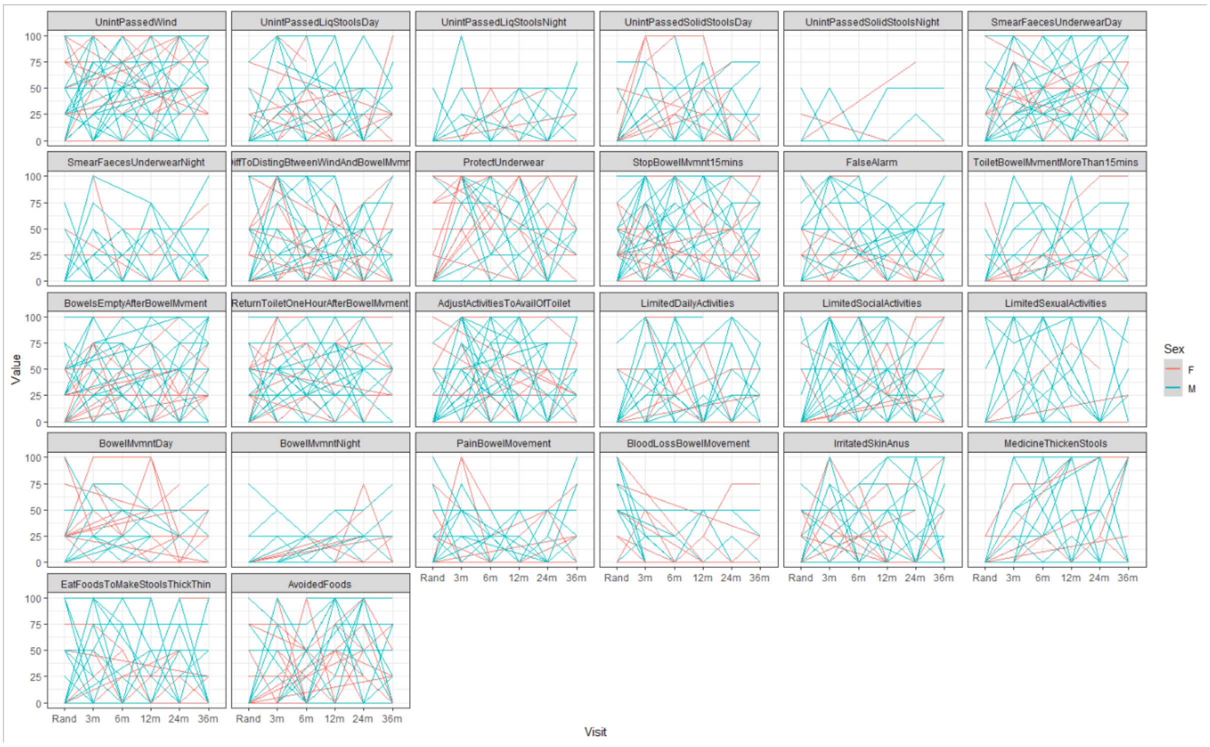

Figure 34: COREFO outcomes by sex

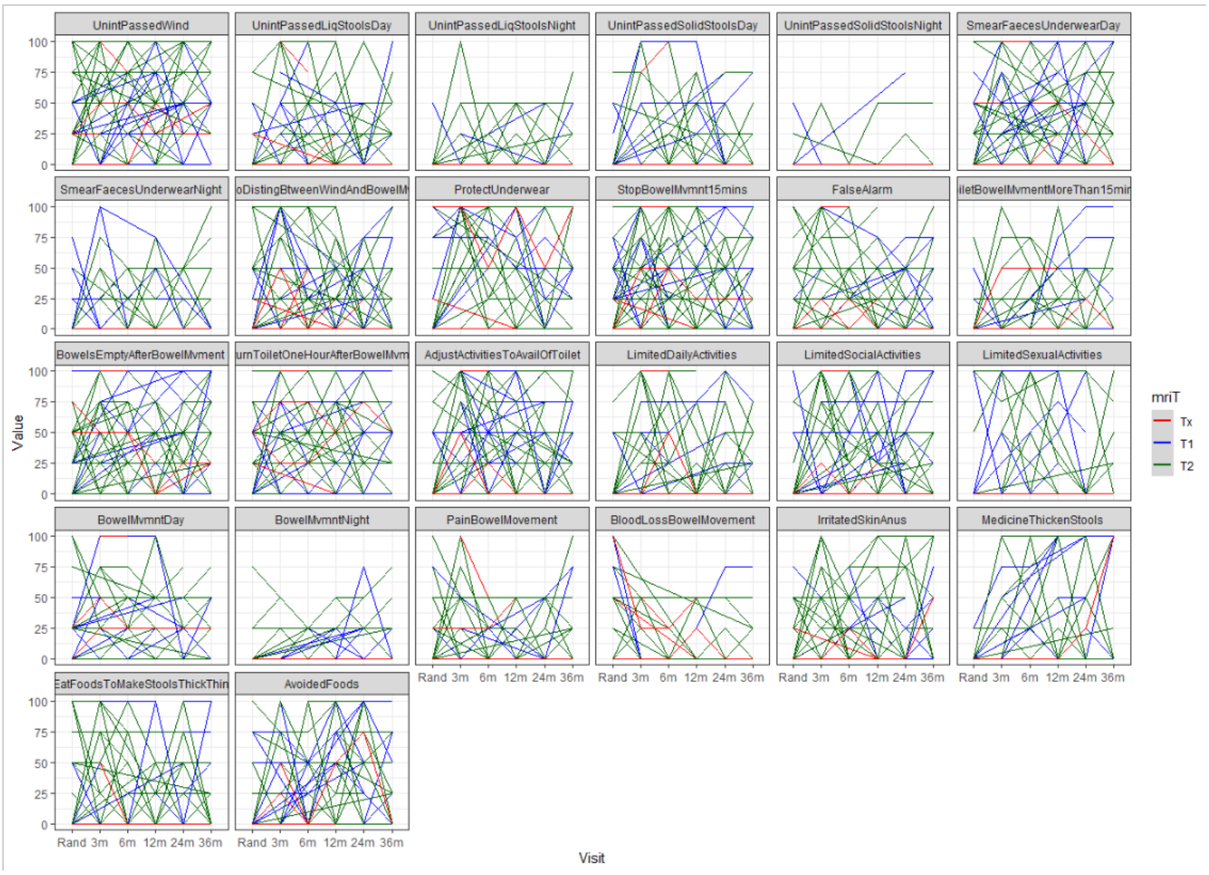

Figure 35: COREFO outcomes by MRI T stage

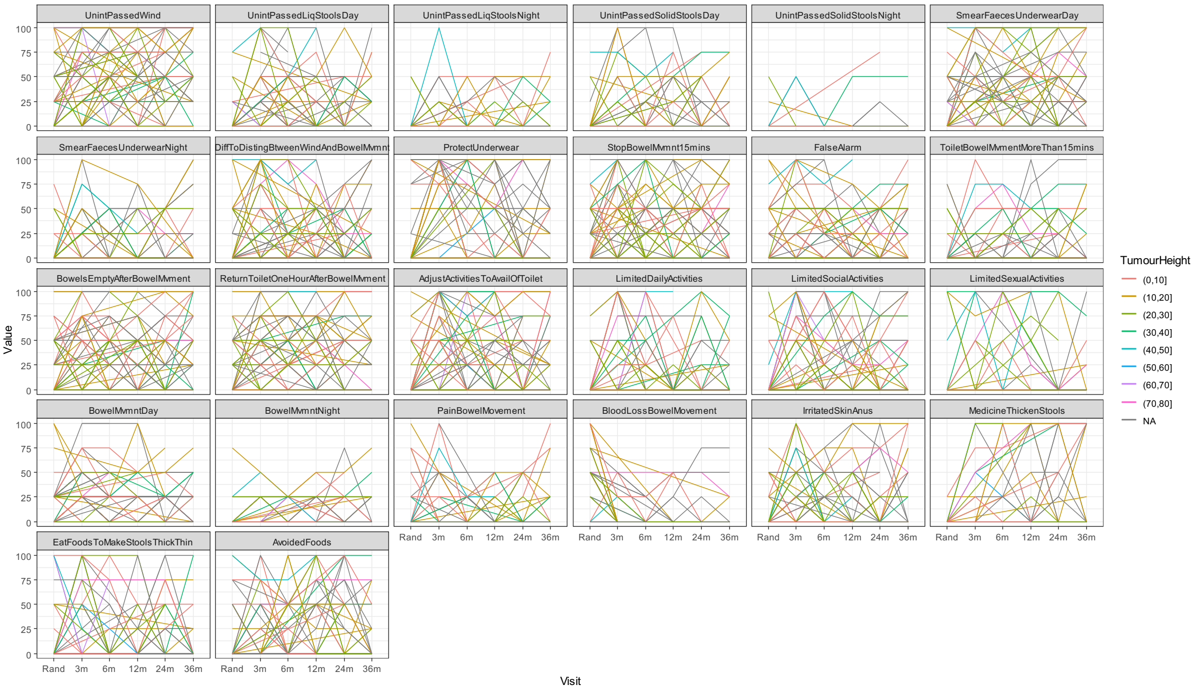

Figure 36: COREFO outcomes by tumour height

EQ-5D Index Value

Figures 36-40 respectively present EQ-5D index values by mechanism of entry (randomised vs non-randomised, figure 36) patients age at entry to TREC (figure 37), sex (figure 38), T stage (figure 39), and tumour height (figure 40). These figures are presented for the pooled population of all patients. Visual inspection of figures 36-40 revealed no discernible difference in outcomes dependant on any factor.

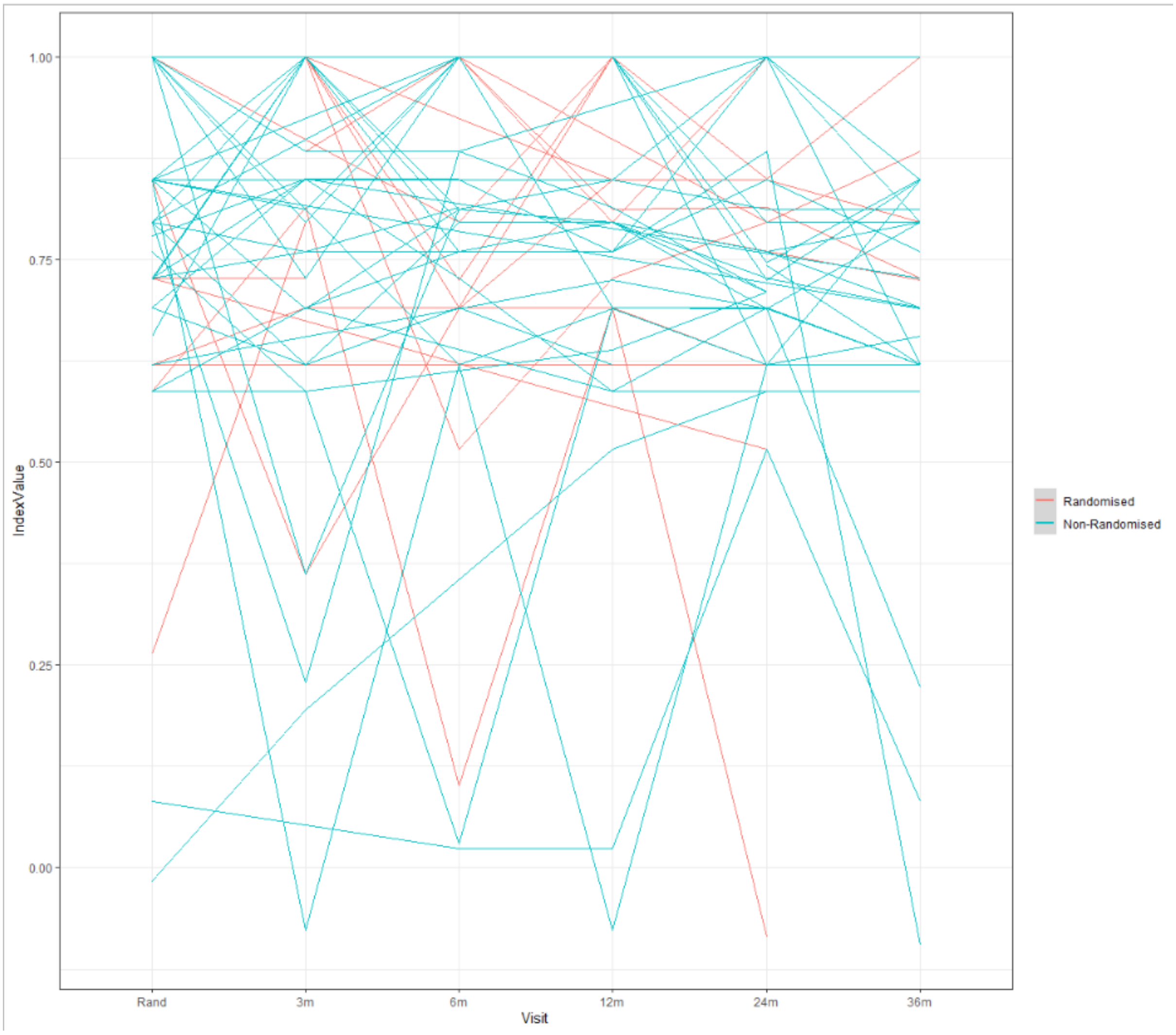

Figure 37: EQ5D Index Value by mechanism of entry to TREC

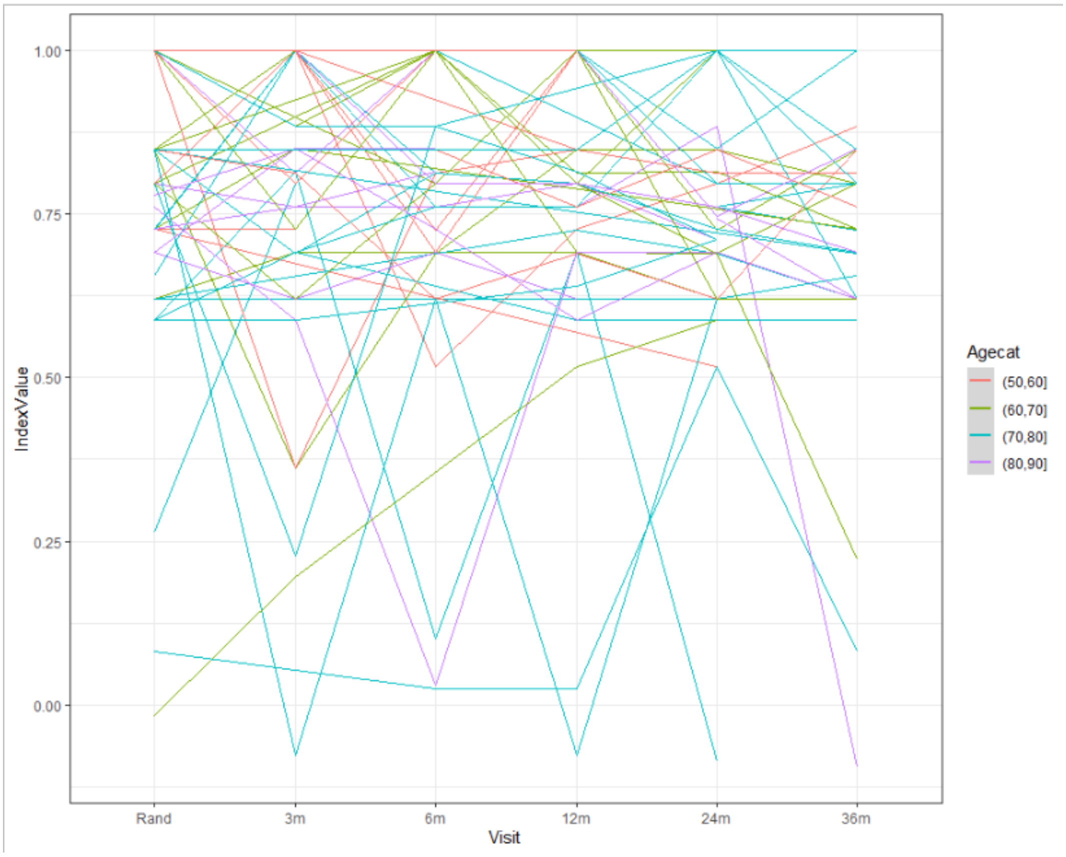

Figure 38: EQ5D Index Value by age decile

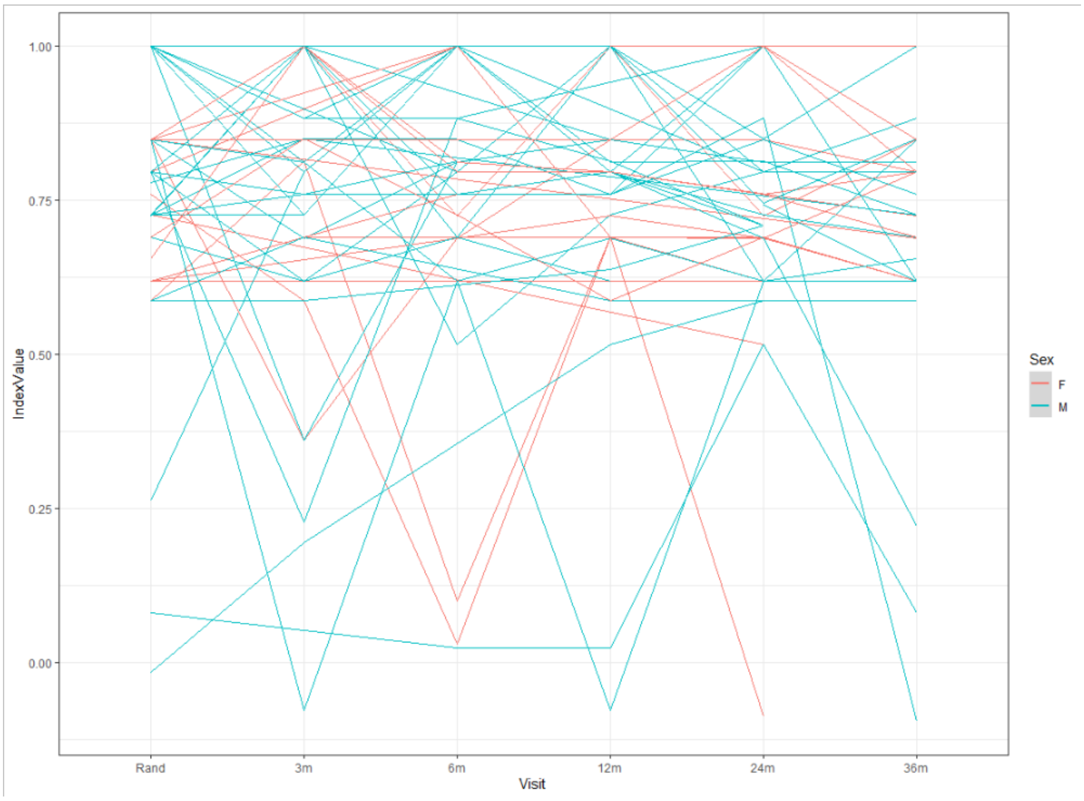

Figure 39: EQ5D Index Value by sex

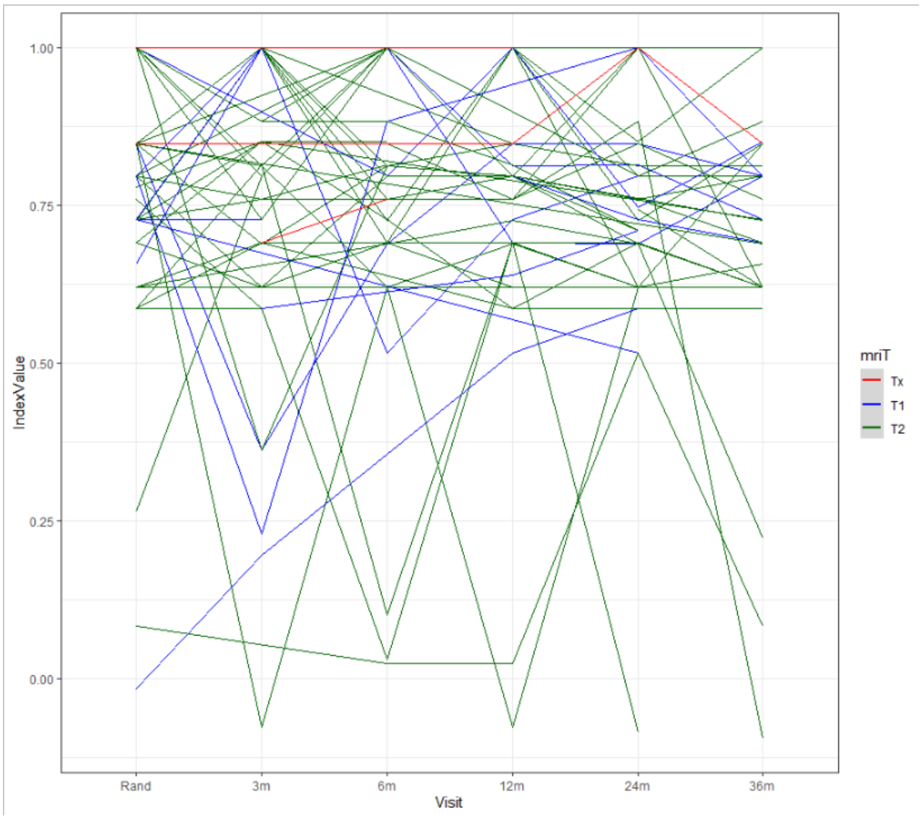

Figure 40: EQ5D Index Value by MRI T Stage

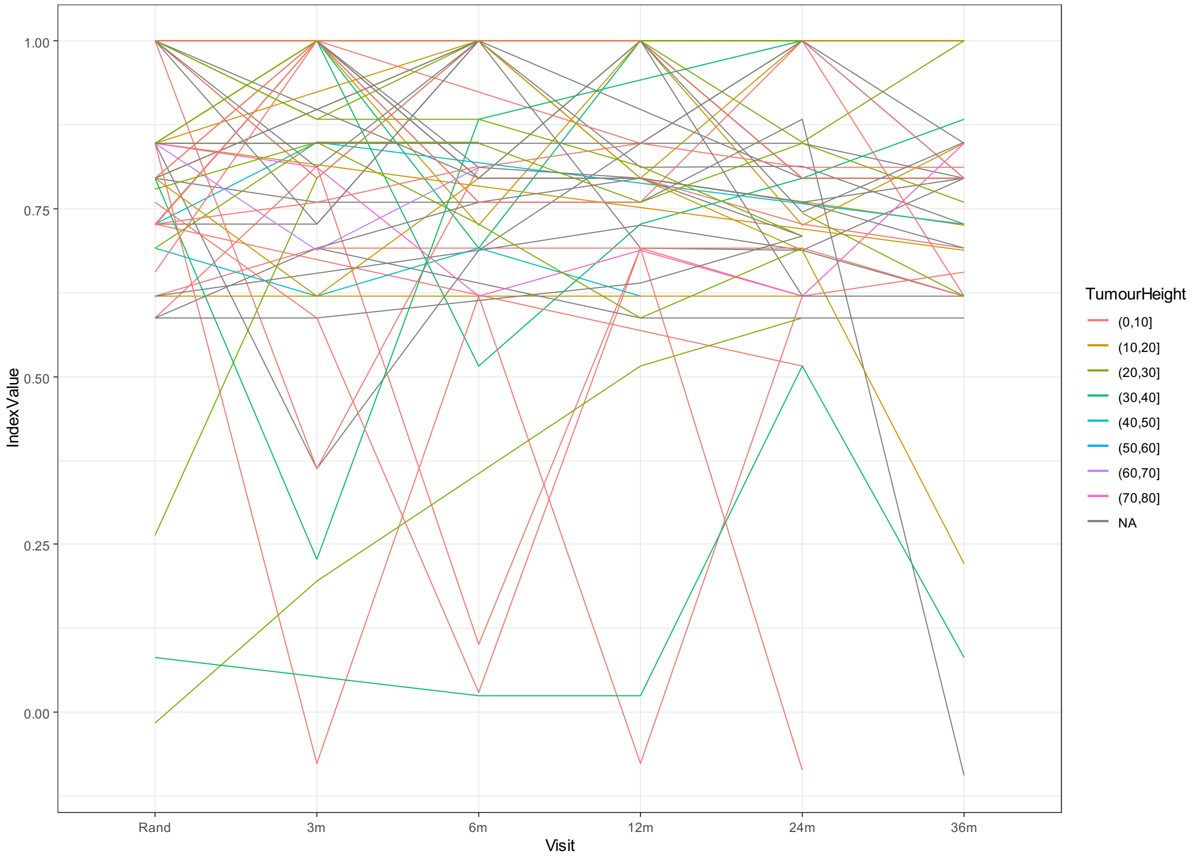

Figure 41: EQ5D index value by tumour height decile

List of Collaborators

First name	Surname
Alan	Beveridge
Bruce	Levy
Kelly	Handley
Gina	Brown
Peter	Antonio
Alex	Vince
Nick	Hilken
Chakanaka	Sidile
Adrian	Wilcockson
Richard	Peto
Tom	Crosby
Brendan	Moran
Julie	Olliff
Katti	Ashok
Simone	Slawik
Andrew	Smethurst
Rajaram	Sripadam
Veena	Tagore
Monica	Terlizzo
Bearn	Philip
Robert	Davies
Susan	Dodd
Sharadah	Essapen
Pasha	Nisar
Alexandra	Stewart
Jonathan	Trickett
Bansal	Ashish
Peter	Billings
Palanichamy	Chandran
Conor	Corr
Edward	Favill
Simon	Gollins
Peter	Marsh
Andrew	Maw
Rakha	Neupane
Ramesh	Rajagopal
Rachel	Cooper
John	Griffith
Paul	Hatfield
Andy	Lowe
Julian	Ostrowski
Jonathan	Robinson
Rhian	Simpson
Richard	Adams
Robert	Bleehen
Michael	Davies
Meleri	Morgan

Darren	Boone
Nicola	Lacey
Ian	Seddon
Bruce	Sizer
Helen	Stunell
Shaobin	Wu
Maher	Hadaki
Dominic	Blunt
Susan	Cleator
Ara	Darzi
Robert	Goldin
Paul	Ziprin
Mike	Dobson
Mark	Pitt
Shabbir	Susnerwala
Deborah	Williamson
Georgina	Howarth
Stephen	Lee
Paul	Wright
Tim	Hoare
Alan	Horgan
Fiona	McDonald
Stephanie	Needham
John	Scott
Timothy	Simmons
Debashis	Biswas
James	Hernon
Gaurav	Kapur
Sandeep	Kapur
James	Sington
Christopher	Speakman
William	Stebbings
Stuart	Williams
Madhavi	Adusumalli
Anil	Agarwal
David	Borowski
Dharmendra	Garg
Talvinder	Gill
Mohammed	Hegab
Catherine	Hobday
Veena	Rao
Jyotsna	Shrimankar
Mohamed	Tabaqchali
David	Wilson
Oliver	Jones
Neil	Mortensen
Andrew	Slater
Aron	Szuts
Lai	Wang
Bryan	Warren

Andrew	Weaver
Mukhtar	Ahmad
Julian	Alexander
Maxine	Flubacher
David	Tarver
Suhail	Baluch
Richard	Beable
David	Cowlishaw
Antony	Higginson
Prokopios	Vogiatzis
Neil	Cruickshank
Howard	Joy
David	Peake
Ulises	Zanetto
Mark	Saunders
Arthur	Sun-Myint
Rajaram	Sripadam
Rachel	Cooper
Paul	Hatfield
Mark	Teo
Arthur	Allan
Ian	Geh
John	Glaholm
Mark	Goldstein
Rahul	Hejmadi
Gerald	Langman
Dion	Morton
Cyril	Nelson
Deborah	Tattersall
Stephen	Falk
Robert	Longman
Huw	Roach
Jamshed	Shabbir
Golda	Shelley-Fraser
Michael	Thomas
Neil	Cripps
Yasser	Haba
Guy	Harris
Max	Hookway
Jay	Simson
Angela	Skull
Tijani	Umar
